# Design, synthesis, molecular docking, and in vitro* α*-glucosidase inhibitory activities of novel 3-amino-2,4-diarylbenzo[4,5]imidazo[1,2-*a*]pyrimidines against yeast and rat *α*-glucosidase

**DOI:** 10.1038/s41598-021-91473-z

**Published:** 2021-06-07

**Authors:** Fariba Peytam, Ghazaleh Takalloobanafshi, Toktam Saadattalab, Maryam Norouzbahari, Zahra Emamgholipour, Setareh Moghimi, Loghman Firoozpour, Hamid Reza Bijanzadeh, Mohammad Ali Faramarzi, Somayeh Mojtabavi, Parviz Rashidi-Ranjbar, Saeed Karima, Roya Pakraad, Alireza Foroumadi

**Affiliations:** 1 Drug Design and Development Research Center, The Institute of Pharmaceutical Sciences (TIPS), Tehran University of Medical Sciences, Tehran, Iran; 2 School of Chemistry, College of Science, University of Tehran, Tehran, Iran; 3 Department of Medicinal Chemistry, Faculty of Pharmacy, Tehran University of Medical Sciences, Tehran, Iran; 4 Faculty of Medicine, Eastern Mediterranean University, via Mersin 10, Famagusta, Northern Cyprus Turkey; 5 Department of Environmental Sciences, Faculty of Natural Resources and Marine Sciences, Tarbiat Modares University, Tehran, Iran; 6 Department of Pharmaceutical Biotechnology, Faculty of Pharmacy, Tehran University of Medical Sciences, Tehran, Iran; 7 Department of Clinical Biochemistry, School of Medicine, Shahid Beheshti University of Medical Sciences (SBMU), Tehran, Iran

**Keywords:** Structure-based drug design, Biocatalysis

## Abstract

In an attempt to find novel, potent α-glucosidase inhibitors, a library of poly-substituted 3-amino-2,4-diarylbenzo[4,5]imidazo[1,2-*a*]pyrimidines **3a–ag** have been synthesized through heating a mixture of 2-aminobenzimidazoles **1** and *α*-azidochalcone **2** under the mild conditions. This efficient, facile protocol has been resulted into the desirable compounds with a wide substrate scope in good to excellent yields. Afterwards, their inhibitory activities against yeast α-glucosidase enzyme were investigated. Showing IC_50_ values ranging from 16.4 ± 0.36 µM to 297.0 ± 1.2 µM confirmed their excellent potency to inhibit α-glucosidase which encouraged us to perform further studies on α-glucosidase enzymes obtained from rat as a mammal source. Among various synthesized 3-amino-2,4-diarylbenzo[4,5]imidazo[1,2-*a*]pyrimidines, compound **3k** exhibited the highest potency against both *Saccharomyces cerevisiae α*-glucosidase (IC_50_ = 16.4 ± 0.36 μM) and rat small intestine *α*-glucosidase (IC_50_ = 45.0 ± 8.2 μM). Moreover, the role of amine moiety on the observed activity was studied through substituting with chlorine and hydrogen resulted into a considerable deterioration on the inhibitory activity. Kinetic study and molecular docking study have confirmed the in-vitro results.

## Introduction

Diabetes mellitus is a common, chronic disease mainly characterized by the body’s lack of ability to control blood sugar resulted into chronic hyperglycemia. Progression in this metabolic disorder may bring subsequent severe health problems, including abnormally great thrust, excessive appetite, overweight, blindness, excessive urination, leg amputation, cardiovascular complications, as well as renal and neurodegenerative diseases^1–4^. According to the World Health Organization (WHO) report, diabetes is increasing with alarming rate worldwide. While 415million people have become infected in 2015, this figure would reach 700 million in 2045^5^. Diabetes is classically classified into three groups: type I diabetes mellitus (T1DM), type II diabetes mellitus (T2DM), and gestational diabetes mellitus (GDM), among which T2DM is the most prevalent^6–8^.

To treat T2DM, traditional medications are reducing the hepatic glucose production, increasing the insulin action and its secretion from *β*-pancreatic cells, and controlling the digestive carbohydrate enzyme activities^9^. Carbohydrate digestive enzymes, found in the brush border of the intestine, play the catalyzing role in breaking down the long-chain polysaccharides into absorbable monosaccharide units. Among these enzymes, *α*-glucosidase has received considerable attention regarding their noticeable role in the lysis of *α*-glucopyranoside bond in oligosaccharides and disaccharides. The released monosaccharide would increase the postprandial blood glucose levels. Accordingly, *α*-glucosidase inhibitors preventing the carbohydrate digestion and glucose release in bloodstream efficiently control T2DM^10^. Acarbose, miglitol, voglibose, and deoxynojirimycin have been clinically used to bind reversibly to *α*-glucosidase and to interrupt the saccharide hydrolysis^11^. Various side effects associated with these drugs, including nausea, bloating, diarrhea, abdominal pain, and flatulence^12^ have been observed; therefore, providing more potent, less toxic *α*-glucosidase inhibitors is highly demanding.

**Figure 1:**
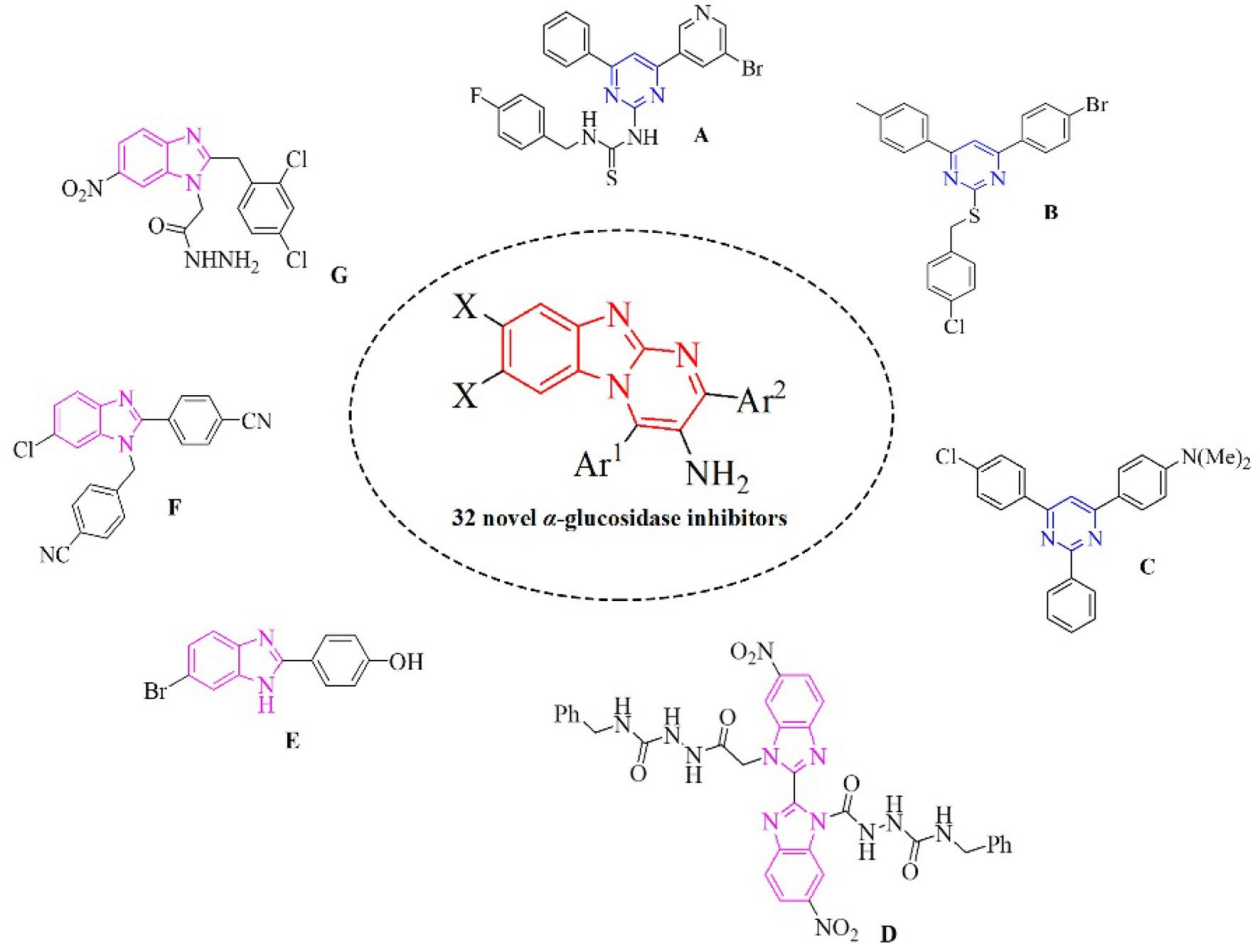
Design of new 3-amino-2,4-diarylbenzo[4,5]imidazo[1,2-*a*]pyrimidine derivatives **3a-ag** as novel *α*-glucosidase inhibitor. *This figure was drawn by ChemDraw Professional 16.0 (https://perkinelmer-chemdraw-professional.software.informer.com).

Over recent decade, various heterocyclic-based compounds possessing *α*-glucosidase inhibitory activities have been found^13–24^. For example, several pyrimidine derivatives have shown excellent inhibition potency^25–28^. Moreover, compounds containing benzimidazole have become an emerging anti-diabetic scaffold during recent years^29–34^. Although there are several reports concerning *α*-glucosidase inhibitors having benzimidazole and pyrimidine skeletons separately, compounds bearing both of these heterocycles, benzo[4,5]imidazo[1,2-*a*]pyrimidine, in particular, as anti-diabetic agents have not been proposed yet (Fig. 1). Therefore, design and synthesis of these targeted compounds which are anticipated to possess potent *α*-glucosidase inhibitory activity could be an interesting challenge in medicinal chemistry.

Benzo[4,5]imidazo[1,2-*a*]pyrimidines, one of the important fused pyrimidine families, have exhibited various significant biological activities, including anticancer^35^, anti-tuberculosis^36^, adenosine receptor inhibitory^37^, anti-inflammatory^38^, antimicrobial^39,40^, calcium channel blocking^41^, antiviral^42^, as well as anti- neurodegenerative properties^43^. As a privileged scaffold, several synthetic approaches toward substituted benzo[4,5]imidazo[1,2-*a*]pyrimidines have already been reported. Among them, the reactions of 2-aminobenzimidazole with appropriate electrophilic compounds are the most traditional routes. Some noticeable examples include the reaction of this starting material with *α*,*β*-unsaturated compounds^44^, 1,2-diphenylethanones, alkynes, or 1,3-bis electrophilic compounds and aromatic aldehydes^45–50^, acrylamides bearing a leaving group like ethoxy in the *β*-position^51^, domino reaction with N-methyl-1-(methylthio)-2-nitroprop-1-en-1-amine and aromatic aldehydes^52^, as well as four-component reaction with amines, diketene, and aromatic aldehydes^53^.

**Scheme 1:**
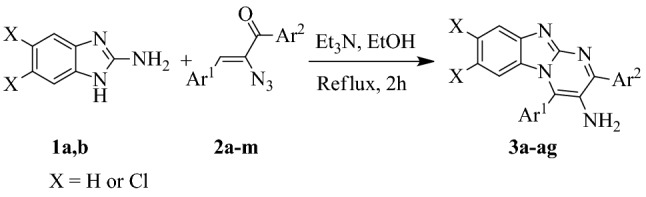
Synthesis of 3-amino-2,4-diarylbenzo[4,5]imidazo[1,2-*a*]pyrimidine derivatives **3a–ag**.

**Scheme 2:**
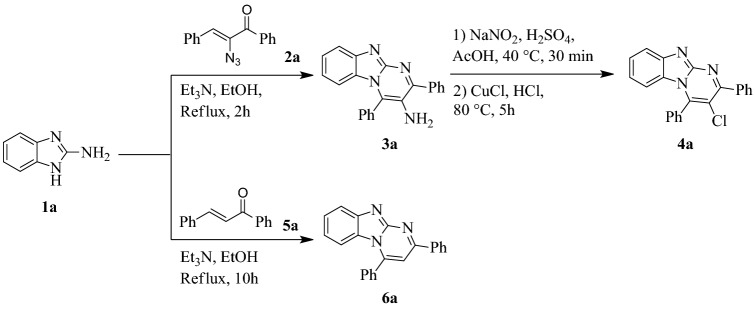
Synthesis of substituted-2,4-diarylbenzo[4,5]imidazo[1,2-*a*]pyrimidine derivatives **4a** and **6a**.

Although various synthetic methods for benzo[4,5]imidazo[1,2-*a*]pyrimidines have been reported, the reaction between 2-aminobenzimidazoles **1** and *α*-azidochalcones **2** has not been proposed yet. Considering the significant role of *α*-glucosidase inhibitors in current pharmaceutical science, in present study, we focused on the synthesis of novel series poly-substituted 3-amino-2,4-diarylbenzo[4,5]imidazo[1,2-*a*]pyrimidines **3** and subsequently, the evaluation of their inhibitory activity against *α*-glucosidase (Scheme 1). To achieve this goal, a targeted Michael addition–cyclization of 2-aminobenzimidazoles **1** with *α*-azidochalcones **2** has been performed to obtain our desirable compounds **3**. Moreover, to highlight the role of amine in the anti-*α*-glucosidase activities, this moiety has been substituted with chlorine and hydrogen (compounds **4a** and **6a**, respectively), both of which showed considerably less potency (Scheme 2).

## Results and discussion

### Chemistry

In this paper, an efficient, facile synthetic approach including Michael-addition-cyclization of 2-aminobenzimidazole **1** with *α*-azidochalcone **2** has been applied to obtain a library of 3-amino-2,4-diarylbenzo[4,5]imidazo[1,2-*a*]pyrimidines **3a–ag**. It is worth mentioning that over recent decade, *α*-azidochalcones **2** have been widely utilized to synthesize several aza-heterocycles^54–66^. To probe the generality of the proposed reaction, a mixture of 2-aminobenzimidazoles **1a,b**, *α*-azidochalcones **2a–m** (with electron-donating alkyl or methoxy groups as well as electron-withdrawing chlorine or bromine substituted phenyl, and heteroaryl substituents), and Et_3_N in EtOH were heated under the reflux conditions for 2 h. TLC and ^1^H NMR analysis of the reaction mixture confirmed the formation of desirable 3-amino-2,4-diarylbenzo[4,5]imidazo[1,2-*a*]pyrimidines **3** in good to excellent yields (Scheme 1).

To study the role of amine functional group in *α*-glucosidase inhibition, this moiety has been replaced by chlorine (3-chloro-2,4-diphenylbenzo[4,5]imidazo[1,2-*a*]pyrimidine **4a**) and hydrogen (2,4-diphenylbenzo[4,5]imidazo[1,2-*a*]pyrimidine **6a**). Therefore, through sandmayer reaction, a mixture of concentrated sulfuric acid and sodium nitrite was treated with compound **3a** to afford corresponding diazonium salt which went through chlorination reaction using cuprous chloride (CuCl) in concentrated hydrochloric acid. On the other hand, heating a mixture of 2-aminobenzimidazoles **1** and chalcone **5a** in the presence of Et_3_N in EtOH for approximately 10 h afforded 2,4-diphenylbenzo[4,5]imidazo[1,2-*a*]pyrimidine **6a** (Scheme 2).

The structures of the isolated products (**3a–ag**, **4a**, and **6a**) were deduced on the basis of their IR, ^1^H- and ^13^C-NMR spectroscopy, as well as mass spectrometry. Partial assignments of these resonances are provided in the Experimental Part.

**Scheme 3:**
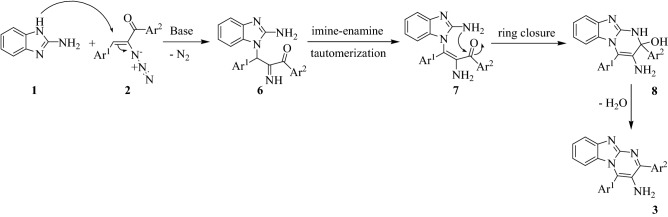
Proposed reaction mechanism for the formation of 3-amino-2,4-diarylbenzo[4,5]imidazo[1,2-*a*]pyrimidine derivatives **3**.

A plausible mechanism for the formation of 3-amino-2,4-diarylbenzo[4,5]imidazo[1,2-*a*]pyrimidines **3** was outlined in Scheme 3. The reaction may be initiated by Michael addition of 2-aminobenzimidazole **1** activated by Et_3_N to *α*-azidochalcone **2** by removal of nitrogen molecule to form adduct **6**, followed by an imine-enamine tautomerization (intermediate **7**). Afterwards, carbonyl functionality can undergo an intramolecular nucleophilic addition of amine moiety resulted from 2-aminobenzimidazole to cyclize the bicyclic skeleton **8**, which may go through dehydration to afford desirable poly substituted benzo[4,5]imidazo[1,2-*a*]pyrimidine **3**.

### Pharmacology

#### In vitro α-glucosidase inhibitory activity

Various 3-amino-2,4-diarylbenzo[4,5]imidazo[1,2-*a*]pyrimidine derivatives **3a–ag** were synthesized to evaluate their *Saccharomyces cerevisiae α*-glucosidase inhibitory activities (Table 1). Results revealed that all targeted compounds exhibited good to excellent inhibitory activities (with IC_50_ values from 16.4 ± 0.36 μM to 297.0 ± 1.2 μM) in comparison to the standard inhibitor (IC_50_ = 750.0 ± 1.5 μM). Structurally, synthesized compounds **3a–ag** were divided into two main categories based on substituents on the benzimidazole moiety: unsubstituted benzimodazole derivatives **3a–x** and 7,8-dichlorosubsittuted benzimodazole derivatives **3y–ag**. To obtain an optimized *α*-glucosidase inhibitor, the substituents on 2-aryl and 4-aryl rings were changed in each category.

**Table 1:** Substrate scope and α-glucosidase inhibitory activity of compounds **3a–ag**.


Compound	**X**	**Ar**^**1**^	**Ar**^**2**^	**IC**_**50**_** (μM)**^b^	**IC**_**50**_** (μM)**^c^
3a	H			53.8 ± 0.04	225.3 ± 43.9
3b	H			37.8 ± 0.23	150.8 ± 2.4
3c	H			85.3 ± 0.36	341.2 ± 44.2
3d	H			26.7 ± 0.28	72.9 ± 11.7
3e	H			297.0 ± 1.2	> 600
3f.	H			193.8 ± 0.54	594.7 ± 46.8
3g	H			91.3 ± 0.4	323.2 ± 42.3
3h	H			42.6 ± 0.15	245.3 ± 50.7
3i	H			36.7 ± 0.01	180.2 ± 18.5
3j	H			114.3 ± 0.1	256.1 ± 21.2
3k	H			16.4 ± 0.36	45.0 ± 8.2
3l	H			28.0 ± 0.26	114.8 ± 13.5
3m	H			62.7 ± 0.36	332.4 ± 42.3
3n	H			48.4 ± 0.39	205.9 ± 27.4
3o	H			75.4 ± 0.42	325.8 ± 35.8
3p	H			65.4 ± 0.03	205.3 ± 37.6
3q	H			122.7 ± 0.6	404.1 ± 38.7
3r	H			128.4 ± 0.2	353.2 ± 3.9
3s	H			160.0 ± 0.36	495.3 ± 5.9
3t	H			188.5 ± 0.06	570.2 ± 70.1
3u	H			222.8 ± 0.15	> 600
3w	H			136.0 ± 0.08	538.9 ± 3.9
3x	H			254.7 ± 0.15	> 600
3y	Cl			78.4 ± 0.06	274.3 ± 14.9
3z	Cl			123.6 ± 0.26	461.5 ± 39.1
3aa	Cl			64.5 ± 0.15	256.1 ± 21.2
3ab	Cl			141.0 ± 1.1	431.3 ± 44.2
3ac	Cl			224.2 ± 0.15	> 600
3ad	Cl			48.4 ± 0.39	205.9 ± 27.4
3ae	Cl			72.9 ± 0.15	292.9 ± 14.9
3af	Cl			85.4 ± 0.04	341.3 ± 44.2
3ag	Cl			102.6 ± 0.23	412.5 ± 18.4
Acarbose	**–**	**–**	**–**	750.0 ± 1.5	145.7 ± 9.2

Values are the means of three replicates ± standard deviation (SD).

^b^The activity against Saccharomyces cerevisiae α-glucosidase.

^c^The activity against rat small intestine α-glucosidase.

Considering the substituents on 4-aryl ring, compounds **3a–x** were classified into five subcategories: (1) unsubstituted derivatives **3a–g**, (2) 4-chlorophenyl derivatives **3h–l**, (3) 4-bromophenyl derivatives **3m,n**, (4) 4-methoxyphenyl derivatives **3o–r**, (5) thiophene derivatives **3s**–**x**.

Among 3-amino-2,4-diarylbenzo[4,5]imidazo[1,2-*a*]pyrimidines **3a–g**, compound **3d** was found to be the most potent *α*-glucosidase inhibitor (IC_50_ = 26.7 ± 0.28 μM). Removing chlorine (compound **3a**) or replacing this atom with methyl or methoxy groups (compounds **3b** and **3c**, respectively) caused to decrease in inhibitory activity. Moreover, moving chlorine from 4-position to 2- and 3-position (compounds **3e** and **3f**, respectively) resulted into a considerable deterioration in activity, as compound **3e** had the least activity among all of the synthesized compounds (IC_50_ = 297.0 ± 1.2 μM). Additionally, compound **3g** bearing thiophene as 2-aryl ring showed lower activity (IC_50_ = 91.3 ± 0.4 μM) than compound **3d** (IC_50_ = 26.7 ± 0.28 μM).

Among 3-amino-2,4-diarylbenzo[4,5]imidazo[1,2-*a*]pyrimidines **3h–l**, compound **3k** with 4-Cl substituent on the both 2- and 4-phenyl rings exhibited the remarkable potency against *α*-glucosidase (IC_50_ = 16.4 ± 0.36 μM). It is worth noticing this derivative was 45.7 times more potent than the standard inhibitor (IC_50_ = 750.0 ± 1.5 μM), and it showed the highest inhibitory activity among all the synthesized compounds. Compound **3l** with 2-thiophene ring was the second most potent in this series (IC_50_ = 28.0 ± 0.26 μM). There was the same trend for the activities of compounds **3h–j** with their analogs in the first series (compounds **3a–c**). Additionally, results revealed that replacing chlorine at 4-position on 4-aryl ring of compounds **3h** and **3i** with bromine (compounds **3m** and **3n**) moderately decreased the *α*-glucosidase inhibitory activity.

In the fourth subcategory, compound **3o** with 2-phenyl exhibited relatively good inhibitory activity against *α*-glucosidase (IC_50_ = 75.4 ± 0.42 μM). Methylation on 4-position of this ring improved the activity (compound **3p** with IC_50_ = 65.4 ± 0.03 μM); however, introducing 4-OCH_3_ substituent (compound **3q**) or replacing this ring with thiophene (compound **3r**) led to decrease in its activity (IC_50_ = 122.7 ± 0.6 and 128.4 ± 0.2 μM, respectively).

Among derivatives **3s–x**, compound **3w** with 4-Cl on 2-phenyl ring was the most potent *α*-glucosidase inhibitor (IC_50_ = 136.0 ± 0.08 μM). By comparing the IC_50_ values of 4-methoxyphenyl derivatives **3o**–**r** and 4-thiophene derivatives **3s–x** with their analogs in previous series (compounds **3a–n**), it can be implied that methoxy and thiophene substituents caused significant deterioration on the *α*-glucosidase inhibitory activity. With this in mind, 3-amino-7,8-dichloro-2,4-diarylbenzo[4,5]imidazo[1,2-*a*]pyrimidines **3y–ag** bearing 4-phenyl (compounds **3y** and **3z**), 4-chlorophenyl derivatives (compounds **3aa–ae**), and 4-bromophenyl derivatives (compounds **3af** and **3ag**) were prepared to investigate their inhibitory activities.

Unsubstituted phenyl ring compound **3y** had good activity in comparison with other compounds (IC_50_ = 78.4 ± 0.06 µM). Introduction of a chlorine atom on the 4-position of the 2-phenyl ring, as in compound **3z** caused weaker activity (IC_50_ = 123.6 ± 0.26 µM). However, introducing this atom on the 4-position of the 4-phenyl ring (compound **3aa**) improved inhibitory activity (IC_50_ = 64.4 ± 0.15 µM). Adding electron-donating substituents including methyl and methoxy on the 4-position of the 2-phenyl ring (compounds **3ab** and **3ac**, respectively) caused significant decrease in inhibitory activity. Introducing another chlorine (compound **3ad**) improved the activity remarkably (IC_50_ = 48.4 ± 0.39 µM), as it has become the most potent inhibitor in the second category. Moreover, replacing chlorine atom in compound **3aa** activity (IC_50_ = 64.4 ± 0.15 µM) with bromine atom (compound **3af**) resulted in increased potency (IC_50_ = 85.4 ± 0.04 µM). Finally, compound **3ae** bearing thiophene as 2-aryl ring (IC_50_ = 72.9 ± 0.15 µM) showed slightly higher inhibitory comparing with compound **3aa** (IC_50_ = 64.4 ± 0.15 µM).

According to results, among derivatives in the first category (compounds **3a–x**), it seems the presence of 4-Cl on 2-aryl ring plays a substantial role in anti-*α*-glucosidase activities. The presence of electron-donating group (OCH_3_) on the 4-postion of 2- and 4-phenyl ring caused decrease in activity among all synthesized products. Additionally, the comparison of IC_50_ values of compounds **3a–x** with their corresponding 7,8-dichlorosubsittuted derivatives **3y–ag** revealed that the presence of chlorine atoms has deteriorate effect on the inhibitory activity of poly substituted benzo[4,5]imidazo[1,2-*a*]pyrimidines.

Additionally, the probable role of amine functional group has been investigated. For this goal, the *α*-glucosidase potency of compound **3a** (IC_50_ = 53.8 ± 0.04 μM) was compared with those of compounds **4a** and **6a** (the IC_50_ values were 235.4 ± 0.5 μM and 168.6 ± 1.2 μM, respectively). As it can be observed, the order of activity was NH_2_ > H > Cl substituted derivatives. Therefore, the necessary, constructive role of amine moiety on the inhibition of *α*-glucosidase has been confirmed.

To develop this investigation, the ability of our target compounds to inhibit the rat small intestine *α*-glucosidase have been evaluated. These inhibitory activities exhibited almost similar trend to that of *Saccharomyces cerevisiae α*-glucosidase. The most active compound was **3k** (IC_50_ value of 45.04 μM) which was 3.23 times more potent than acarbose (IC_50_ value of 145.74 μM). Moreover, compounds **4a** and **6a** showed slight inhibitory activities confirmed the significance of amine moiety in targeted compounds **3**.

### Enzyme kinetic study

**Figure 2:**
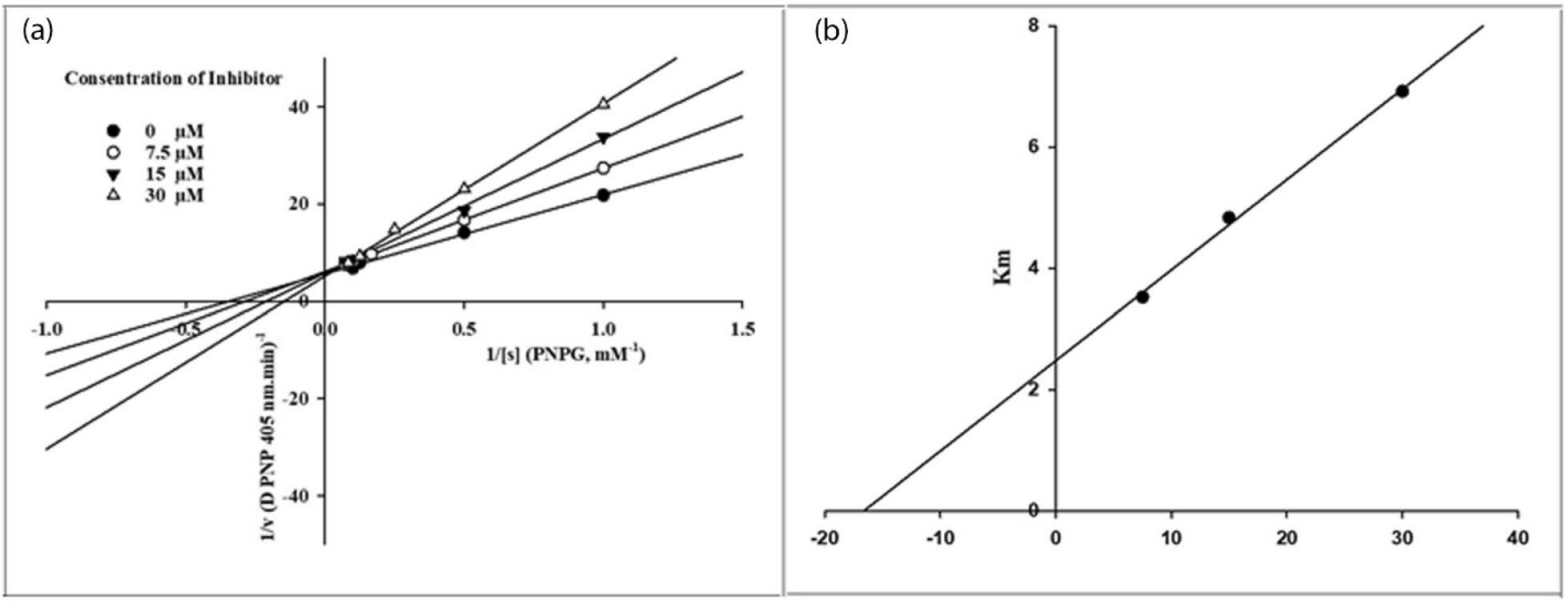
Kinetics of α-glucosidase inhibition by sample **3k**. (**a**) The Lineweaver–Burk plot in the absence and presence of different concentrations of sample **3k**; (**b**) The secondary plot between *K*_m_ and various concentrations of sample **3k**. *This figure was created by Microsoft Excel 2016 (https://www.microsoft.com/en-us/download/office.aspx).

To investigate the inhibition mode of synthesized poly-substituted 3-amino-2,4-diarylbenzo[4,5]imidazo[1,2-*a*]pyrimidine **3** against *α*-glucosidase, kinetic study was performed with standard inhibitor, acarbose, and the most potent derivative **3k**. To indicate the type of inhibition and K_i_, Lineweaver–Burk plots and secondary re-plotting of the mentioned plots were presented (Fig. 2). As it was showed in Fig. 2a, while inhibitor concentration increased, the K_m_ value gradually increased, but V_m_ value remained unchanged. Therefore, it can be implied compound **3k** was a competitive inhibitor and competes with acarbose for binding to the enzyme active site. Moreover, plot of K_m_ versus different concentration of compound **3k** gave an estimate of the inhibition constant, K_i_ of 16 µM (Fig. 2b).

### Cytotoxicity studies

Among the potent synthesized 3-amino-2,4-diarylbenzo[4,5]imidazo[1,2-*a*]pyrimidine **3**, the cytotoxicity of some of them including **3a**, **3k**, **3m** and **3ad** was evaluated through use of the breast cancer cell lines including MCF-7 and MDA-MB-231, as well as human pancreatic cancer cell lines including HDF and PANC1. The selected compounds did not possess any cytotoxic activity against these cell lines at concentration of 100 µM (IC_50_ > 200 µM).

### Docking study

**Figure 3:**
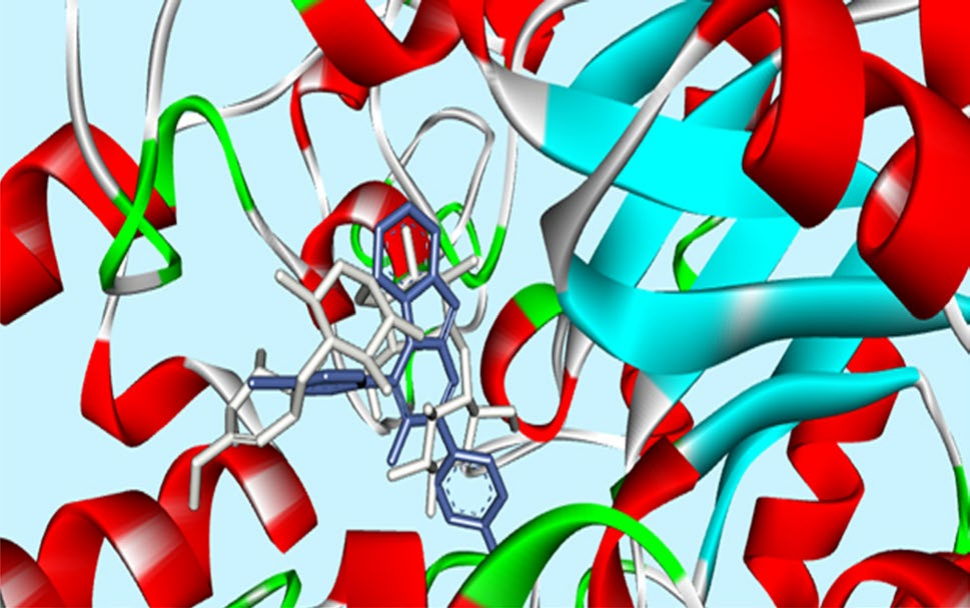
Acarbose (gray) and most potent compound **3k** (blue) superimposed in the active site pocket. *This figure was created by using Discovery Studio 4.0 Client (https://discover.3ds.com/discovery-studio-visualizer-download) and *LigPlot *(https://www.ebi.ac.uk/thornton-srv/software/LigPlus/download.html).

**Figure 4:**
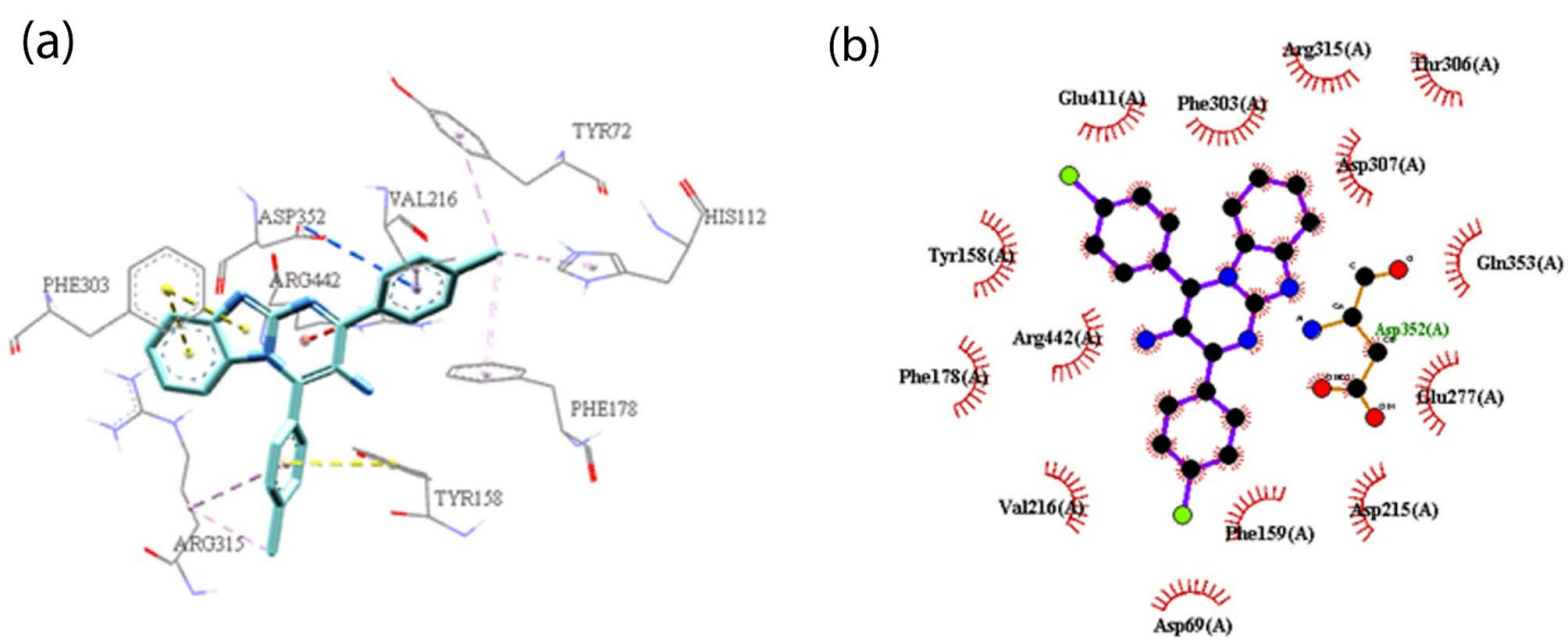
(**a**) The 3D and (**b**) 2D predicted binding mode of the compound **3k** in the active site pocket (π–π: yellow, π-Anion: blue, π-cation: red, hydrophobic: pink). *This figure was created by using Discovery Studio 4.0 Client (https://discover.3ds.com/discovery-studio-visualizer-download) and *LigPlot *(https://www.ebi.ac.uk/thornton-srv/software/LigPlus/download.html).

Molecular docking study was performed on the compounds **3a**, **3k** and **3ad** to study the mode of their interaction in the active site of the yeast isomaltase from *Saccharomyces cerevisiae* (Pdb id:3A4A) with 84% similarity to *S. cerevisiae* α-glucosidase using Auto Dock Tools (version 1.5.6). These compounds showed similar binding modes of interaction with catalytic residues. The superimposed structure of acarbose as a standard inhibitor and the most potent compound **3k** in the active site of isomaltase was shown in Fig. 3. In the most potent compound **3k,** benzimidazole and 4-(4Cl-phenyl) ring units created π–π interaction with Phe 303 and Tyr 158, respectively in the active site of the enzyme (Fig. 4). The 2-(4Cl-phenyl) ring formed π-anion interaction with the aromatic side chains of Asp352. Moreover, a π-cation interaction was observed between pyrimidine moiety and Arg 442.

**Figure 5:**
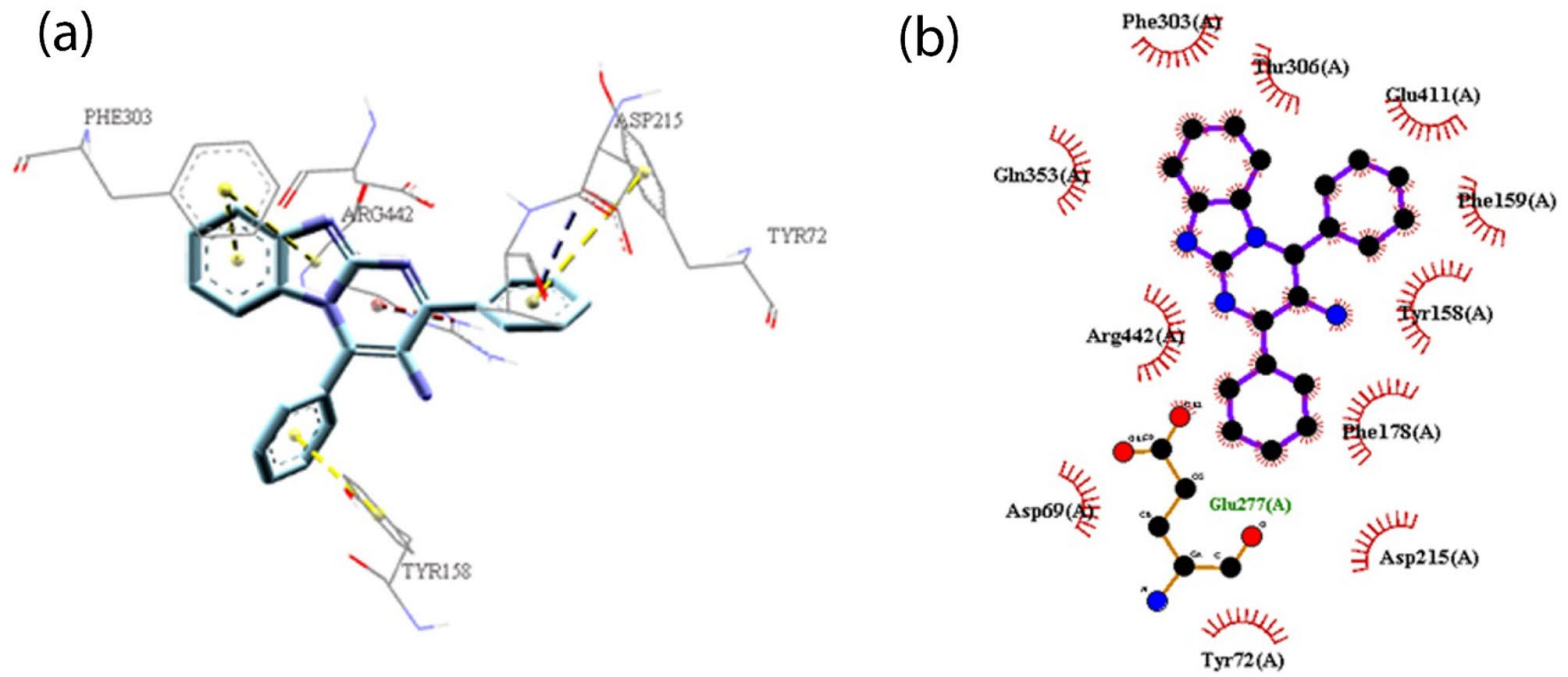
(**a**) The 3D and (**b**) 2D predicted binding mode of the compound **3a** in the active site pocket (π–π: yellow, π-Anion: blue, π-cation: red, hydrophobic: pink). *This figure was created by using Discovery Studio 4.0 Client (https://discover.3ds.com/discovery-studio-visualizer-download) and *LigPlot *(https://www.ebi.ac.uk/thornton-srv/software/LigPlus/download.html).

Compounds **3a** and **3k** interacted with similar amino acids in the active site of the enzyme. Benzimidazol, pyrimidine, and 4-phenyl ring of compound **3a** interacted with Phe303, Arg442, and Tyr158, respectively (Fig. 5). Compound **3k** had additional π-alkyl interaction between 4-(4Cl-phenyl) ring and Arg315, as well as 2-(4Cl-phenyl) ring and Val 216. Higher observed inhibitory activity of compound **3k** could be attributed to the formation of stabilizing interactions with specific residues like Arg315 and Val 216, which could be resulted from the presence of chlorine atoms led to the electron-deficiency of phenyl rings. Additionally, chlorine atoms in compound **3k** could create hydrophobic interactions with Tyr72, His112, Phe178, Arg315 which brought more inhibitory activity in comparison with compound **3a**.

**Figure 6:**
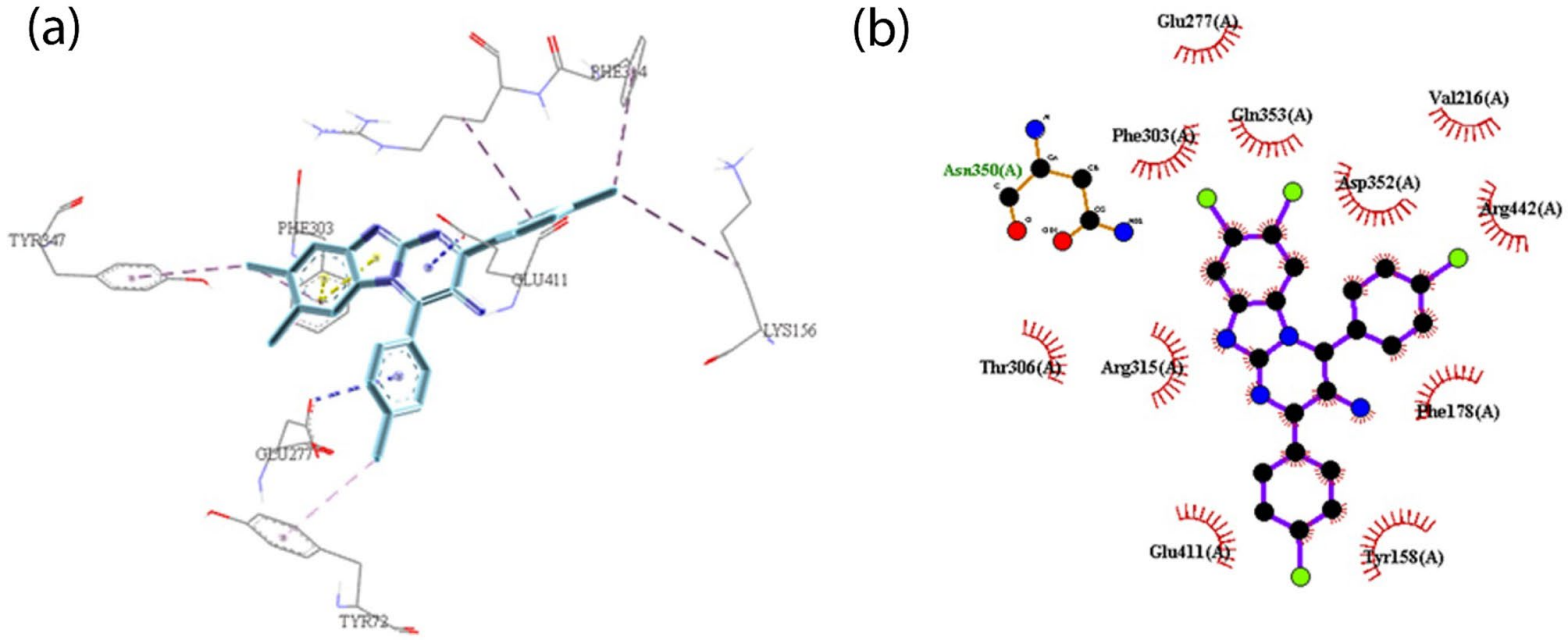
(**a**) The 3D and (**b**) 2D predicted binding mode of the compound **3ad** in the active site pocket (π–π: yellow, π-Anion: blue, π-cation: red, hydrophobic: pink). *This figure was created by using Discovery Studio 4.0 Client (https://discover.3ds.com/discovery-studio-visualizer-download) and *LigPlot *(https://www.ebi.ac.uk/thornton-srv/software/LigPlus/download.html).

In compound **3ad**, there was a difference in interaction mode of the 2-(4Cl-phenyl) moiety with the active site of enzyme. Insertion of chlorine in 7 and 8 positions on the benzimidazole moiety led to a significant decrease in the inhibitory activity. However, there was not any interaction between 2-(4Cl-phenyl) moiety and Asp352 (Fig. 6).

Further studies on the binding energies of selected compounds exhibited that compound **3k** had lower free binding energy (− 9.63 kcal/mol) as compared to compounds **3ad** (− 8.89 kcal/mol) and **3a** (− 9.14 kcal/mol). As observed from the best docking conformations, showed that all three compounds have a lower free binding energy than acarbose (− 8.20 kcal/mol). Therefore, the results emphasized that the target compounds bind more easily to the target enzyme (*α*-glucosidase) than the reference drug, acarbose. These findings had good agreement with the obtained results through in vitro experiments.

**Figure 7:**
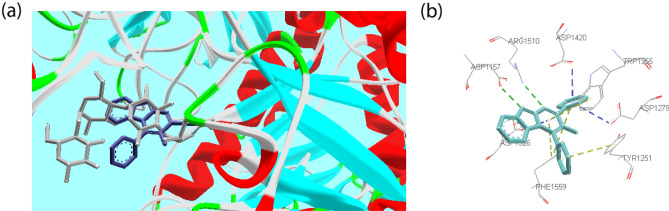
(**a**) Acarbose (gray) and compound **3a** (blue) superimposed in the human intestinal α-glucosidase active site. (**b**) 3D predicted binding mode of the compound **3a** in the human intestinal α-glucosidase active site (H-bond: green, π–π: yellow, π-Anion: blue). *This figure was created by using Discovery Studio 4.0 Client (https://discover.3ds.com/discovery-studio-visualizer-download) and *LigPlot *(https://www.ebi.ac.uk/thornton-srv/software/LigPlus/download.html).

To assess potential inhibition of human α-glucosidase, compound **3a** was docked against the crystal structure for *C*-terminal domain of human intestinal *α*-glucosidase (PDB Code: 3TOP) comparing with Acarbose. This study exhibited similar interactions with the yeast isomaltase binding site. The superimposed structure of acarbose and compound **3a** in the active site of human intestinal α-glucosidase was shown in Fig. 7. Interestingly, compound **3a** exhibited better binding energy (− 10.47 kcal/mol) than Acarbose (− 8.85 kcal/mol). Benzimidazole moiety in this compound created hydrogen bond interaction with Asp 1157 and π-anion interaction with Asp 1526. Another important hydrogen bond interaction was observed between pyrimidine moiety and Arg 1510. Phenyl rings and pyrimidine were involved in several π-anion interactions with Asp 1279, Asp 1420, and Asp 1526. Moreover, compound **3a** formed π–π stacking interaction with hydrophobic residue including Tyr1251, Trp1355, and Phe1559.

## Conclusion

In conclusion, we introduced a novel, potent series of *α*-glucosidase inhibitors. Poly-substituted 3-amino-2,4-diarylbenzo[4,5]imidazo[1,2-*a*]pyrimidines were synthesized through an efficient, short-time, high-yield Michael addition–cyclization between 2-aminobenzimidazoles and *α*-azidochalcones under the mild conditions. No need to column chromatography led us to obtain a large scope of substrates, all of which exhibited good to excellent inhibitory activity. Among them, compound **3k** showed the best inhibitory potency having IC_50_ value of 16.4 ± 0.36 μM which was 45.7 times more potent than acarbose as standard inhibitor (IC_50_ = 750.0 ± 1.5 μM). The kinetic study for this compound showed there was a competitive mechanism. Moreover, docking studies revealed that 3-amino-2,4-diarylbenzo[4,5]imidazo[1,2-*a*]pyrimidines could interact with important amino acids in the active site of *α*-glucosidase.

## Experimental

### Methods

All chemicals were purchased from Merck (Germany) and were used without further purification. Melting points were measured on an Electrothermal 9100 apparatus and were not corrected. Mass spectra were recorded on an Agilent Technologies (HP) 5973 mass spectrometer operating at an ionization potential of 20 eV. IR spectra were recorded on a Shimadzu IR-460 spectrometer. ^1^H and ^13^C NMR spectra were measured (DMSO‑*d*_6_ solution) with Bruker DRX-300 AVANCE (at 300.1 and 75.1 MHz) spectrometer with TMS as an internal standard. *α*-Azido chalcones **2** were obtained from the corresponding benzylidene acetophenones in two steps following the literature procedure^15^.

### General procedure for the preparation of 3-amino-2,4-diarylbenzo[4,5]imidazo[1,2-*a*]pyrimidines 3a–ag

A solution of 2-aminobenzimidazoles **1** (1.2 mmol), *α*-azidochalcones **2** (1.0 mmol), Et_3_N (1.2 mmol) in EtOH (5.0 mL) was magnetically stirred for 2 h under reflux conditions. After completion of the reaction according to the TLC analysis, the mixture was cooled to ambient temperature, the precipitated product was filtered and washed with Et_2_O (5.0 mL) to afford pure products as yellow powder.

### General procedure for the preparation of 3-chloro-2,4-diphenylbenzo[4,5]imidazo[1,2-*a*]pyrimidine 4a

To a stirring solution of concentrated sulfuric acid (1.6 mmol), sodium nitrite (2.2 mmol) was added gradually over 10–15 min. After addition was completed, the temperature was raised to 70 °C, and the mixture was stirred until sodium nitrite dissolved thoroughly. Then, the mixture is cooled to 25 °C with an ice bath, and a solution of 3-amino-2,4-diphenylbenzo[4,5]imidazo[1,2-a]pyrimidin **3a** (2.0 mmol) in glacial acetic acid (4.0 ml) was added slowly with stirring, at such a rate that temperature remains below 40 °C. After 30 min, TLC monitoring confirmed compound **3a** was completely converted to corresponding diazonium salt. The obtained mixture was added at 10 °C in portions to a solution of CuCl (4.4 mmol) in concentrated hydrochloric acid (4.0 mmol) over a period of about 5 min. Afterward, temperature was raised to 80 °C and the reaction mixture was heated for almost 5 h. After completion of the reaction which was monitored by TLC, mixture was quenched by iced water. The precipitate was filtered and recrystallized in EtOH to afford the pure product **4a**.

### General procedure for the preparation of 2,4-diphenylbenzo[4,5]imidazo[1,2-*a*]pyrimidine 6a

A mixture of 2-aminobenzimidazole **1a** (1.2 mmol), chalcone **5a** (1.0 mmol), and Et_3_N (1.2 mmol) in EtOH (5.0 mL) was heated under reflux conditions for 10 h. After completion of the reaction confirmed by the TLC analysis, the solvent was removed under the reduced pressure. The residue was purified by column chromatography using n-hexane/EtOAc (3:1) as eluent to afford pure product **6a**.

### 2,4-Diphenyl-benzo[4,5]imidazo[1,2-a]pyrimidin-3-ylamine (3a)

Yellow solid; yield: 89%, mp 208–210 °C. IR (KBr) (*ν*_max_/cm^–1^): 3459 and 3372 (NH_2_), 1594, 1426, 1378, 1302, 1228, 1194, 1148, 1083, 1016, 984, 906, 801, 746, 668, 624. ^1^H NMR (300.1 MHz, DMSO): *δ* 8.15 (d, *J* = 8.4 Hz, 2H, 2CH), 8.10–7.20 (m, 9H, 9CH), 7.11 (t, *J* = 7.5 Hz, 1H, CH), 6.87 (t, *J* = 7.4 Hz, 1H, CH), 6.18 (d, *J* = 8.2 Hz, 1H, CH), 4.13 (s, 2H, NH_2_). ^13^C NMR (75.5 MHz, DMSO-*d*_6_): *δ* 155.2, 148.2, 144.9, 136.8, 136.4, and 130.8 (6C), 130.4 and 130.3 (2CH), 129.9 (2CH), 129.7 (2CH), 129.2 (2CH), 128.6 (CH), 127.9 (2CH), 127.4 and 126.4 (2C), 124.9, 120.0 and 119.2 (3CH). EI-MS, *m/z* (%): 336 (M^+^, 27), 133 (100), 105 (80), 79 (43), 52 (35).

### 2-Phenyl-4-p-tolyl-benzo[4,5]imidazo[1,2-a]pyrimidin-3-ylamine (3b)

Yellow solid; yield: 86%, mp 272–274 °C. IR (KBr) (*ν*_max_/cm^–1^): 3428 and 3362 (NH_2_), 1595, 1434, 1399, 1356, 1256, 1181, 1085, 1014, 991, 829, 754, 685, 650. ^1^H NMR (300.1 MHz, DMSO): *δ* 8.17 (d, *J* = 8.3 Hz, 2H, 2CH), 7.76 (d, *J* = 7.8 Hz, 1H, CH), 7.60–7.24 (m, 7H, 7CH), 7.10 (t, *J* = 7.5 Hz, 1H, CH), 6.91 (t, *J* = 7.4 Hz, 1H, CH), 6.28 (d, *J* = 8.2 Hz, 1H, CH), 4.04 (s, 2H, NH_2_), 2.36 (s, 3H, CH_3_). ^13^C NMR (75.5 MHz, DMSO-*d*_6_): *δ* 155.3, 148.2, 144.9, 137.5, 136.9 and 136.4 (6C), 130.9 (2CH), 130.6 (CH), 129.7 (2 × 2CH), 129.2 (CH), 128.6 (C), 127.9 (2CH), 127.4 and 126.8 (2C), 124.8, 119.9 and 119.1 (3CH), 21.1 (CH_3_). EI-MS, *m/z* (%): 351 (M^+^  + 1, 100), 332 (18), 273 (72), 257 (15), 169 (48), 91 (10), 76 (25).

### 4-(4-Methoxy-phenyl)-2-phenyl-benzo[4,5]imidazo[1,2-a]pyrimidin-3-ylamine (3c)

Yellow solid; yield: 74%, mp 267–271 °C. IR (KBr) (*ν*_max_/cm^–1^): 3438 and 3389 (NH_2_), 1592, 1414, 1367, 1283, 1182, 1144, 1068, 991, 947, 843, 805, 753, 675, 623. ^1^H NMR (300.1 MHz, DMSO):* δ* 7.91 (d, *J* = 8.4 Hz, 2H, 2CH), 7.74 (d, *J* = 8.5 Hz, 1H, CH), 7.63–7.52 (m, 5H, 5CH), 7.37–7.27 (m, 3H, 3CH), 6.94 (t, *J* = 7.4 Hz, 1H, CH), 6.29 (d, *J* = 8.4 Hz, 1H, CH), 4.03 (s, 2H, NH_2_), 3.91 (s, 3H, OCH_3_). ^13^C NMR (75.5 MHz, DMSO-*d*_6_): *δ* 160.9, 156.8, 148.1, 144.5 and 137.1 (5C), 131.2 (2CH), 130.2 and 129.9 (2CH), 129.7(C), 128.6 (2 × 2CH), 127.7, 127.2 and 126.6 (3C), 124.6, 119.9 and 119.1 (3CH), 115.7 (2CH), 55.4 (OCH_3_). EI-MS, *m/z* (%): 366 (M^+^, 100), 288 (45), 244 (15), 202 (43), 185 (29), 76 (14), 51 (32).

### 4-(4-Chloro-phenyl)-2-phenyl-benzo[4,5]imidazo[1,2-a]pyrimidin-3-ylamine (3d)

Yellow solid; yield: 90%, mp 252–253 °C. IR (KBr) (*ν*_max_/cm^–1^): 3448 and 3363 (NH_2_), 1598, 1458, 1397, 1368, 1287, 1158, 1079, 1012, 994, 935, 897, 752, 689, 623. ^1^H NMR (300.1 MHz, DMSO):* δ* 7.95–7.63 (m, 7H, 7CH), 7.60–7.47 (m, 3H, 3CH), 7.33 (t, *J* = 7.5 Hz, 1H, CH), 6.96 (t, *J* = 7.4 Hz, 1H, CH), 6.25 (d, *J* = 8.4 Hz, 1H, CH), 4.20 (s, 2H, NH_2_). ^13^C NMR (75.5 MHz, DMSO-*d*_6_): *δ* 155.4, 148.0, 144.5, 138.8, 136.9 and 135.6 (6C), 132.0 (2CH), 131.5 (CH), 130.5 (2CH), 129.9 (CH), 128.6 (2 × 2CH), 128.1, 127.0 and 126.6 (3C), 124.6, 120.1 and 119.2 (3CH). EI-MS, *m/z* (%): 372 (M^+^ + 2, 48), 293 (34), 278 (100), 258 (28), 204 (10), 111 (23).

### 4-(2-Chloro-phenyl)-2-phenyl-benzo[4,5]imidazo[1,2-a]pyrimidin-3-ylamine (3e)

Yellow solid; yield: 68%, mp 276–277 °C. IR (KBr) (*ν*_max_/cm^–1^): 3436 and 3385 (NH_2_), 1593, 1484, 1389, 1345, 1278, 1169, 1149, 1079, 991, 936, 899, 842, 799, 753, 685, 655. ^1^H NMR (300.1 MHz, DMSO):* δ* 8.04–7.71 (m, 6H, 6CH), 7.67 (d, *J* = 7.6 Hz, 1H, CH), 7.63–7.47 (m, 3H, 3CH), 7.32 (t, *J* = 7.5 Hz, 1H, CH), 6.95 (t, *J* = 7.8 Hz, 1H, CH), 6.19 (d, *J* = 7.8 Hz, 1H, CH), 4.21 (s, 2H, NH_2_). ^13^C NMR (75.5 MHz, DMSO-*d*_6_): *δ* 157.1, 147.9, 144.5, 136.9, 134.8 and 132.2 (6C), 131.8, 130.9, 129.9, 129.8 and 128.72 (5CH), 128.66 (2CH), 128.60 (2CH), 127.8, 127.0 and 126.6 (3C), 124.6, 120.1, 119.2 and 113.5 (4CH). EI-MS, *m/z* (%): 370 (M^+^, 100), 334 (18), 294 (34), 189 (28), 204 (10), 111 (23).

### 4-(3-Chloro-phenyl)-2-phenyl-benzo[4,5]imidazo[1,2-a]pyrimidin-3-ylamine (3f)

Yellow solid; yield: 73%, mp 268–269 °C. IR (KBr) (*ν*_max_/cm^–1^): 3443 and 3359 (NH_2_), 1596, 1501, 1346, 1277, 1182, 1149, 1084, 993, 825, 785, 685, 635. ^1^H NMR (300.1 MHz, DMSO):* δ* 7.96–7.85 (m, 3H, 3CH), 7.83–7.67 (m, 4H, 4CH), 7.63–7.50 (m, 3H, 3CH), 7.35 (t, *J* = 7.7 Hz, 1H, CH), 6.96 (t, *J* = 7.6 Hz, 1H, CH), 6.13 (d, *J* = 8.4 Hz, 1H, CH), 4.29 (s, 2H, NH_2_). ^13^C NMR (75.5 MHz, DMSO-*d*_6_): *δ* 157.0, 147.7, 144.4, 136.8 and 133.8 (5C), 133.0, 132.4, 130.7, 130.0 and 129.2 (5CH), 128.7 (2CH), 128.6 (2CH), 128.5, 126.92, 126.85 and 126.1 (4C), 124.7, 120.6, 119.3 and 112.6 (4CH). EI-MS, *m/z* (%): 370 (M^+^, 78), 320 (25), 293 (34), 244 (100), 182 (48), 109 (23), 77 (48).

### 2-Phenyl-4-thiophen-2-yl-benzo[4,5]imidazo[1,2-a]pyrimidin-3-ylamine (3g)

Yellow solid; yield: 69%, mp 246–248 °C. IR (KBr) (*ν*_max_/cm^–1^): 3458 and 3376 (NH_2_), 1595, 1512, 1397, 1282, 1178, 1132, 1075, 989, 923, 824, 732, 684, 652. ^1^H NMR (300.1 MHz, DMSO-*d*_6_): *δ* 8.19 (d, *J* = 3.5 Hz, 1H, CH), 7.89 (d, *J* = 5.0 Hz, 1H, CH), 7.83–7.48 (m, 6H, 6CH), 7.36–7.21 (m, 2H, 2CH), 6.95 (t, *J* = 7.4 Hz, 1H, CH), 6.26 (d, *J* = 8.3 Hz, 1H, CH), 4.25 (s, 2H, NH_2_). ^13^C NMR (75.5 MHz, DMSO-*d*_6_): *δ* 156.1, 150.4, 147.9, 144.6, 141.4 and 131.6 (6C), 131.3 and 130.4 (2CH), 129.9 (2CH), 128.7 (2CH), 128.0 (CH), 127.3 and 126.6 (2C), 125.7, 124.7, 120.2, 119.4 and 113.3 (5CH). EI-MS, *m/z* (%): 342 (M^+^, 100), 266 (25), 248 (78), 168 (43), 135 (36), 105 (28), 77 (48), 51 (25).

### 2-(4-Chloro-phenyl)-4-phenyl-benzo[4,5]imidazo[1,2-a]pyrimidin-3-ylamine (3h)

Yellow solid; yield: 84%, mp 265–266 °C. IR (KBr) (*ν*_max_/cm^–1^): 3468 and 3373 (NH_2_), 1607, 1498, 1424, 1358, 1284, 1203, 1163, 1041, 991, 953, 885, 743, 696, 641. ^1^H NMR (300.1 MHz, DMSO-*d*_6_): *δ* 7.96 (d, *J* = 8.4 Hz, 2H, 2CH), 7.85–7.52 (m, 8H, 8CH), 7.31 (t, *J* = 7.7 Hz, 1H, CH), 6.89 (t, *J* = 7.9 Hz, 1H, CH), 6.11 (d, *J* = 8.4 Hz, 1H, CH), 4.14 (s, 2H, NH_2_). ^13^C NMR (75.5 MHz, DMSO-*d*_6_): *δ* 155.8, 148.0, 144.6, 135.9, 134.7 and 134.1 (6C), 130.9 (CH), 130.7 (2CH), 130.4 (2CH), 130.1 (C), 129.71 (2CH), 129.66 (CH), 128.6 (2CH), 127.1 and 126.3 (2C), 124.7, 120.0 and 119.2 (3CH). EI-MS, *m/z* (%): 370 (M^+^, 100), 333 (18), 232 (25), 206 (36), 167 (29), 102 (45), 77 (51), 51 (25).

### 2-(4-Chloro-phenyl)-4-p-tolyl-benzo[4,5]imidazo[1,2-a]pyrimidin-3-ylamine (3i)

Yellow solid; yield: 78%, mp 277–278 °C. IR (KBr) (*ν*_max_/cm^–1^): 3468 and 3348 (NH_2_), 1602, 1487, 1412, 1368, 1294, 1235, 1132, 1098, 1032, 991, 928, 848, 776, 729, 687, 635. ^1^H NMR (300.1 MHz, DMSO-*d*_6_): *δ* 7.93 (d, *J* = 8.3 Hz, 2H, 2CH), 7.66–7.46 (m, 7H, 7CH), 7.31 (t, *J* = 7.9 Hz, 1H, CH), 6.90 (t, *J* = 7.6 Hz, 1H, CH), 6.22 (d, *J* = 8.5 Hz, 1H, CH), 4.09 (s, 2H, NH_2_), 2.43 (s, 3H, CH_3_). ^13^C NMR (75.5 MHz, DMSO-*d*_6_): *δ* 155.6, 148.0, 144.5, 135.9 and 134.6 (5C), 131.4 (CH), 130.9 (2CH), 130.6 (2CH), 130.2 and 129.8 (2C), 129.5 (2CH), 128.6 (2CH), 127.1, 126.6 and 126.4 (3C), 124.7, 120.0 and 119.1(3CH), 21.2 (CH_3_). EI-MS, *m/z* (%): 384 (M^+^, 100), 293 (46), 276 (32), 218 (68), 109 (18), 91 (22).

### 2-(4-Chloro-phenyl)-4-(4-methoxy-phenyl)-benzo[4,5]imidazo[1,2-a]pyrimidin-3-ylamine (3j)

Yellow solid; yield: 83%, mp 257–258 °C. IR (KBr) (*ν*_max_/cm^–1^): 3446 and 3372 (NH_2_), 1595, 1493, 1427, 1359, 1295, 1236, 1149, 1085, 1035, 972, 939, 858, 784, 740, 655, 637. ^1^H NMR (300.1 MHz, DMSO-*d*_6_): *δ* 8.18 (d, *J* = 8.6 Hz, 2H, 2CH), 7.95 (d, *J* = 8.4 Hz, 2H, 2CH), 7.74 (d, *J* = 8.0 Hz, 1H, CH), 7.30 (d, *J* = 8.4 Hz, 2H, 2CH), 7.09–7.05 (m, 1H, CH), 7.03 (d, *J* = 8.5 Hz, 2H, 2CH), 6.94 (t, *J* = 7.8 Hz, 1H, CH), 6.28 (d, *J* = 8.6 Hz, 1H, CH), 4.12 (s, 2H, NH_2_), 3.74 (s, 3H, OCH_3_). ^13^C NMR (75.5 MHz, DMSO-*d*_6_): *δ* 157.4, 155.6, 147.8, 145.0, 135.9 and 134.6 (6C), 131.3 (2CH), 131.2 (CH), 130.1 (C), 128.6 (2CH), 127.7 (2CH), 127.3, 127.1 and 126.7 (3C), 124.7, 120.0 and 119.1 (3CH), 115.8 (2CH), 55.4 (OCH_3_). EI-MS, *m/z* (%): 402 (M^+^ + 2, 100), 292 (25), 276 (48), 111 (10), 106 (56), 92 (28), 51 (32).

### 2,4-Bis-(4-chloro-phenyl)-benzo[4,5]imidazo[1,2-a]pyrimidin-3-ylamine (3k)

Yellow solid; yield: 92%, mp 271–272 °C. IR (KBr) (*ν*_max_/cm^–1^): 3447 and 3356 (NH_2_), 1599, 1506, 1436, 1348, 1294, 1233, 1172, 1061, 990, 898, 831, 786, 686, 635. ^1^H NMR (300.1 MHz, DMSO-*d*_6_): *δ* 7.93 (d, *J* = 8.5 Hz, 2H, 2CH), 7.82 (d, *J* = 8.4 Hz, 2H, 2CH), 7.75 (d, *J* = 8.3 Hz, 1H, CH), 7.72 (d, *J* = 8.5 Hz, 2H, 2CH), 7.62 (d, *J* = 8.4 Hz, 2H, 2CH), 7.33 (t, *J* = 7.8 Hz, 1H, CH), 6.96 (t, *J* = 7.8 Hz, 1H, CH), 6.24 (d, *J* = 8.4 Hz, 1H, CH), 4.25 (s, 2H, NH_2_). ^13^C NMR (75.5 MHz, DMSO-*d*_6_): *δ* 155.8, 147.9, 144.5, 135.8, 135.6 and 134.7 (6C), 131.9 (2CH), 131.4 (CH), 130.6 (2CH), 130.5 (2CH), 128.6 (2CH), 128.51, 128.48, 127.0 and 126.7 (4C), 124.7, 120.2 and 119.2 (3CH). EI-MS, *m/z* (%): 404 (M^+^, 100), 333 (45), 293 (65), 270 (15), 258 (34), 166 (18), 103 (28), 77 (38), 52 (26).

### 3-(4-Chloro-phenyl)-1-thiophen-2-yl-benzo[4,5]imidazo[1,2-a]pyridin-2-ylamine (3l)

Yellow solid; yield: 71%, mp 246–245 °C. IR (KBr) (*ν*_max_/cm^–1^): 3459 and 3374 (NH_2_), 1586, 1502, 1435, 1370, 1283, 1256, 1082, 1046, 932, 845, 760, 638. ^1^H NMR (300.1 MHz, DMSO-*d*_6_): *δ* 8.23 (d, *J* = 3.6 Hz, 1H, CH), 8.02 (d, *J* = 8.4 Hz, 2H, 2CH), 7.90 (d, *J* = 4.8 Hz, 1H, CH), 7.75 (d, *J* = 8.3 Hz, 1H, CH), 7.40 (d, *J* = 8.4 Hz, 2H, 2CH), 7.32–7.21 (m, 2H, 2CH), 6.98 (t, *J* = 7.5 Hz, 1H, CH), 6.31 (d, *J* = 8.4 Hz, 1H, 1CH), 4.15 (s, 2H, NH_2_). ^13^C NMR (75.5 MHz, DMSO-*d*_6_): *δ* 155.7, 151.0, 148.2, 144.8, 142.4 and 135.4 (6C), 131.1 (CH), 130.9 (2CH), 130.8 and 128.3 (2C), 128.2 (2CH), 127.4 (CH), 126.7 (C) 125.6, 124.7, 119.2, 118.9 and 114.0 (5CH). EI-MS, *m/z* (%): 376 (M^+^, 38), 265 (100), 294 (48), 209 (10), 184 (75), 167 (20), 128 (13), 99 (27).

### 2-(4-Bromo-phenyl)-4-phenyl-benzo[4,5]imidazo[1,2-a]pyrimidin-3-ylamine (3m)

Yellow solid; yield: 83%, mp 268–271 °C. IR (KBr) (*ν*_max_/cm^–1^): 3429 and 3384 (NH_2_), 1583, 1498, 1445, 1358, 1267, 1242, 1145, 1061, 995, 846, 789, 748, 674, 625. ^1^H NMR (300.1 MHz, DMSO-*d*_6_): *δ* 8.10 (d, *J* = 8.3 Hz, 2H, 2CH), 7.75 (d, *J* = 7.8 Hz, 1H, CH), 7.64–7.48 (m, 7H, 7CH), 7.33 (t, *J* = 7.7 Hz, 1H, CH), 6.85 (t, *J* = 7.4 Hz, 1H, CH), 6.17 (d, *J* = 8.4 Hz, 1H, CH), 4.15 (s, 2H, NH_2_). ^13^C NMR (75.5 MHz, DMSO-*d*_6_): *δ* 155.1, 147.9, 144.9, 136.3 and 136.0 (5C), 131.6 (2CH), 130.92 (C), 130.86 (2CH), 130.4 and 130.1 (2CH), 129.8 (2CH), 129.1 (2CH), 127.3 and 126.4 (2C), 124.8 (CH), 123.6 (C), 119.9 and 119.2 (2CH). EI-MS, *m/z* (%): 416 (M^+^, 100), 333 (17), 232 (15), 206 (22), 167 (35), 133 (16), 102 (33), 77 (38), 51 (13).

### 2-(4-Bromo-phenyl)-4-p-tolyl-benzo[4,5]imidazo[1,2-a]pyrimidin-3-ylamine (3n)

Yellow solid; yield: 90%, mp 278–279 °C. IR (KBr) (*ν*_max_/cm^–1^): 3469 and 3342 (NH_2_), 1605, 1492, 1428, 1371, 1295, 1245, 1180, 1045, 1015, 923, 879, 753, 695, 634. ^1^H NMR (300.1 MHz, DMSO-*d*_6_): *δ* 7.87 (d, *J* = 8.3 Hz, 2H, 2CH), 7.80–7.65 (m, 5H, 5CH), 7.64–7.48 (m, 4H, 4CH), 7.31 (t, *J* = 7.5 Hz, 1H, CH), 6.91 (t, *J* = 7.5 Hz, 1H, CH), 6.23 (d, *J* = 8.7 Hz, 1H, CH), 4.10 (s, 2H, NH_2_), 2.29 (s, 3H, CH_3_). ^13^C NMR (75.5 MHz, DMSO-*d*_6_): *δ* 155.7, 148.0, 144.6, 136.2, 135.5 (5C), 131.5 (2 × 2CH), 130.89 (2CH), 130.85 (2CH), 130.7 (CH), 129.5 (2CH), 129.2, 127.1, 126.6 and 126.4 (4C), 124.7 (CH), 123.5 (C), 120.0 and 119.1 (2CH), 21.2 (CH_3_). EI-MS, *m/z* (%): 428 (M^+^, 100), 338 (64), 273 (26), 184 (47), 172 (10), 156 (28), 107 (22).

### 2-(4-Methoxy-phenyl)-4-phenyl-benzo[4,5]imidazo[1,2-a]pyrimidin-3-ylamine (3o)

Yellow solid; yield: 65%, mp 248–250 °C. IR (KBr) (*ν*_max_/cm^–1^): 3473 and 3359 (NH_2_), 1604, 1458, 1388, 1292, 1239, 1198, 1132, 1043, 994, 926, 831, 756, 698, 624. ^1^H NMR (300.1 MHz, DMSO-*d*_6_): *δ* 7.82 (d, *J* = 8.5 Hz, 2H, 2CH), 7.70 (d, *J* = 8.0 Hz, 1H, CH), 7.60–7.42 (m, 5H, 5CH), 7.38 (t, *J* = 7.9 Hz, 1H, CH), 7.07 (d, *J* = 8.5 Hz, 2H, 2CH), 6.96 (t, *J* = 7.6 Hz, 1H, CH), 6.30 (d, *J* = 8.3 Hz, 2H, 2CH), 4.13 (s, 2H, NH_2_), 3.82 (s, 3H, OCH_3_). ^13^C NMR (75.5 MHz, DMSO-*d*_6_): *δ* 160.0, 158.7, 148.2, 145.3 and 136.3 (5C), 130.5 (2CH), 130.2 and 129.9 (2CH), 129.4 (C), 129.3 (2CH), 128.6 (2CH), 127.7, 127.3 and 126.5 (3C), 124.6, 120.6 and 119.2 (3CH), 114.2 (2CH), 55.0 (OCH_3_). EI-MS, *m/z* (%): 366 (M^+^, 100), 274 (58), 201 (23), 188 (26), 92 (46), 79 (10), 51 (45).

### 2-(4-Methoxy-phenyl)-4-p-tolyl-benzo[4,5]imidazo[1,2-a]pyrimidin-3-ylamine (3p)

Yellow solid; yield: 84%, mp 274–275 °C. IR (KBr) (*ν*_max_/cm^–1^): 3438 and 3329 (NH_2_), 1605, 1458, 1401, 1376, 1273, 1178, 1075, 1023, 983, 878, 768, 659, 621. ^1^H NMR (300.1 MHz, DMSO-*d*_6_): *δ* 7.92 (d, *J* = 8.4 Hz, 2H, 2CH), 7.72 (d, *J* = 7.8 Hz, 1H, CH), 7.56 (d, *J* = 8.3 Hz, 2H, 2CH), 7.53 (d, *J* = 8.3 Hz, 2H, 2CH), 7.30 (t, *J* = 7.5 Hz, 1H, CH), 7.09 (d, *J* = 8.6 Hz, 2H, 2CH), 7.01 (t, *J* = 7.8 Hz, 1H, CH), 6.90 (d, *J* = 7.7 Hz, 1H, CH), 4.03 (s, 2H, NH_2_), 3.83 (s, 3H, OCH_3_), 2.39 (s, 3H, CH_3_). ^13^C NMR (75.5 MHz, DMSO-*d*_6_): *δ* 156.5, 155.2, 147.5, 144.5 and 137.7 (5C), 131.5 (CH), 130.9 (2CH), 129.9 (2CH), 129.5 (2CH), 129.3, 128.8, 127.1, 126.8 and 126.4 (5C), 124.5, 119.8 and 119.1 (3CH), 111.5 (2CH), 55.3 (OCH_3_), 21.2 (CH_3_). EI-MS, *m/z* (%): 380 (M^+^, 43), 278 (66), 167 (100), 135 (39), 77 (28), 51 (16).

### 2,4-Bis-(4-methoxy-phenyl)-benzo[4,5]imidazo[1,2-a]pyrimidin-3-ylamine (3q)

Yellow solid; yield: 70%, mp 291–292 °C. IR (KBr) (*ν*_max_/cm^–1^): 3452 and 3369 (NH_2_), 1587, 1498, 1354, 1298, 1134, 1065, 1022, 983, 933, 886, 798, 702, 649. ^1^H NMR (300.1 MHz, DMSO-*d*_6_): *δ* 7.96 (d, *J* = 8.5 Hz, 2H, 2CH), 7.74 (d, *J* = 8.0 Hz, 2H, 2CH), 7.72 (d, *J* = 7.9 Hz, 1H, CH), 7.35–7.18 (m, 5H, 5CH), 6.90 (t, *J* = 7.8 Hz, 1H, CH), 6.11 (d, *J* = 8.4 Hz, 1H, CH), 4.14 (s, 2H, NH_2_), 3.76 and 3.75 (2 s, 6H, 2OCH_3_). ^13^C NMR (75.5 MHz, DMSO-*d*_6_): *δ* 160.9, 160.2, 156.5, 148.4, 144.7 and 136.2 (6C), 130.5 (CH), 129.7 (C), 129.2 (2CH), 128.6 (2CH), 128.2, 127.1 and 126.2 (3C), 124.8, 120.3 and 119.2 (3CH), 116.0 (2CH), 115.1 (2CH), 55.2 and 53.6 (2OCH_3_). EI-MS, *m/z* (%): 396 (M^+^, 75), 288 (36), 230 (100), 212 (14), 184 (27), 108 (29).

### 2-(4-Methoxy-phenyl)-4-thiophen-2-yl-benzo[4,5]imidazo[1,2-a]pyrimidin-3-ylamine (3r)

Yellow solid; yield: 82%, mp 274–277 °C. IR (KBr) (*ν*_max_/cm^–1^): 3488 and 3362 (NH_2_), 1596, 1503, 1487, 1363, 1278, 1243, 1137, 1041, 985, 962, 876, 795, 687, 632. ^1^H NMR (300.1 MHz, DMSO-*d*_6_): *δ* 8.24 (d, *J* = 3.8 Hz, 1H, CH), 7.90–7.68 (m, 4H, 4CH), 7.28–7.08 (m, 4H, 4CH), 6.94 (t, *J* = 7.8 Hz, 1H, CH), 6.17 (d, *J* = 8.4 Hz, 1H, CH), 4.24 (s, 2H, NH_2_), 3.91 (s, 3H, OCH_3_). ^13^C NMR (75.5 MHz, DMSO-*d*_6_): *δ* 159.2, 155.9, 150.4, 148.2, 144.8 and 141.8 (6C), 131.9 (CH), 131.8 (2CH), 131.6 (CH), 128.7, 127.4 and 126.5 (3C), 125.4, 124.9, 119.8 and 119.6 (4CH), 114.2 (2CH), 112.0 (CH), 54.3 (OCH_3_). EI-MS, *m/z* (%): 373 (M^+^ + 1, 100), 288 (16), 266 (72), 206 (38), 109 (49), 91 (14), 83 (23).

### 4-Phenyl-2-thiophen-2-yl-benzo[4,5]imidazo[1,2-a]pyrimidin-3-ylamine (3s)

Yellow solid; yield: 78%, mp 265–267 °C. IR (KBr) (*ν*_max_/cm^–1^): 3473 and 3345 (NH_2_), 1589, 1495, 1432, 1386, 1241, 1174, 1098, 1028, 934, 859, 743, 659. ^1^H NMR (300.1 MHz, DMSO-*d*_6_): *δ* 8.19 (d, *J* = 3.5 Hz, 1H, CH), 7.87 (d, *J* = 5.2 Hz, 1H, CH), 7.72 (d, *J* = 8.0 Hz, 1H, CH), 7.62–7.45 (m, 4H, 4CH), 7.30 (t, *J* = 7.4 Hz, 1H, CH), 7.24 (d, *J* = 4.6 Hz, 1H, CH), 6.95–6.75 (m, 2H, 2CH), 6.16 (d, *J* = 8.1 Hz, 1H, CH), 4.26 (s, 2H, NH_2_). ^13^C NMR (75.5 MHz, DMSO-*d*_6_): *δ* 155.3, 150.2, 147.7, 144.9, 141.7, 132.3 (6C), 131.6 (CH), 130.9 (2CH), 130.8 (CH), 129.5 (2CH), 128.6 (CH), 127.2 and 126.5 (2C), 125.3, 124.7, 119.9, 119.0 and 113.6 (5C). EI-MS, *m/z* (%): 342 (M^+^, 89), 258 (100), 228 (34), 189 (53), 177 (10), 91 (27), 82 (64).

### 2-Thiophen-2-yl-4-p-tolyl-benzo[4,5]imidazo[1,2-a]pyrimidin-3-ylamine (3t)

Yellow solid; yield: 69%, mp 276–277 °C. IR (KBr) (*ν*_max_/cm^–1^): 3474 and 3352 (NH_2_), 1585, 1522, 1486, 1448, 1346, 1220, 1188, 1072, 952, 899, 848, 774, 646, 623. ^1^H NMR (300.1 MHz, DMSO-*d*_6_): *δ* 8.19 (d, *J* = 3.5 Hz, 1H, CH), 7.89 (d, *J* = 5.0 Hz, 1H, CH), 7.70 (d, *J* = 8.2 Hz, 1H, CH), 7.58 (d, *J* = 8.4 Hz, 2H, 2CH), 7.55 (d, *J* = 8.3 Hz, 2H, 2CH), 7.38–7.22 (m, 2H, 2CH), 7.13–7.04 (m, 1H, CH), 6.90 (d, *J* = 8.4 Hz, 1H, CH), 4.26 (s, 2H, NH_2_), 2.29 (s, 3H, CH_3_). ^13^C NMR (75.5 MHz, DMSO-*d*_6_): *δ* 155.2, 150.2, 147.7, 144.9, 141.7, 140.7 and 132.4 (7C), 131.7 (CH), 130.9 (2CH), 129.5 (2CH), 128.7 (CH), 127.2 and 126.5 (2C), 125.3, 124.8, 119.9, 119.0 and 113.6 (5CH). EI-MS, *m/z* (%): 358 (M^+^ + 2, 100), 266 (8), 202 (45), 166 (32), 133 (24), 91 (63).

### 4-(4-Methoxy-phenyl)-2-thiophen-2-yl-benzo[4,5]imidazo[1,2-a]pyrimidin-3-ylamine (3u)

Yellow solid; yield: 72%, mp 271–273 °C. IR (KBr) (*ν*_max_/cm^–1^): 3468 and 3343 (NH_2_), 1568, 1483, 1353, 1279, 1234, 1149, 1098, 983, 886, 785, 683, 642. ^1^H NMR (300.1 MHz, DMSO-*d*_6_): *δ* 8.13 (d, *J* = 3.4 Hz, 1H, CH), 7.91 (d, *J* = 4.8 Hz, 1H, CH), 7.84 (d, *J* = 8.6 Hz, 2H, 2CH), 7.71 (d, *J* = 8.0 Hz, 1H, CH), 7.35–7.20 (m, 2H, 2CH), 7.11 (d, *J* = 8.6 Hz, 2H, 2CH), 6.90 (t, *J* = 7.6 Hz, 1H, CH), 6.31 (d, *J* = 8.4 Hz, 1H, CH), 4.24 (s, 2H, NH_2_), 3.82 (s, 3H, OCH_3_). ^13^C NMR (75.5 MHz, DMSO-*d*_6_): *δ* 159.9, 155.8, 150.4, 147.6, 144.9, 141.8 and 132.8 (7C), 131.5 and 128.7 (2CH), 128.6 (2CH), 127.6 and 126.9 (2C), 125.5, 124.8, 120.5 and 119.2 (4CH), 115.3 (2CH), 113.5 (CH), 55.8 (OCH3). EI-MS, *m/z* (%): 372 (M^+^, 58), 296 (23), 273 (42), 265 (100), 248 (15), 206 (34), 108 (76).

### 4-(4-Chloro-phenyl)-2-thiophen-2-yl-benzo[4,5]imidazo[1,2-a]pyrimidin-3-ylamine (3w)

Yellow solid; yield: 79%, mp 283–284 °C. IR (KBr) (*ν*_max_/cm^–1^): 3479 and 3326 (NH_2_), 1598, 1543, 1478, 1398, 1306, 1211, 1189, 1090, 973, 879, 837, 768, 723, 678, 641. ^1^H NMR (300.1 MHz, DMSO-*d*_6_): *δ* 8.17 (d, *J* = 2.9 Hz, 1H, CH), 7.87 (d, *J* = 5.4 Hz, 1H, CH), 7.83 (d, *J* = 8.3 Hz, 2H, 2CH), 7.74 (d, *J* = 8.3 Hz, 2H, 2CH), 7.70 (d, *J* = 7.8 Hz, 1H, CH), 7.31 (t, *J* = 7.8 Hz, 1H, CH), 7.25 (t, *J* = 3.3 Hz, 1H, CH), 6.93 (t, *J* = 7.6 Hz, 1H, CH), 6.18 (d, *J* = 8.3 Hz, 1H, CH), 4.36 (s, 2H, NH_2_). ^13^C NMR (75.5 MHz, DMSO-*d*_6_): *δ* 155.4, 150.3, 147.6, 144.9, 141.6 and 135.8 (6C), 131.9 (2CH), 131.6 (CH), 130.7 (C), 130.5 (2CH), 128.6 (CH), 128.4 and 127.1 (2C), 125.5, 124.7, 120.0, 118.9 and 113.3 (5CH). EI-MS, *m/z* (%): 376 (M^+^, 100), 341 (10), 266 (12), 240 (22), 205 (48), 170 (32), 138 (15), 102 (30), 75 (20), 51 (14).

### 2,4-Di-thiophen-2-yl-benzo[4,5]imidazo[1,2-a]pyrimidin-3-ylamine (3x)

Yellow solid, yield: 72%, mp 280–282 °C. IR (KBr) (*ν*_max_/cm^–1^): 3458 and 3306 (NH_2_), 1567, 1499, 1427, 1356, 1307, 1242, 1199, 1115, 1066, 960, 876, 824, 782, 716, 683, 642. ^1^H NMR (300.1 MHz, DMSO-*d*_6_): *δ* 8.21 (d, *J* = 3.2 Hz, 1H, CH), 8.07 (d, *J* = 4.3 Hz, 1H, CH), 7.89 (d, *J* = 4.4 Hz, 1H, CH), 7.87–7.67 (m, 3H, 3CH), 7.45–7.29 (m, 2H, 2CH), 7.06 (t, *J* = 7.6 Hz, 1H, CH), 6.62 (d, *J* = 8.3 Hz, 1H, CH), 4.43 (s, 2H, NH_2_). ^13^C NMR (75.5 MHz, DMSO-*d*_6_): *δ* 155.7, 155.4, 151.0, 144.8, 142.7 and 142.4 (6C), 132.8 (CH), 131.1 (C), 130.9 130.4, 129.1 and 128.2 (4CH), 127.4 (C), 125.6, 121.0, 119.2, 118.9 and 114.0 (5CH). EI-MS, *m/z* (%): 348 (M^+^, 100), 272 (39), 266 (15), 182 (64), 174 (18), 82 (43).

### 7,8-Dichloro-2,4-diphenyl-benzo[4,5]imidazo[1,2-a]pyrimidin-3-ylamine (3y)

Yellow solid, yield: 70%, mp 267–270 °C. IR (KBr) (*ν*_max_/cm^–1^): 3464 and 3325 (NH_2_), 1597, 1539, 1501, 1419, 1360, 1295, 1190, 1067, 966, 906, 879, 753, 683, 642. ^1^H NMR (300.1 MHz, DMSO-*d*_6_): *δ* 8.13 (dd, *J* = 2.1, 7.3 Hz, 2H, 2CH), 8.01 (s, 1H, CH), 7.89 (dd, *J* = 2.4, 7.5 Hz, 1H, CH), 7.79 (t, *J* = 7.4 Hz, 2H, 2CH), 7.62–7.45 (m, 5H, 5CH), 6.12 (s, 1H, CH), 4.23 (s, 2H, NH_2_). ^13^C NMR (75.5 MHz, DMSO-*d*_6_): *δ* 157.3, 149.6, 144.4, 136.6, 136.4 (5C), 129.8 (2CH), 129.72 (2CH), 129.69 (CH), 129.6 (2CH), 128.71 (C), 128.67 and 128.6 (2CH), 128.1 (2CH), 127.5, 127.2, 126.6 and 126.2 (4C), 119.9 (CH). EI-MS, *m/z* (%): 405 (M^+^ + 1, 100), 327 (34), 250 (75), 233 (66), 170 (29), 133 (22), 76 (34).

### 7,8-Dichloro-4-(4-chloro-phenyl)-2-phenyl-benzo[4,5]imidazo[1,2-a]pyrimidin-3-ylamine (3z)

Yellow solid, yield: 74%, mp 291–293 °C. IR (KBr) (*ν*_max_/cm^–1^): 3461 and 3384 (NH_2_), 1585, 1532, 1486, 1425, 1386, 1220, 1188, 1082, 1020, 958, 848, 784, 646, 621. ^1^H NMR (300.1 MHz, DMSO-*d*_6_): *δ* 8.00 (s, 1H, CH), 7.89–7.82 (m, 4H, 4CH), 7.74 (d, *J* = 8.5 Hz, 2H, 2CH), 7.63–7.46 (m, 3H, 3CH), 6.22 (s, 1H, CH), 4.34 (s, 2H, NH_2_). ^13^C NMR (75.5 MHz, DMSO-*d*_6_): *δ* 157.3, 149.2, 143.8, 136.6, 135.9 (5C), 132.0 (2CH), 130.6 (2CH), 130.2 (CH), 129.7 (C), 128.73 (2CH), 128.66 (2CH), 128.2, 127.9 and 127.7 (3C), 127.6 (CH), 127.2 and 126.2 (2C), 120.0 (CH). EI-MS, *m/z* (%): 438 (M^+^, 43), 362 (100), 328 (22), 248 (72), 182 (36), 144 (12), 111 (24), 76 (10).

### 7,8-Dichloro-2-(4-chloro-phenyl)-4-phenyl-benzo[4,5]imidazo[1,2-a]pyrimidin-3-ylamine (3aa)

Yellow solid, yield: 75%, mp 302–304 °C. IR (KBr) (*ν*_max_/cm^–1^): 3419 and 3329 (NH_2_), 1578, 1483, 1426, 1353, 1279, 1204, 1149, 1092, 963, 876, 832, 775, 683. ^1^H NMR (300.1 MHz, DMSO-*d*_6_): *δ* 8.15 (d, *J* = 8.6 Hz, 2H, 2CH), 7.99 (s, 1H, CH), 7.74–7.58 (m, 5H, 5CH), 7.45 (d, *J* = 8.6 Hz, 2H, 2CH), 6.15 (s, 1H, CH), 4.28 (s, 2H, NH_2_). ^13^C NMR (75.5 MHz, DMSO-*d*_6_): *δ* 157.3, 149.1, 144.4, 135.2 and 134.9 (5C), 131.3 (2CH), 130.5 (CH), 129.8 (2CH), 129.3 (2CH), 129.2 and 128.7 (2C), 128.6 (CH), 127.9 (2CH), 127.3, 127.2, 126.4 and 126.2 (4C), 119.8 (CH). EI-MS, *m/z* (%): 436 (M^+^-2, 100), 360 (26), 328 (68), 249 (28), 184 (10), 111 (22), 82 (43).

### 7,8-Dichloro-2-(4-chloro-phenyl)-4-p-tolyl-benzo[4,5]imidazo[1,2-a]pyrimidin-3-ylamine (3ab)

Yellow solid, yield: 81%, mp 289–290 °C. IR (KBr) (*ν*_max_/cm^–1^): 3486 and 3362 (NH_2_), 1588, 1487, 1359, 1291, 1140, 1088, 959, 929, 846, 797, 683, 642. ^1^H NMR (300.1 MHz, DMSO-*d*_6_): *δ* 8.15 (d, *J* = 8.6 Hz, 2H, 2CH), 8.02 (s, 1H, CH), 7.58 (d, *J* = 8.2 Hz, 2H, 2CH), 7.57 (d, *J* = 8.4 Hz, 2H, 2CH), 7.50 (d, *J* = 8.2 Hz, 2H, 2CH), 6.24 (s, 1H, CH), 4.27 (s, 2H, NH_2_), 2.47 (s, 3H, CH_3_). ^13^C NMR (75.5 MHz, DMSO-*d*_6_): *δ* 157.3, 149.4, 144.4, 137.0, 136.5 and 135.2 (6C), 131.50 (2CH), 131.46 (CH), 130.7 (C), 130.0 (2CH), 129.6 (2CH), 129.2 and 128.6 (2C), 128.2 (2CH), 127.6, 126.6 and 125.6 (3C), 119.9 (CH), 21.1 (CH_3_). EI-MS, *m/z* (%): 452 (M^+^, 100), 308 (42), 266 (15), 218 (73), 202 (42), 133 (15), 111 (18), 91 (26).

### 7,8-Dichloro-2-(4-chloro-phenyl)-4-(4-methoxy-phenyl)-benzo[4,5]imidazo[1,2-a]pyrimidin-3-ylamine (3ac)

Yellow solid, yield: 75%, mp 294–295 °C. IR (KBr) (*ν*_max_/cm^–1^): 3466 and 3394 (NH_2_), 1579, 1498, 1362, 1294, 1247, 1183, 1096, 1043, 954, 879, 812, 772, 739, 690, 627. ^1^H NMR (300.1 MHz, DMSO-*d*_6_): *δ* 7.98 (s, 1H, CH), 7.86 (d, *J* = 8.9 Hz, 2H, 2CH), 7.77 (d, *J* = 8.5 Hz, 2H, 2CH), 7.61 (d, *J* = 8.5 Hz, 2H, 2CH), 6.98 (d, *J* = 8.9 Hz, 2H, 2CH), 6.50 (s, 1H, CH), 4.22 (s, 2H, NH_2_), 3.74 (s, 3H, OCH_3_). ^13^C NMR (75.5 MHz, DMSO-*d*_6_): *δ* 160.6, 157.3, 149.5, 144.8, 137.3 and 135.3 (6C), 132.7 (2CH), 131.3 (2CH), 131.0 (C), 130.1 (CH), 129.3 and 128.8 (2C), 128.6 (2CH), 127.4, 126.3 and 125.5 (3C), 119.9 (CH), 114.1 (2CH), 55.3 (OCH_3_). EI-MS, *m/z* (%): 468 (M^+^, 88), 357 (43), 346 (100), 234 (15), 133 (62), 125 (10), 108 (15), 51 (24).

### 7,8-Dichloro-2,4-bis-(4-chloro-phenyl)-benzo[4,5]imidazo[1,2-a]pyrimidin-3-ylamine (3ad)

Yellow solid, yield: 82%, mp 308–310 °C. IR (KBr) (*ν*_max_/cm^–1^): 3435 and 3384 (NH_2_), 1505, 1461, 1370, 1287, 1123, 1090, 965, 835, 791, 725, 646, 634. ^1^H NMR (300.1 MHz, DMSO-*d*_6_): *δ* 8.25 (s, 1H, CH), 8.03 (d, *J* = 8.5 Hz, 2H, 2CH), 7.93 (d, *J* = 8.4 Hz, 2H, 2CH), 7.82 (d, *J* = 8.5 Hz, 2H, 2CH), 7.72 (d, *J* = 8.4 Hz, 2H, 2CH), 6.24 (s, 1H, CH), 4.30 (s, 2H, NH_2_). ^13^C NMR (75.5 MHz, DMSO-*d*_6_): *δ* 157.6, 149.5, 144.7, 137.3 and 135.7 (5C), 132.1 (2CH), 131.6 (2CH), 131.3 (C), 130.6 (CH), 129.9 (2CH), 129.5, 128.8 and 128.6 (3C), 128.4 (2CH), 127.2, 126.3 and 125.6 (3C), 119.9 (CH). EI-MS, *m/z* (%): 480 (M^+^, 100), 370 (37), 250 (45), 112 (67), 78 (19), 51 (25). EI-MS, *m/z* (%): 474 (M^+^, 100), 360 (49), 288 (10), 249 (34), 238 (26), 133 (16), 111 (38).

### 7,8-Dichloro-2-(4-chloro-phenyl)-4-thiophen-2-yl-benzo[4,5]imidazo[1,2-a]pyrimidin-3-ylamine (3ae)

Yellow solid, yield: 73%, mp 275–278 °C. IR (KBr) (*ν*_max_/cm^–1^): 3489 and 3347 (NH_2_), 1585, 1522, 1486, 1448, 1346, 1340, 1288, 1173, 1062, 952, 949, 858, 774, 701, 646. ^1^H NMR (300.1 MHz, DMSO-*d*_6_): *δ* 8.15 (d, *J* = 3.1 Hz, 1H, CH), 7.95–7.82 (m, 4H, 4CH), 7.79 (s, 1H, CH), 7.76 (d, *J* = 2.0 Hz, 1H, CH), 6.11 (s, 1H, CH), 4.47 (s, 2H, NH_2_). ^13^C NMR (75.5 MHz, DMSO-*d*_6_): *δ* 157.3, 150.8, 148.8, 144.1, 141.1 and 136.1 (6C), 132.4 (CH), 132.0 (2CH), 131.3 (CH), 130.6 (2CH), 130.3, 128.7, 127.7 and 127.2 (4C), 126.33 (CH), 126.25 (C), 120.6 and 114.5 (2CH). EI-MS, *m/z* (%): 444 (M^+^, 62), 360 (24), 332 (100), 266 (15), 208 (52), 113 (29), 82 (10).

### 2-(4-Bromo-phenyl)-7,8-dichloro-4-phenyl-benzo[4,5]imidazo[1,2-a]pyrimidin-3-ylamine (3af)

Yellow solid, yield: 84%, mp 293–296 °C. IR (KBr) (*ν*_max_/cm^–1^): 3487 and 3328 (NH_2_), 1588, 1521, 1487, 1341, 1310, 1221, 1168, 1072, 978, 924, 851, 767, 697, 642. ^1^H NMR (300.1 MHz, DMSO-*d*_6_): *δ* 8.09 (d, *J* = 8.6 Hz, 2H, 2CH), 8.01 (s, 1H, CH), 7.74–7.64 (m, 7H, 7CH), 6.13 (s, 1H, CH), 4.29 (s, 2H, NH_2_). ^13^C NMR (75.5 MHz, DMSO-*d*_6_): *δ* 157.4, 149.4, 144.5, 136.6 and 135.4 (5C), 131.7 (2CH), 131.6 (C), 131.1 (2CH), 130.7 (CH), 129.8 (2CH), 129.7 (C), 129.5 (2CH), 128.9 (CH), 127.5, 127.3, 126.5 and 123.8 (4C), 119.9 (CH). EI-MS, *m/z* (%): 482 (M^+^, 100), 389 (23), 327 (44), 234 (86), 182 (64), 174 (18), 77 (15).

### 2-(4-Bromo-phenyl)-7,8-dichloro-4-p-tolyl-benzo[4,5]imidazo[1,2-a]pyrimidin-3-ylamine (3ag)

Yellow solid, yield: 76%, mp 306–308 °C. IR (KBr) (*ν*_max_/cm^–1^): 3467 and 3343 (NH_2_), 1578, 1483, 1353, 1279, 1204, 1149, 1092, 963, 876, 832, 775, 743, 663. ^1^H NMR (300.1 MHz, DMSO):* δ* 8.05 (d, *J* = 8.6 Hz, 2H, 2CH), 7.95 (s, 1H, CH), 7.68 (d, *J* = 8.6 Hz, 2H, 2CH), 7.58 (d, *J* = 8.0 Hz, 2H, 2CH), 7.50 (d, *J* = 8.0 Hz, 2H, 2CH), 6.22 (s, 1H, CH), 4.26 (s, 2H, NH_2_), 2.48 (s, 3H, CH_3_). ^13^C NMR (75.5 MHz, DMSO-*d*_6_): *δ* 157.3, 149.4, 144.4, 136.84, 136.78 and 135.3 (6C), 131.7 (2CH), 131.6 (CH), 131.1 (2CH), 130.8 (C), 130.0 (2CH), 129.7 (2CH), 129.5, 127.6, 126.5, 125.9 and 123.8 (5C), 119.9 (CH), 21.2 (CH_3_). EI-MS, *m/z* (%): 496 (M^+^, 100), 342 (25), 326 (74), 263 (14), 167 (48), 133 (25), 79 (33), 51 (18).

### 3-Chloro-2,4-diphenyl-benzo[4,5]imidazo[1,2-a]pyrimidine (4a)

Yellow solid, yield: 56%, mp 228–229 °C. IR (KBr) (*ν*_max_/cm^–1^): 1578, 1529, 1474, 1437, 1376, 1297, 1251, 1214, 1175, 1091, 1026, 954, 908, 834, 805, 728, 637. ^1^H NMR (300.1 MHz, DMSO):* δ* 7.64 (dd, *J* = 1.6, 7.2 Hz, 2H, 2CH), 7.45–7.20 (m, 9H, 9CH), 7.00 (t, *J* = 7.8 Hz, 1H, CH), 6.86 (t, *J* = 7.6 Hz, 1H, CH), 6.32 (d, *J* = 8.1 Hz, 1H, CH). ^13^C NMR (75.5 MHz, DMSO-*d*_6_): *δ* 156.7, 143.1, 136.8, 135.3 and 131.2 (5C), 130.6 (CH), 130.3 (2CH), 129.5 (CH), 129.4 (2 × 2CH), 128.7 (2CH), 128.5 (C), 127.8 (CH), 126.2 and 126.1 (2C), 124.9, 120.2 and 119.2 (3CH). EI-MS, *m/z* (%): 355 (M^+^, 100), 204 (48), 145 (52), 167 (75), 77 (32), 51 (25).

### 2,4-Diphenyl-benzo[4,5]imidazo[1,2-a]pyrimidine (6a)

Yellow solid, yield: 65%, mp 206–208 °C. IR (KBr) (*ν*_max_/cm^–1^): 1603, 1505, 1461, 1370, 1302, 1250, 1187, 1090, 1048, 1006, 965, 897, 834, 785, 732, 634. ^1^H NMR (300.1 MHz, DMSO):* δ* 8.40 (d, *J* = 8.0 Hz, 2H, 2CH), 7.90–7.35 (m, 9H, 9CH), 7.29 (s, 1H, CH), 7.05 (t, *J* = 7.8 Hz, 1H, CH), 6.93 (t, *J* = 7.5 Hz, 1H, CH), 6.53 (d, *J* = 7.9 Hz, 1H, CH). ^13^C NMR (75.5 MHz, DMSO-*d*_6_): *δ* 159.9, 152.4, 144.5, 138.6, 136.4 and 131.8 (6C), 130.8 and 130.4 (2CH), 130.2 (2CH), 130.0 (2CH), 129.7 (2CH), 128.6 (CH), 128.2 (2CH), 127.8 and 126.9 (2C), 125.4, 120.4, 118.7 and 108.5 (4CH). EI-MS, *m/z* (%): 321 (M^+^, 100), 244 (18), 167 (25), 79 (65), 52 (45).

### *Saccharomyces cerevisiae α*-glucosidase inhibition assay

*α*-Glucosidase enzyme ((EC3.2.1.20, Saccharomyces cerevisiae, 20 U/mg) and substrate (p-nitrophenyl glucopyranoside) were purchased from Sigma-Aldrich. Enzyme was prepared in potassium phosphate buffer (pH 6.8, 50 mM), and ploy-substituted-2,4-diarylbenzo[4,5]imidazo[1,2-*a*]pyrimidines **3a–ag**, **4a**, and **6a** was dissolved in DMSO (10% final concentration). The various concentrations of these compounds (20 mL), enzyme solution (20 mL) and potassium phosphate buffer (135 mL), were added in the 96-well plate and incubated at 37 °C for 10 min. Then, the substrate (25 mL, 4 mM) was added to the mentioned mixture and allowed to incubate at 37 °C for 20 min. Finally, the change in absorbance was measured at 405 nm by using spectrophotometer (Gen5, Power wave xs2, BioTek, America). DMSO (10% final concentration) and acarbose were used respectively as control and standard drug. The percentage of enzyme inhibition was calculated and IC_50_ values were obtained from non-linear regression curve using the Logit method^67^. The statistical analyses were provided using SigmaStat 14.0 (Systat Software, Inc | Tools For Science).

### Kinetic studies

The kinetic analysis was carried out to determine inhibition mode of most potent compound **3k**. The 20 mL of enzyme solution (1 U/mL) was incubated with different concentrations (0, 45, 65, and 80 mM) of compound **3k** for 15 min at 30 °C. The reaction was then started by adding different concentrations of substrate (p-nitrophenyl glucopyranoside,1–4 mM), and change in absorbance was measured for 20 min at 405 nm by using spectrophotometer (Gen5, Power wave xs2, BioTek, America).

### Rat *α*-glucosidase assay

Rat small intestine *α*-glucosidase (EC 3.2.1.20) was prepared according to the method provided by Lossow et al. (1964). Enzyme in vitro activity was determined by recording the release of 4-nitrophenol from Pnitrophenyl α-D glucopyranoside according to the method described by Kim^68,69^. Final volume of 200 μL of assay solution was prepared in a 96-well plate as follow: the enzyme solution (190 μL, 0.15 units/ml), different concentrations of ploy-substituted-2,4-diarylbenzo[4,5]imidazo[1,2-*a*]pyrimidines **3a–ag**, **4a**, and **6a** (1, 10, 20, 50, 100, 500 and 1000 μM (5 μL)), and potassium phosphate buffer. Test compounds were dissolved in DMSO (not exceed than 5% of final volume). After 10 min. of pre-incubation at 37 ^◦^C, p-nitrophenyl glucopyranoside as substrate (5 μL, 3 mM), was added to the enzyme solution and let to be incubated for one hour at 37 ^◦^C. Finally, the change in the absorbance was followed at 405 nm using Cytation 3 hybrid microplate reader (BioTek, USA). DMSO and acarbose were used as the control and standard inhibitor, respectively. IC_50_ values of tested compounds were obtained from the nonlinear regression curve using GraphPadprism 6.0 (San Diego, California, USA) (https://www.graphpad.com/scientific-software/prism/).

All experimental animal procedures were approved by the Animal Care, use Ethics Committee at Shahid Beheshti University of Medical Sciences (SBMU), and comply with the Animal Research Reporting of In Vivo Experiments (ARRIVE) guidelines. All methods proposed here were performed in accordance with relevant institutional guidelines and regulations.

### Molecular docking study

Since the X-ray crystallographic structure *S. cerevisiae* α-glucosidase isn’t accessible, the 3D structure of *S.cerevisiae isomaltase* with PDB ID: 3A4A was downloaded from RCSB web site with 84% similarity to *S. cerevisiae* α-glucosidase^15^.

Docking studies were performed based on previous studies^34,70,71^ using Auto Dock Tools (version1.5.6), and the pdb structure of 3A4A and 3TOP were taken from the Brookhaven protein database (http://www.rcsb.org). The 3D structures of the selected compounds were created by MarvineSketch 5.8.3, 2012, ChemAxon (http://www.chemaxon.com) and converted to pdbqt coordinate using Auto dock Tools. Before preparation of auto dock format of protein, the water molecules and the inhibitors were removed from it. Then, using Auto Dock Tools, polar hydrogen atoms were added, Kollman charges were assigned, and the obtained enzyme structure was used as an input file for the AUTOGRID program. In AUTOGRID for each atom type in the ligand, maps were calculated with 0.375 A spacing between grid points, and the center of the grid box was placed at x = 22.625, y = − 8.069, and z = 24.158 for 3A4A and x = − 51.5, y = 9, and z = − 64.8 for 3TOP. The dimensions of the active site box were set at 50 × 50 × 50 A. Each docked system was carried out by 150 runs of the AUTODOCK search by the Lamarckian genetic algorithm. The best pose of each ligand was selected for analyzing the interactions between *α*-glucosidase and the inhibitor. The results were visualized using Discovery Studio 4.0 Client (https://discover.3ds.com/discovery-studio-visualizer-download) and LigPlot (https://www.ebi.ac.uk/thornton-srv/software/LigPlus/download.html) (Figs. 3, 4, 5, 6, 7).

## Supplementary Information

Supplementary Information.
