# Supplementary material for: Design, synthesis, molecular docking, and in vitro α-glucosidase inhibitory activities of novel 3-amino-2,4-diarylbenzo[4,5]imidazo[1,2-a]pyrimidines against yeast and rat α-glucosidase
Source: Sci Rep. 2021 Jun 7;11:11911. doi: 10.1038/s41598-021-91473-z (PMC8184976; doi:10.1038/s41598-021-91473-z)

Design, synthesis, molecular docking, and *in vitro* *α*-glucosidase inhibitory activities of novel 3-amino-2,4-diarylbenzo[4,5]imidazo[1,2-*a*]pyrimidines against yeast and rat *α*-glucosidase

Fariba Peytam[a], Ghazaleh Takalloobanafshi[b], Toktam Saadattalab[c], Maryam Norouzbahari[d], Zahra Emamgholipour[c], Setareh Moghimi[a], Loghman Firoozpour[a], Hamid Reza Bijanzadeh[e], Mohammad Ali Faramarzi[f], Somayeh Mojtabavi[f], Parviz Rashidi-Ranjbar[b], Saeed Karima [g], Roya Pakraad [g], Alireza Foroumadi[a,c,*]

[a] Drug Design and Development Research Center, The Institute of Pharmaceutical Sciences (TIPS), Tehran University of Medical Sciences, Tehran, Iran

[b] School of Chemistry, College of Science, University of Tehran, Tehran, Iran

[c] Department of Medicinal Chemistry, Faculty of Pharmacy, Tehran University of Medical Sciences, Tehran, Iran

[d] Faculty of Medicine, Eastern Mediterranean University, Famagusta, Northern Cyprus, via Mersin 10, Turkey.

[e] Department of Environmental Sciences, Faculty of Natural Resources and Marine Sciences, Tarbiat Modares University, Tehran, Iran

[f] Department of Pharmaceutical Biotechnology, Faculty of Pharmacy, Tehran University of Medical Sciences, Tehran, Iran

[g] Department of Clinical Biochemistry, School of Medicine, Shahid Beheshti University of Medical Sciences (SBMU), Tehran, Iran

^{1}H NMR spectrum of 2,4-diphenyl-benzo[4,5]imidazo[1,2-a]pyrimidin-3-ylamine (**3a**)

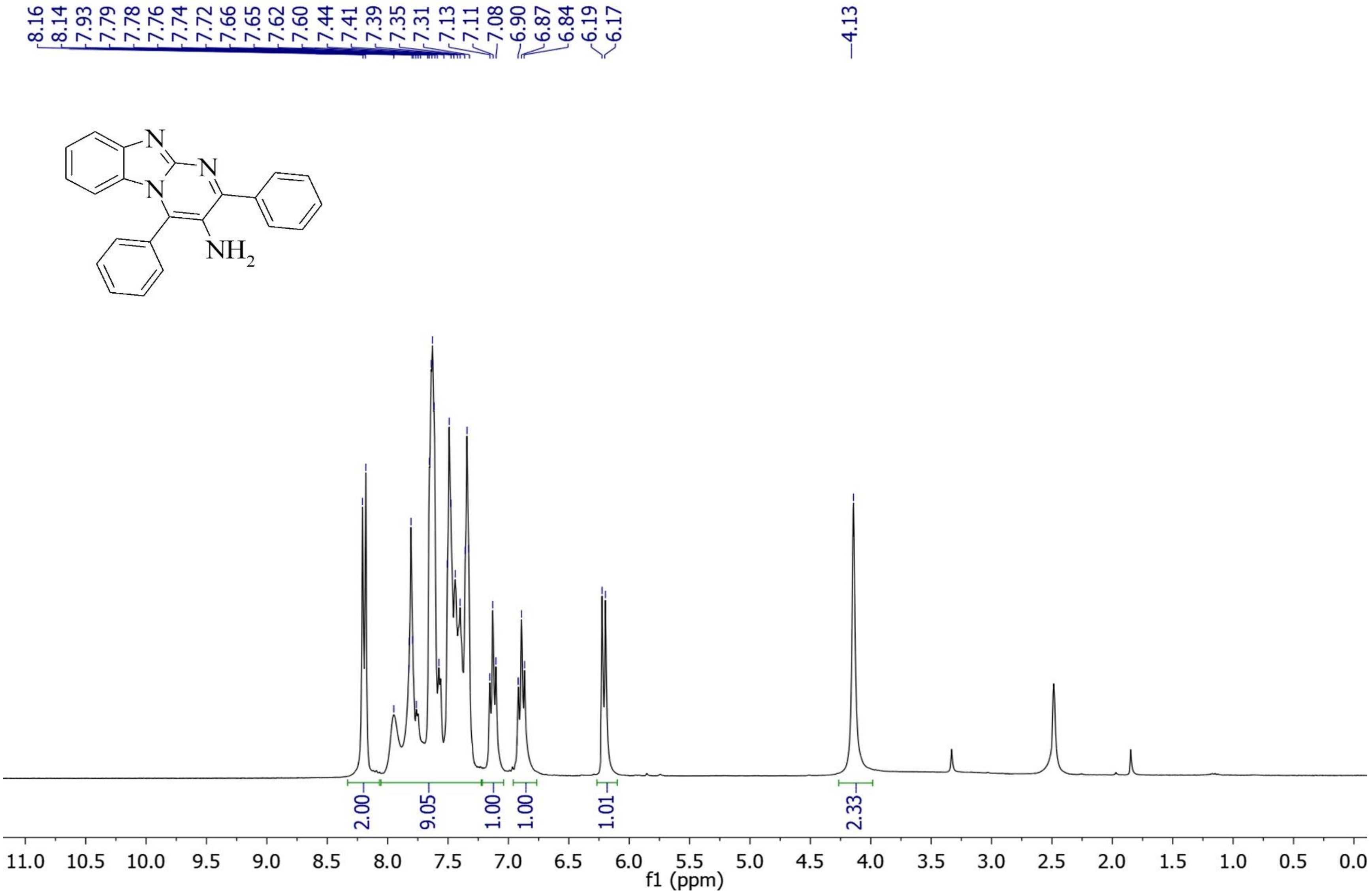

^{13}C NMR spectrum of 2,4-diphenyl-benzo[4,5]imidazo[1,2-a]pyrimidin-3-ylamine (**3a**)

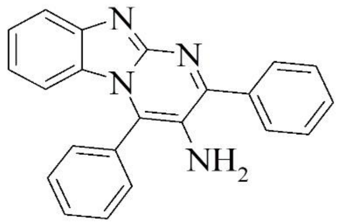

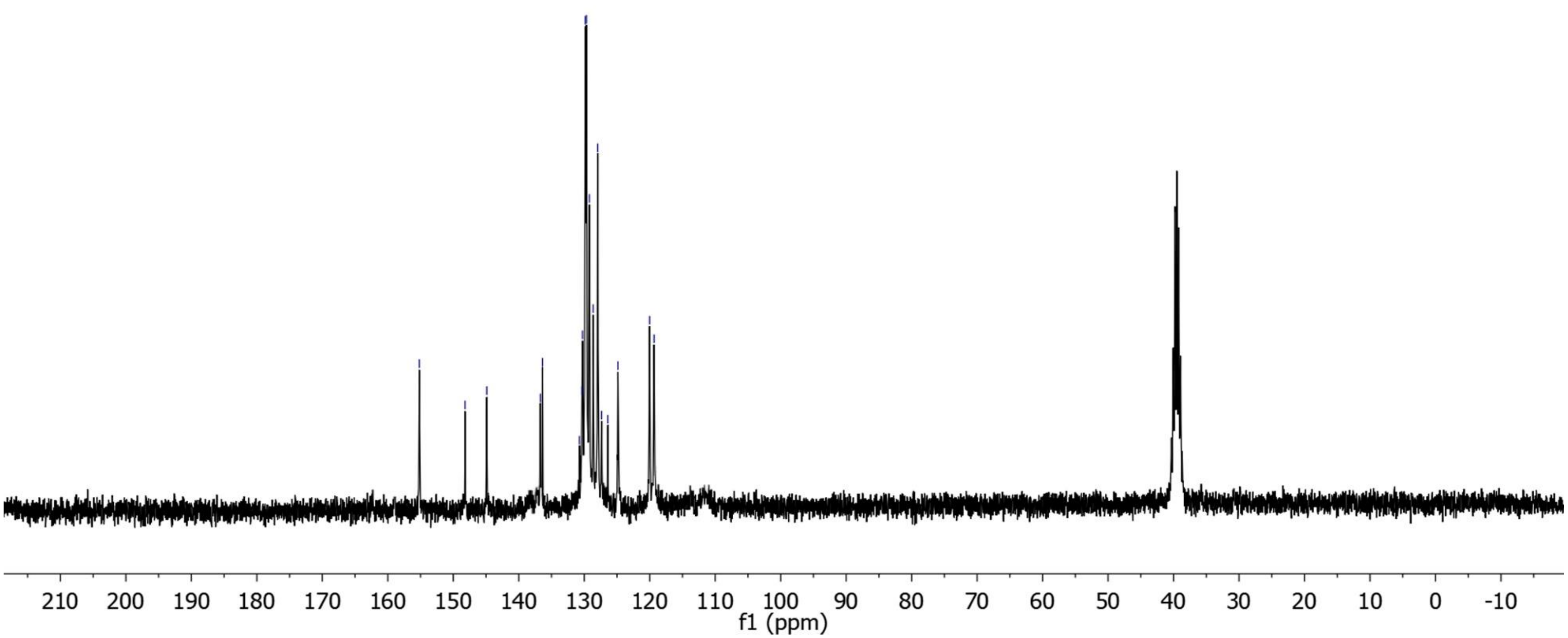

^{1}H NMR spectrum of 2-phenyl-4-p-tolyl-benzo[4,5]imidazo[1,2-a]pyrimidin-3-ylamine (**3b**)

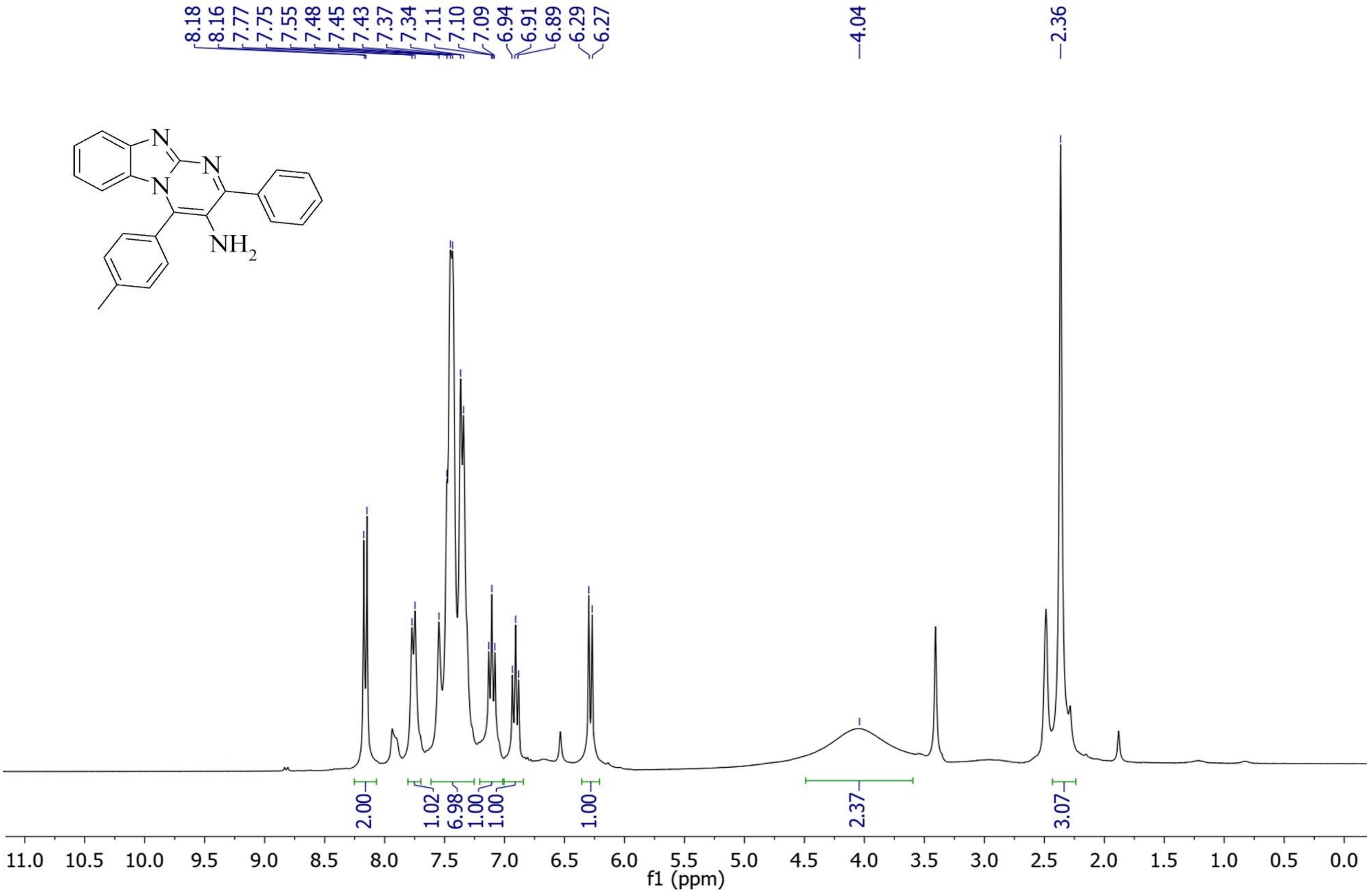

^{13}C NMR spectrum of 2-phenyl-4-p-tolyl-benzo[4,5]imidazo[1,2-a]pyrimidin-3-ylamine (**3b**)

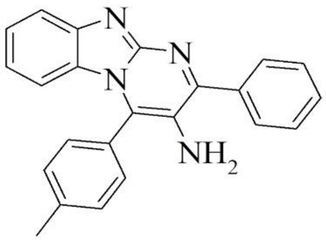

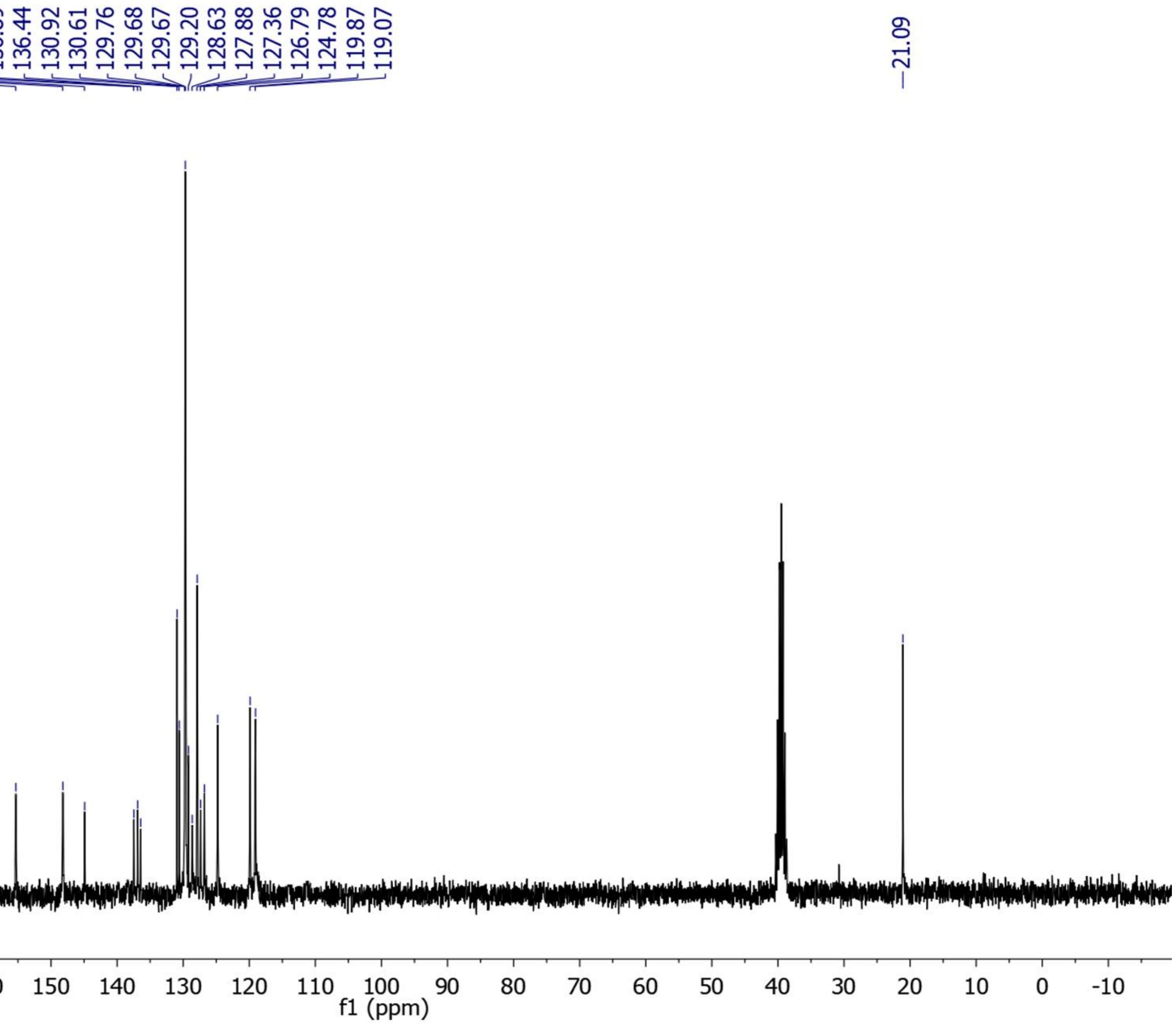

^{1}H NMR spectrum of 4-(4-methoxy-phenyl)-2-phenyl-benzo[4,5]imidazo[1,2-a]pyrimidin-3-ylamine (**3c**)

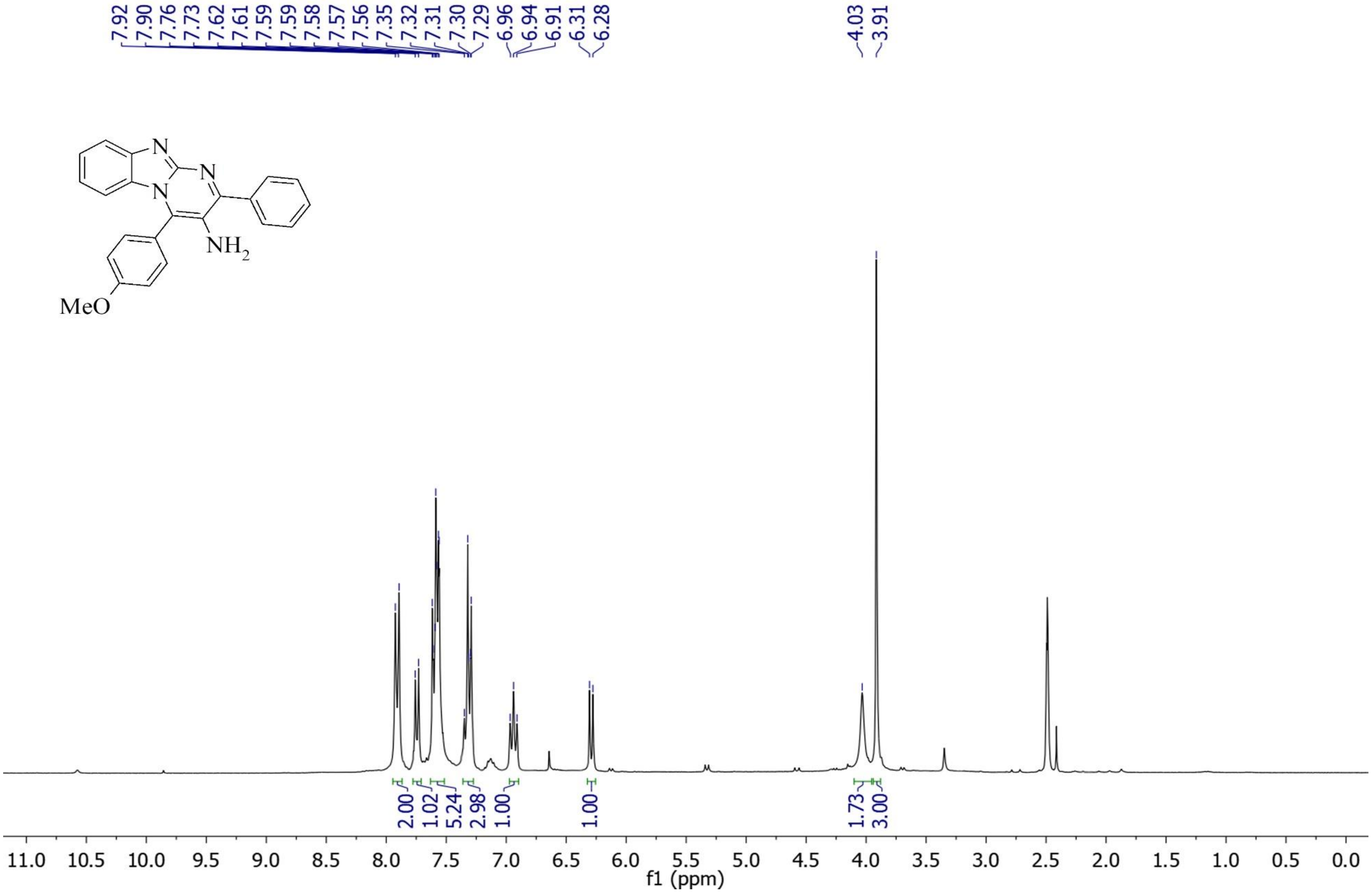

^{13}C NMR spectrum of 4-(4-methoxy-phenyl)-2-phenyl-benzo[4,5]imidazo[1,2-a]pyrimidin-3-ylamine (**3c**)

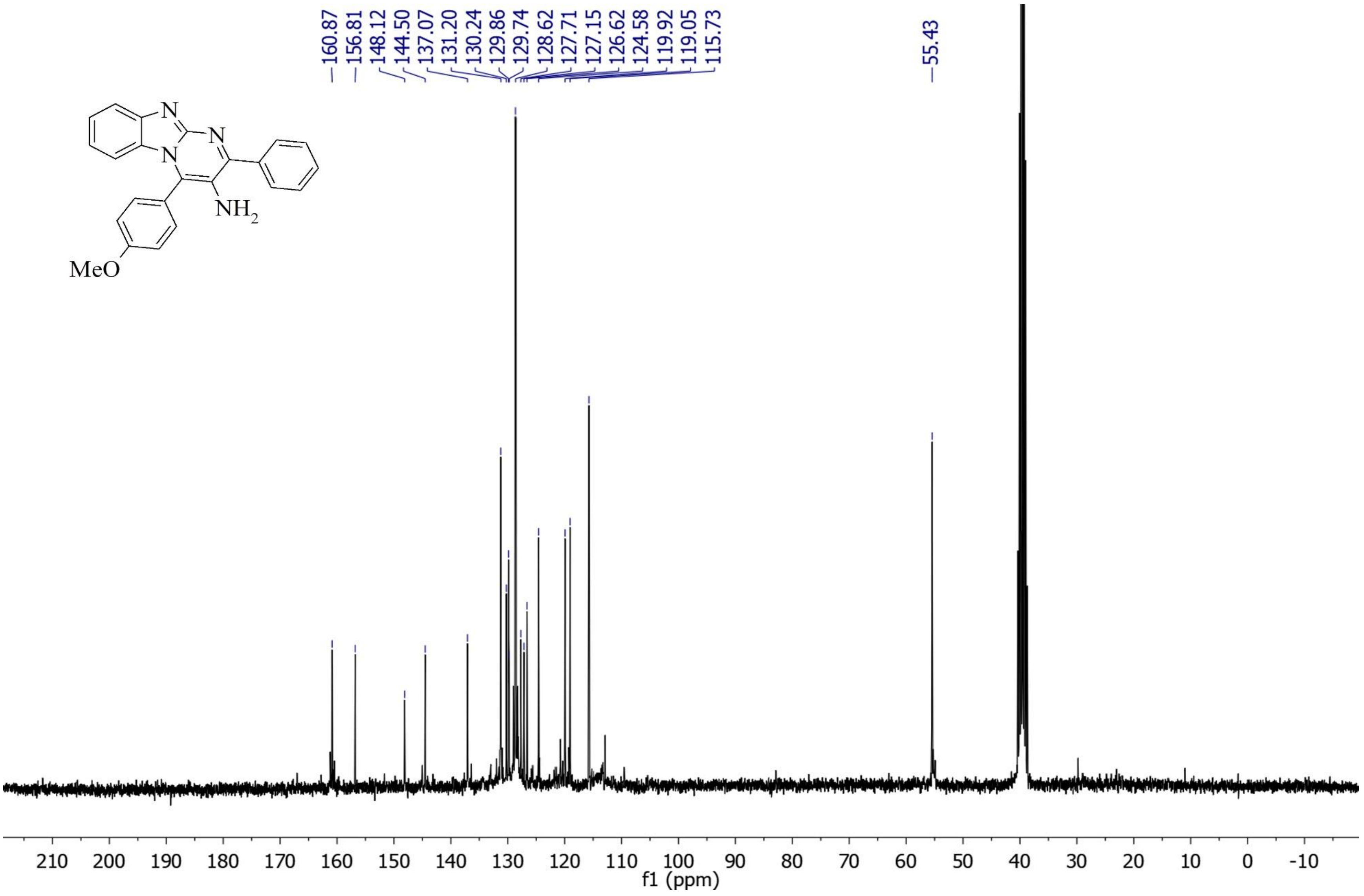

^{1}H NMR spectrum of 4-(4-chloro-phenyl)-2-phenyl-benzo[4,5]imidazo[1,2-a]pyrimidin-3-ylamine (**3d**)

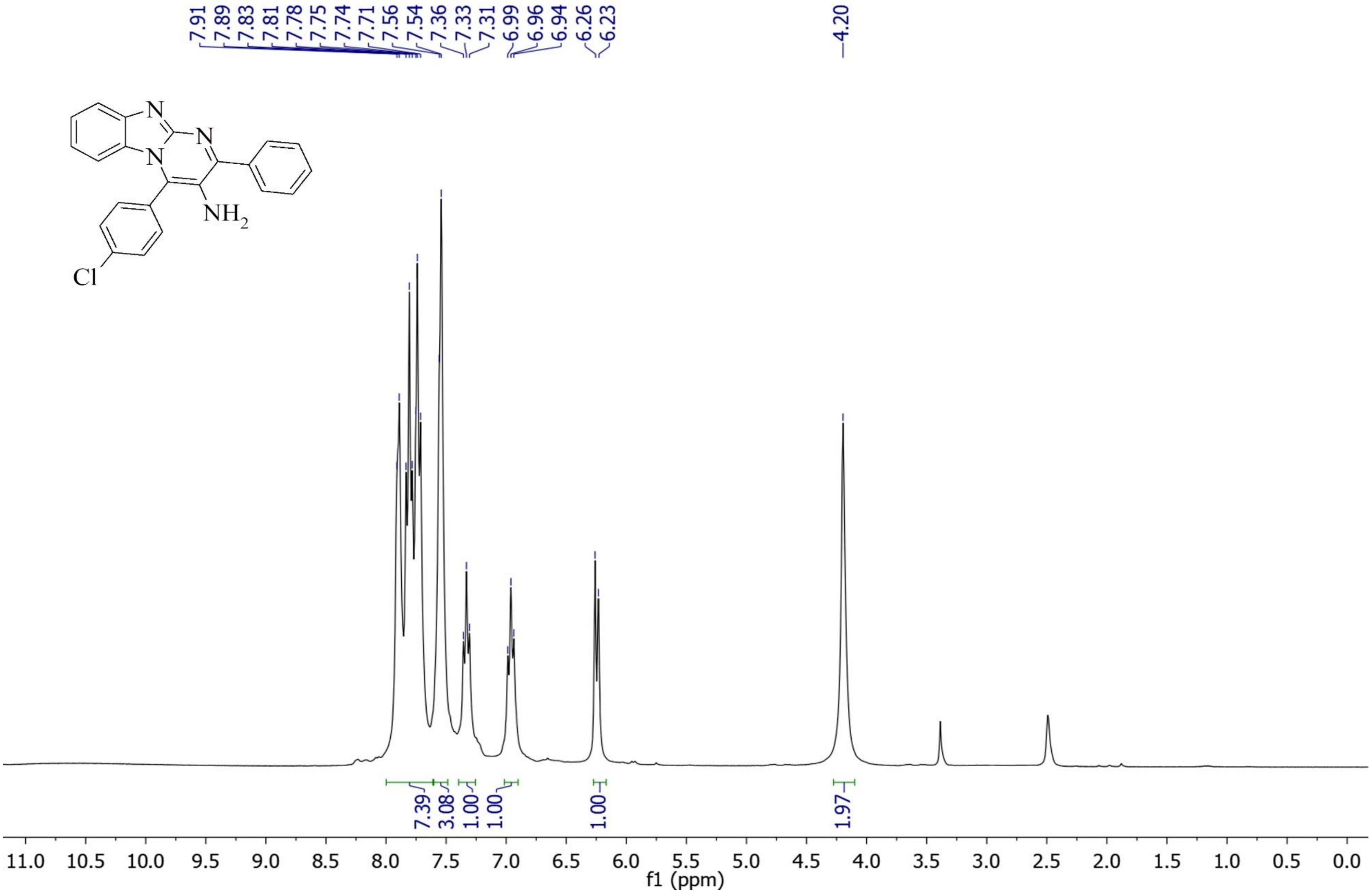

^{13}C NMR spectrum of 4-(4-chloro-phenyl)-2-phenyl-benzo[4,5]imidazo[1,2-a]pyrimidin-3-ylamine (**3d**)

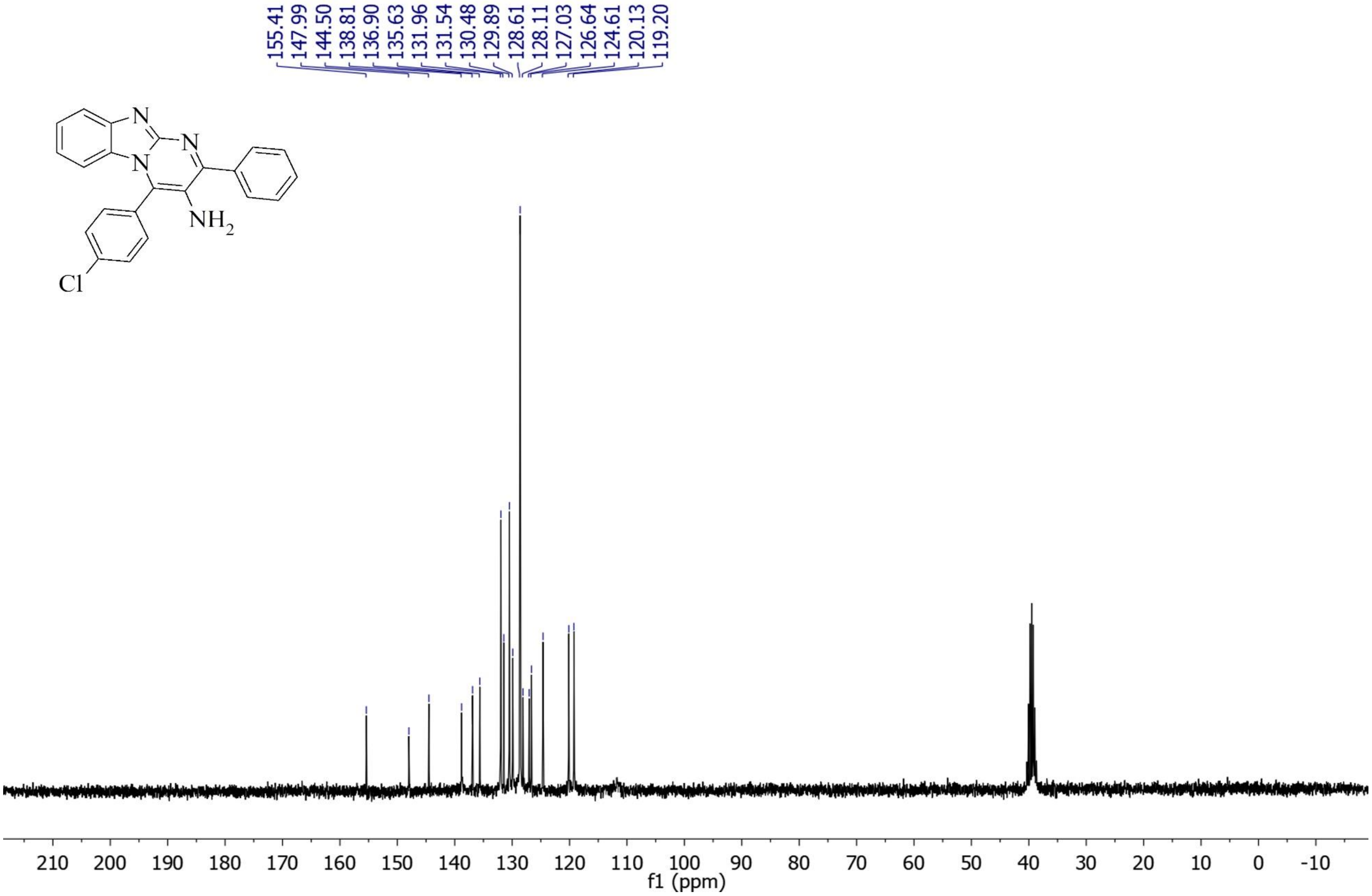

^{1}H NMR spectrum of 4-(2-chloro-phenyl)-2-phenyl-benzo[4,5]imidazo[1,2-a]pyrimidin-3-ylamine (**3e**)

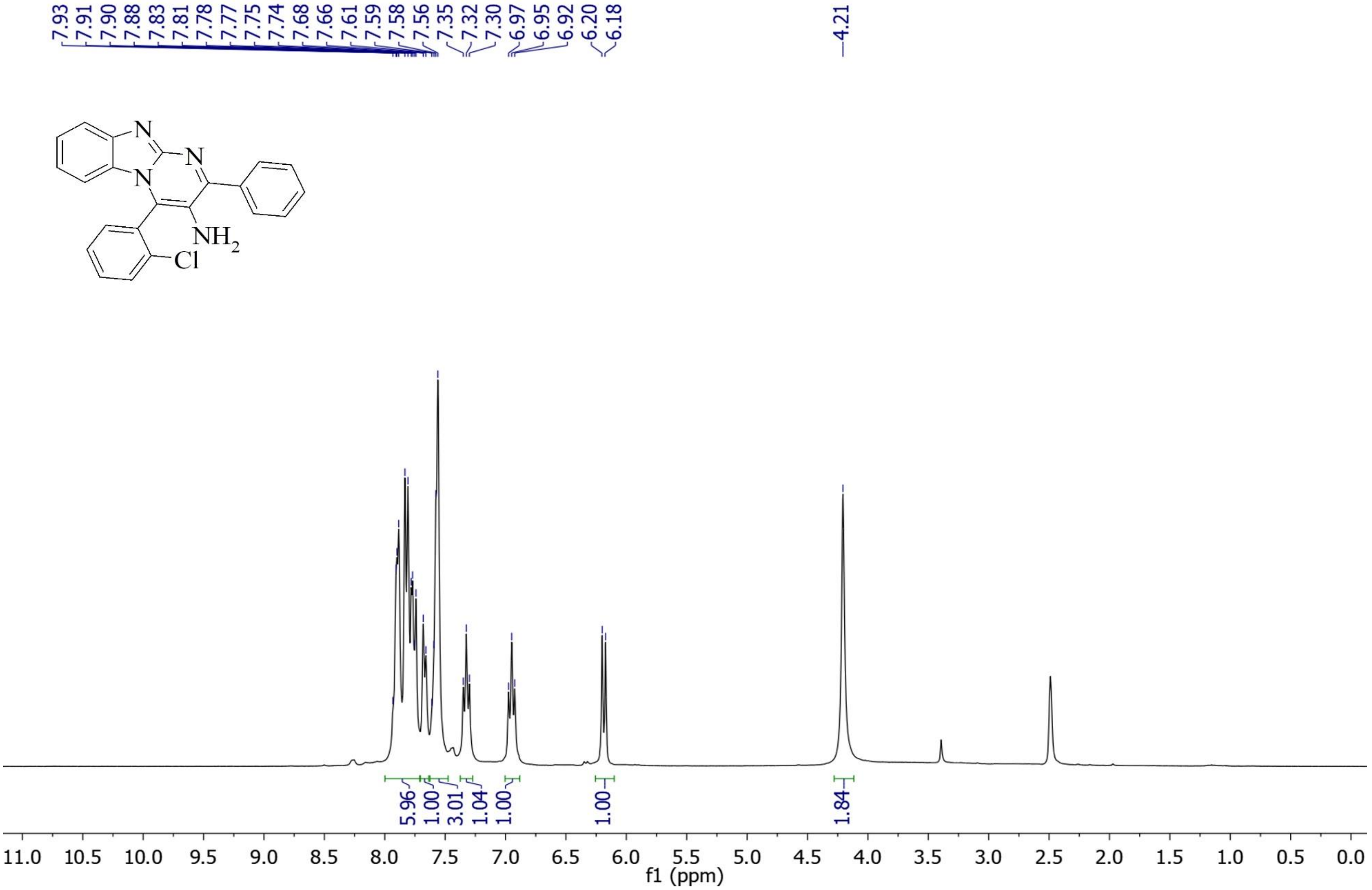

^{13}C NMR spectrum of 4-(2-chloro-phenyl)-2-phenyl-benzo[4,5]imidazo[1,2-a]pyrimidin-3-ylamine (**3e**)

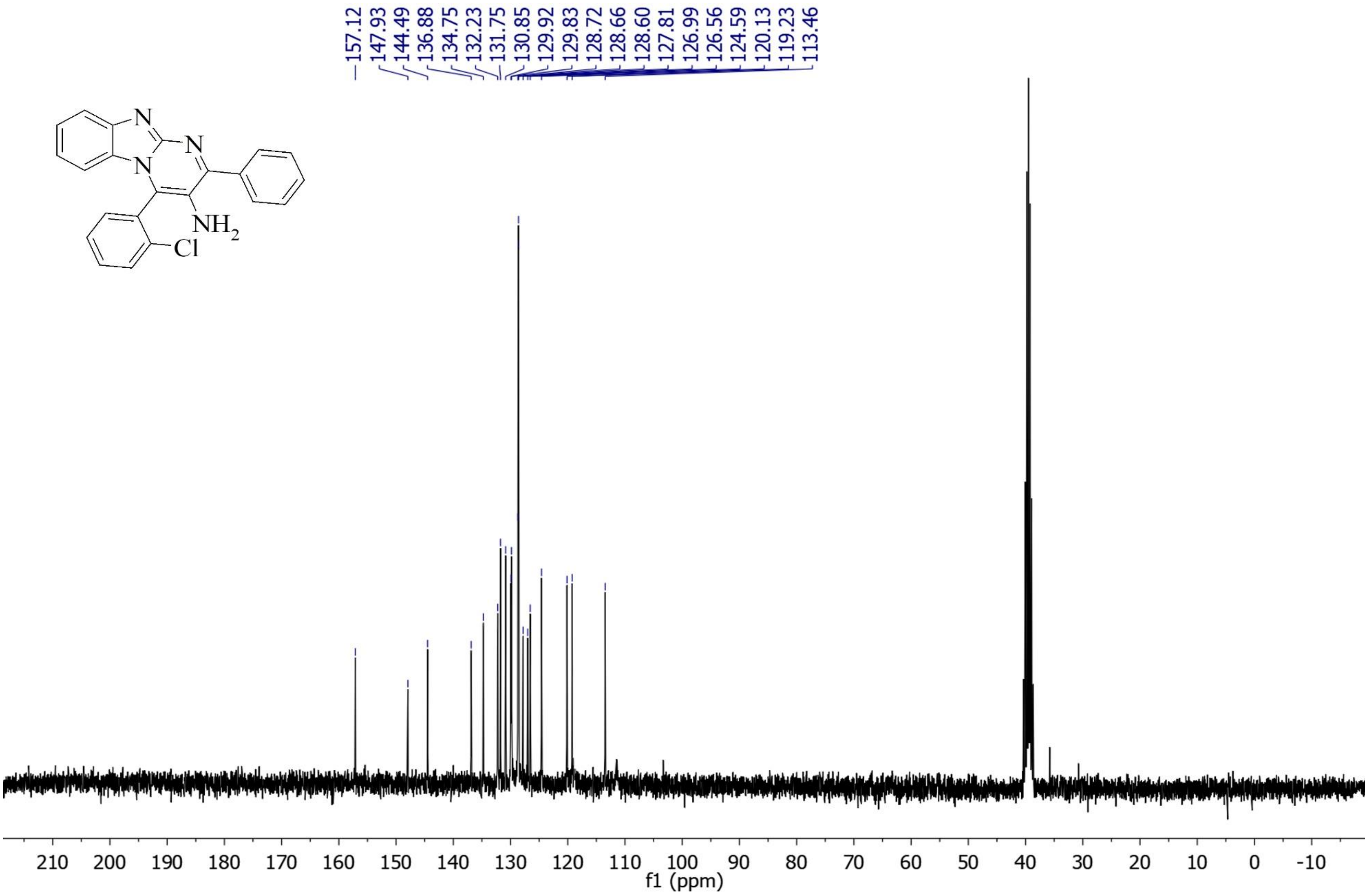

^{1}H NMR spectrum of 4-(3-chloro-phenyl)-2-phenyl-benzo[4,5]imidazo[1,2-a]pyrimidin-3-ylamine (**3f**)

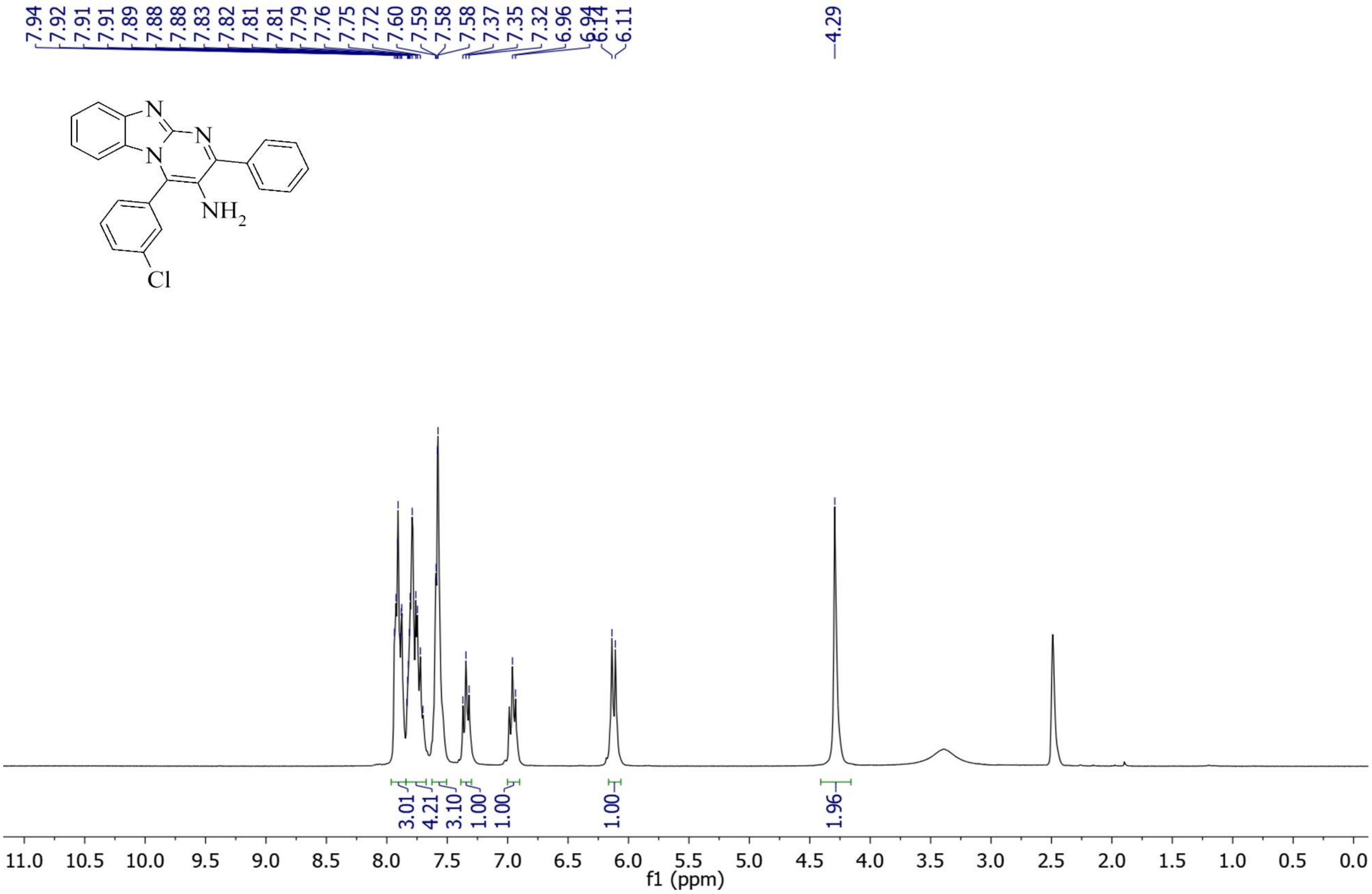

^{13}C NMR spectrum of 4-(3-chloro-phenyl)-2-phenyl-benzo[4,5]imidazo[1,2-a]pyrimidin-3-ylamine (**3f**)

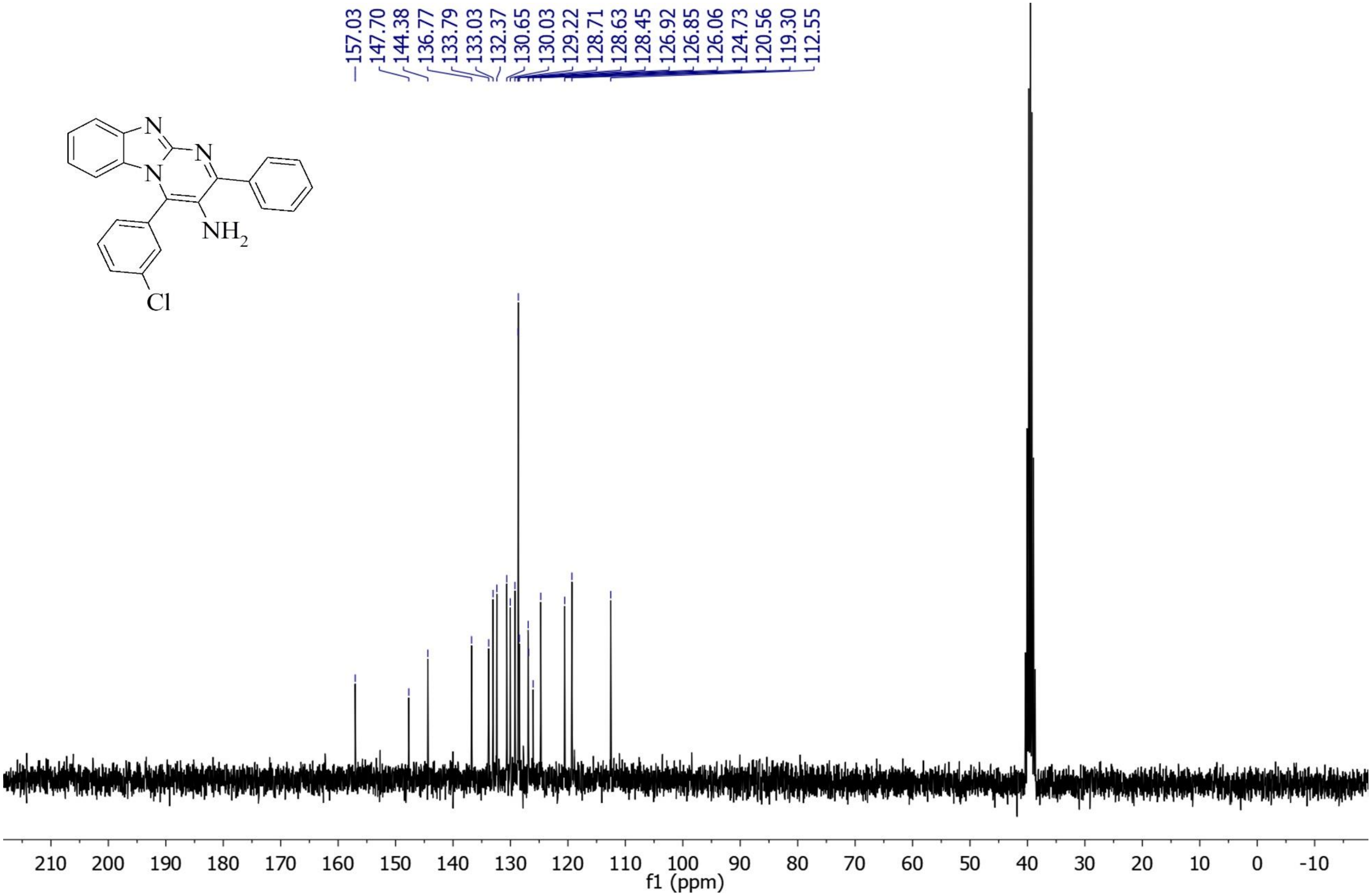

^{1}H NMR spectrum of 2-phenyl-4-thiophen-2-yl-benzo[4,5]imidazo[1,2-a]pyrimidin-3-ylamine (**3g**)

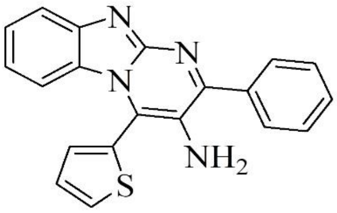

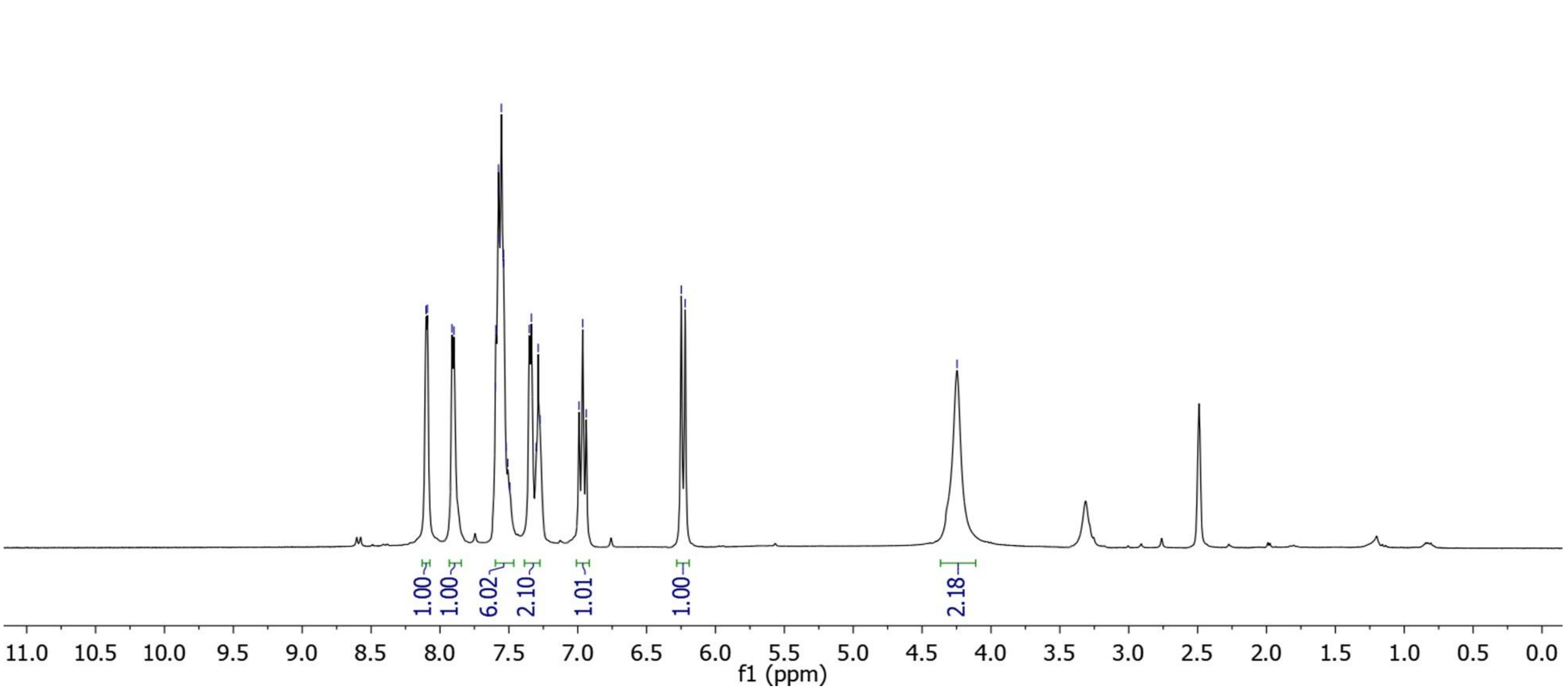

^{13}C NMR spectrum of 2-phenyl-4-thiophen-2-yl-benzo[4,5]imidazo[1,2-a]pyrimidin-3-ylamine (**3g**)

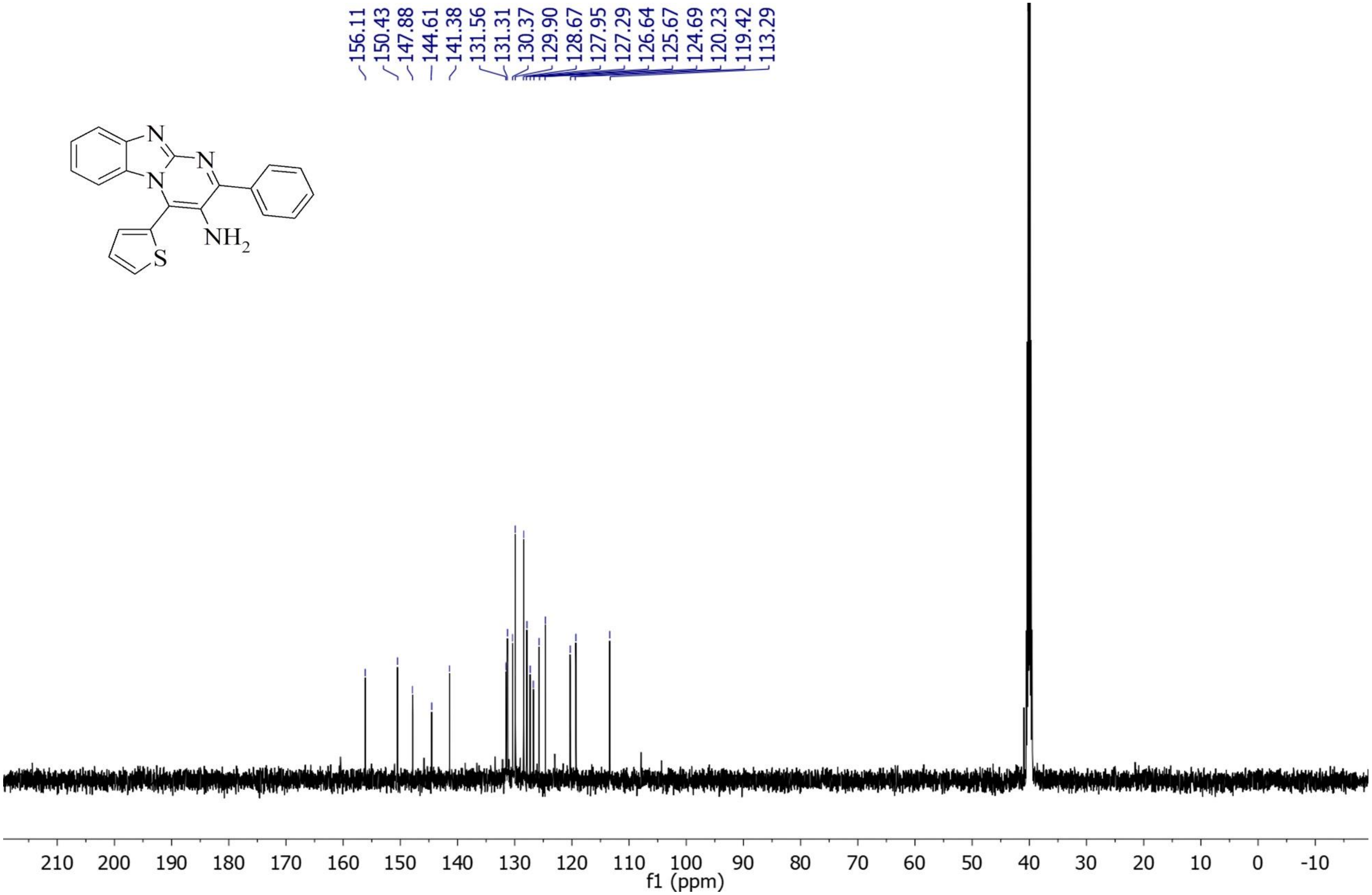

^{1}H NMR spectrum of 2-(4-chloro-phenyl)-4-phenyl-benzo[4,5]imidazo[1,2-a]pyrimidin-3-ylamine (**3h**)

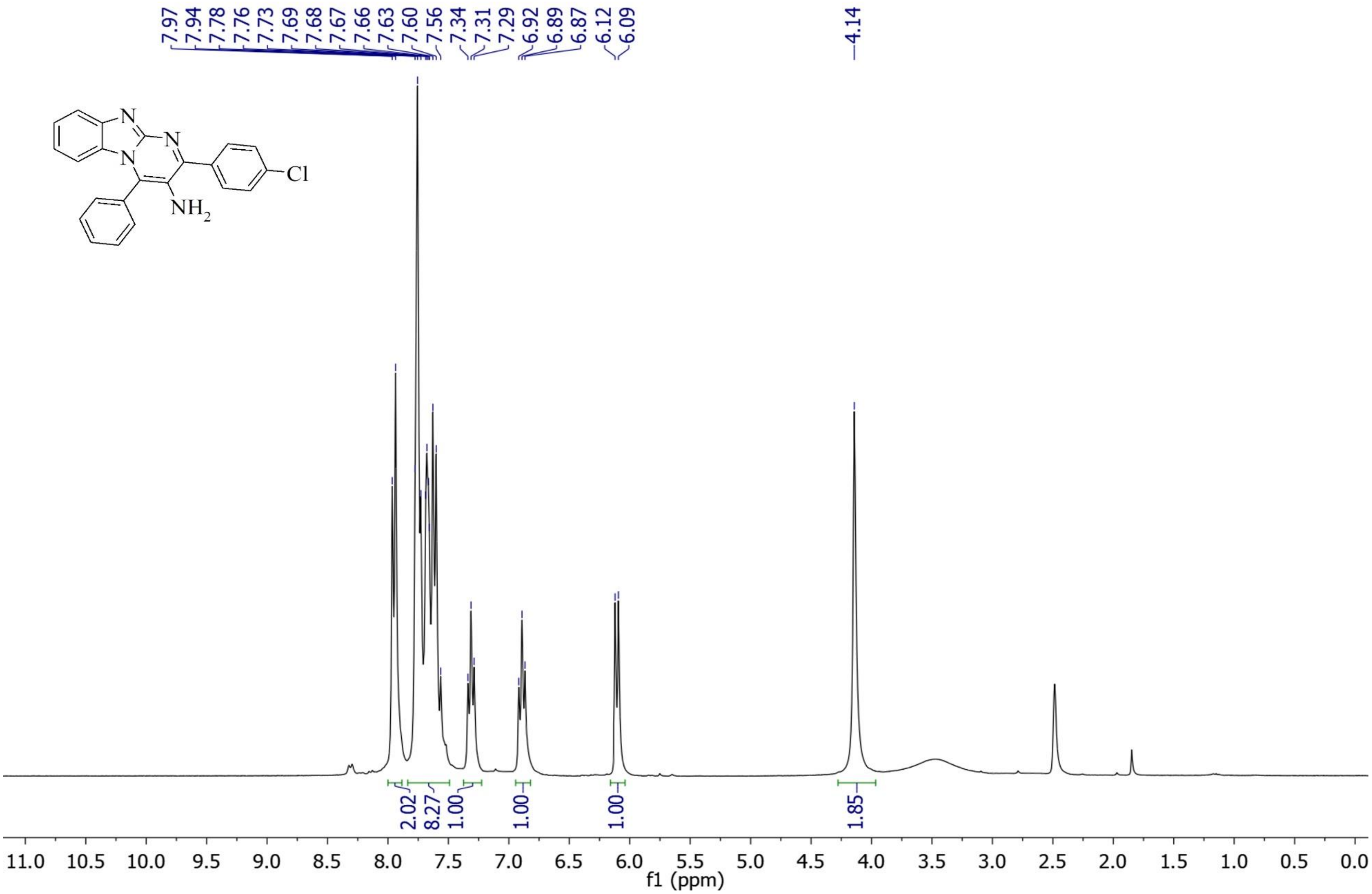

^{13}C NMR spectrum of 2-(4-chloro-phenyl)-4-phenyl-benzo[4,5]imidazo[1,2-a]pyrimidin-3-ylamine (**3h**)

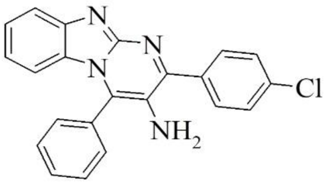

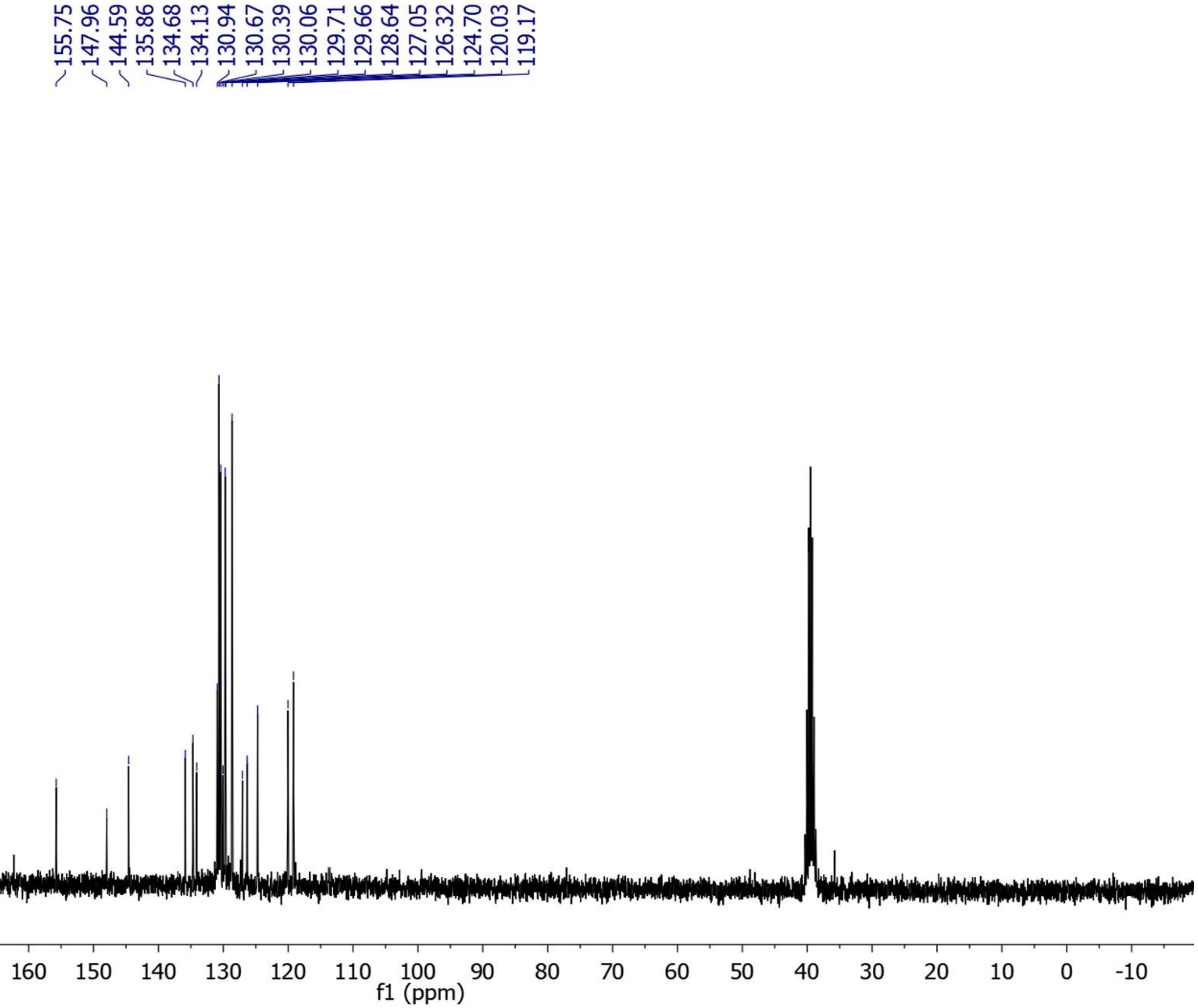

^{1}H NMR spectrum of 2-(4-chloro-phenyl)-4-p-tolyl-benzo[4,5]imidazo[1,2-a]pyrimidin-3-ylamine (**3i**)

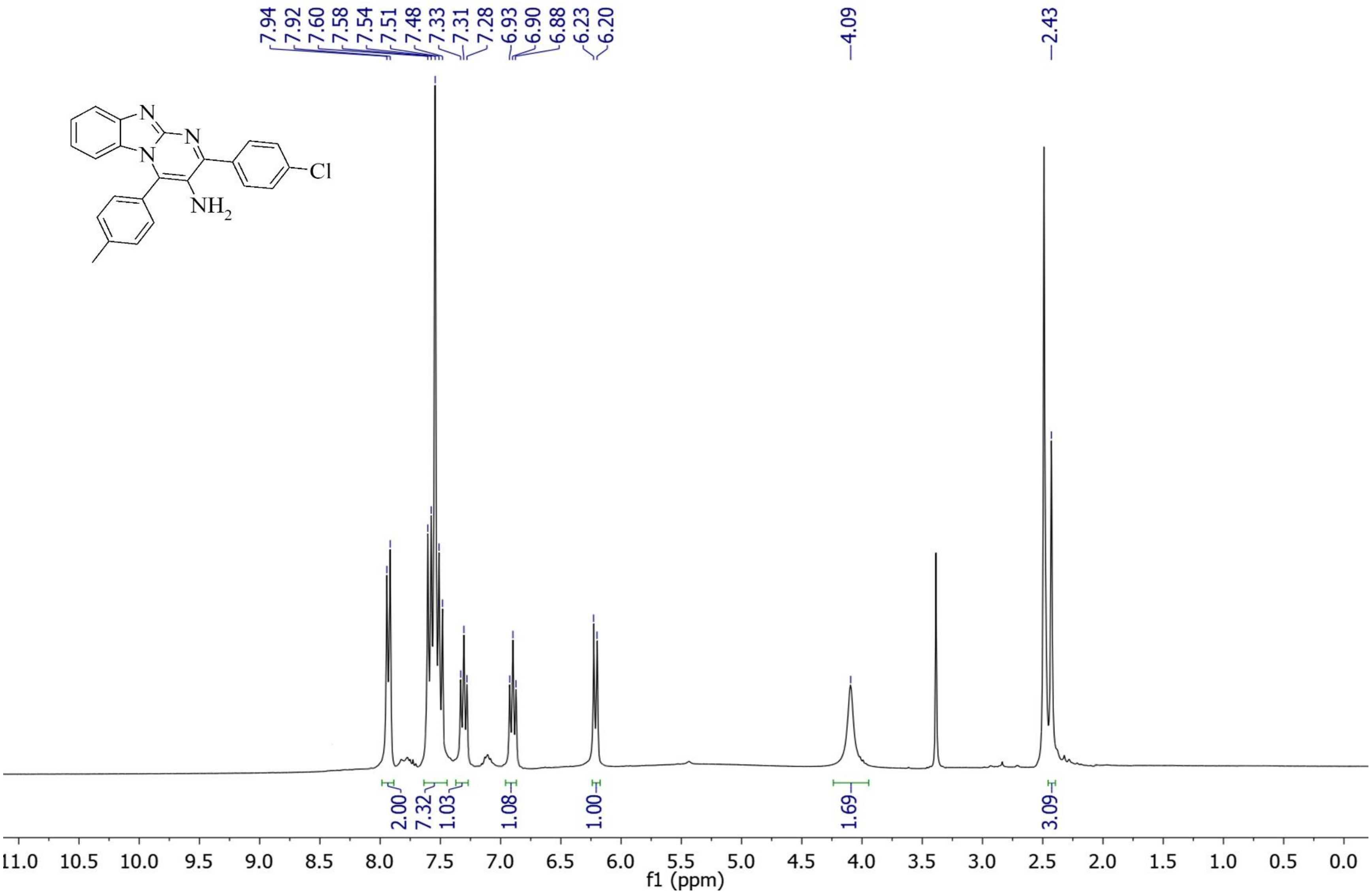

^{13}C NMR spectrum of 2-(4-chloro-phenyl)-4-p-tolyl-benzo[4,5]imidazo[1,2-a]pyrimidin-3-ylamine (**3i**)

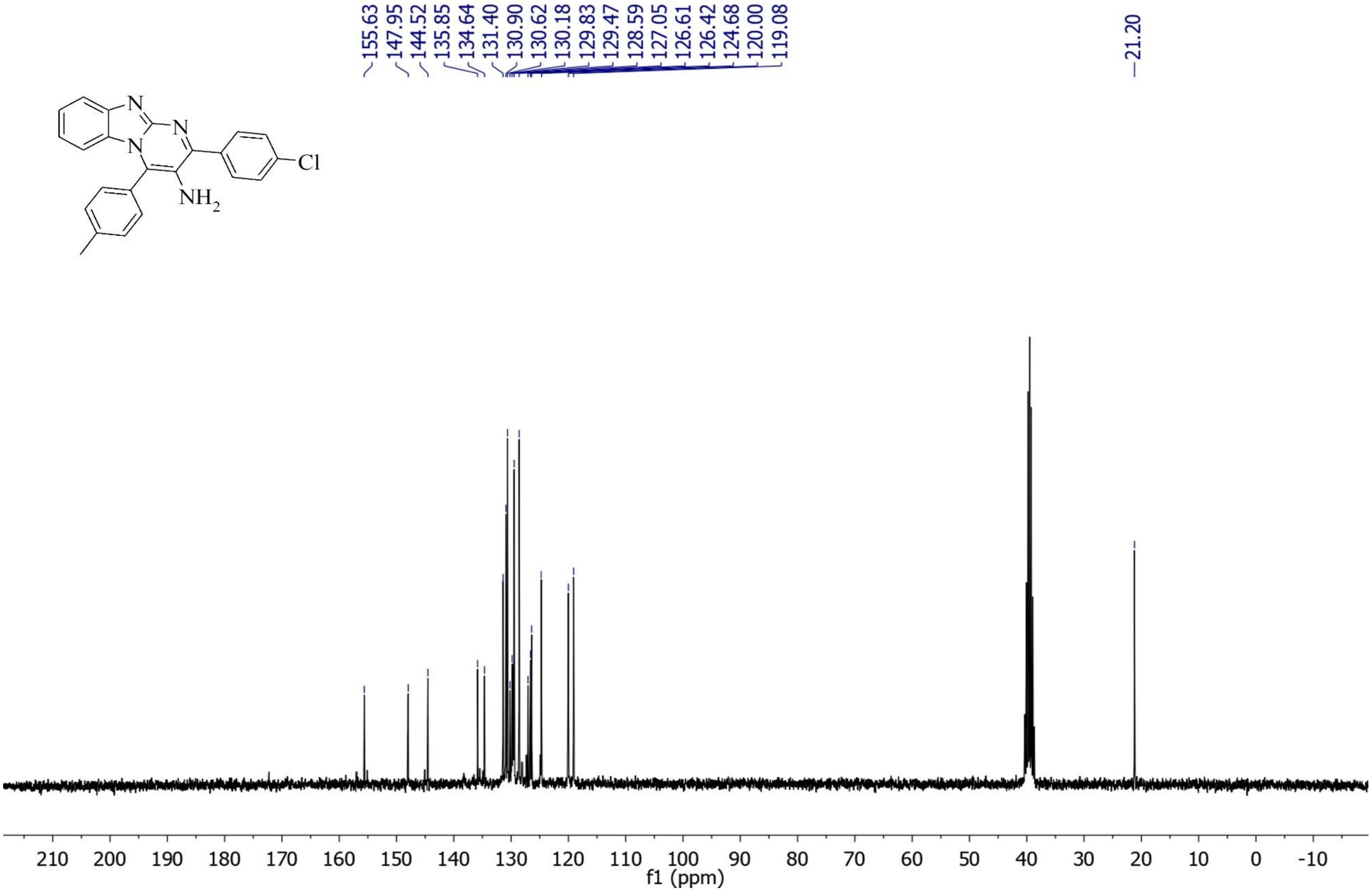

^{1}H NMR spectrum of 2-(4-chloro-phenyl)-4-(4-methoxy-phenyl)-benzo[4,5]imidazo[1,2-a]pyrimidin-3-ylamine (**3j**)

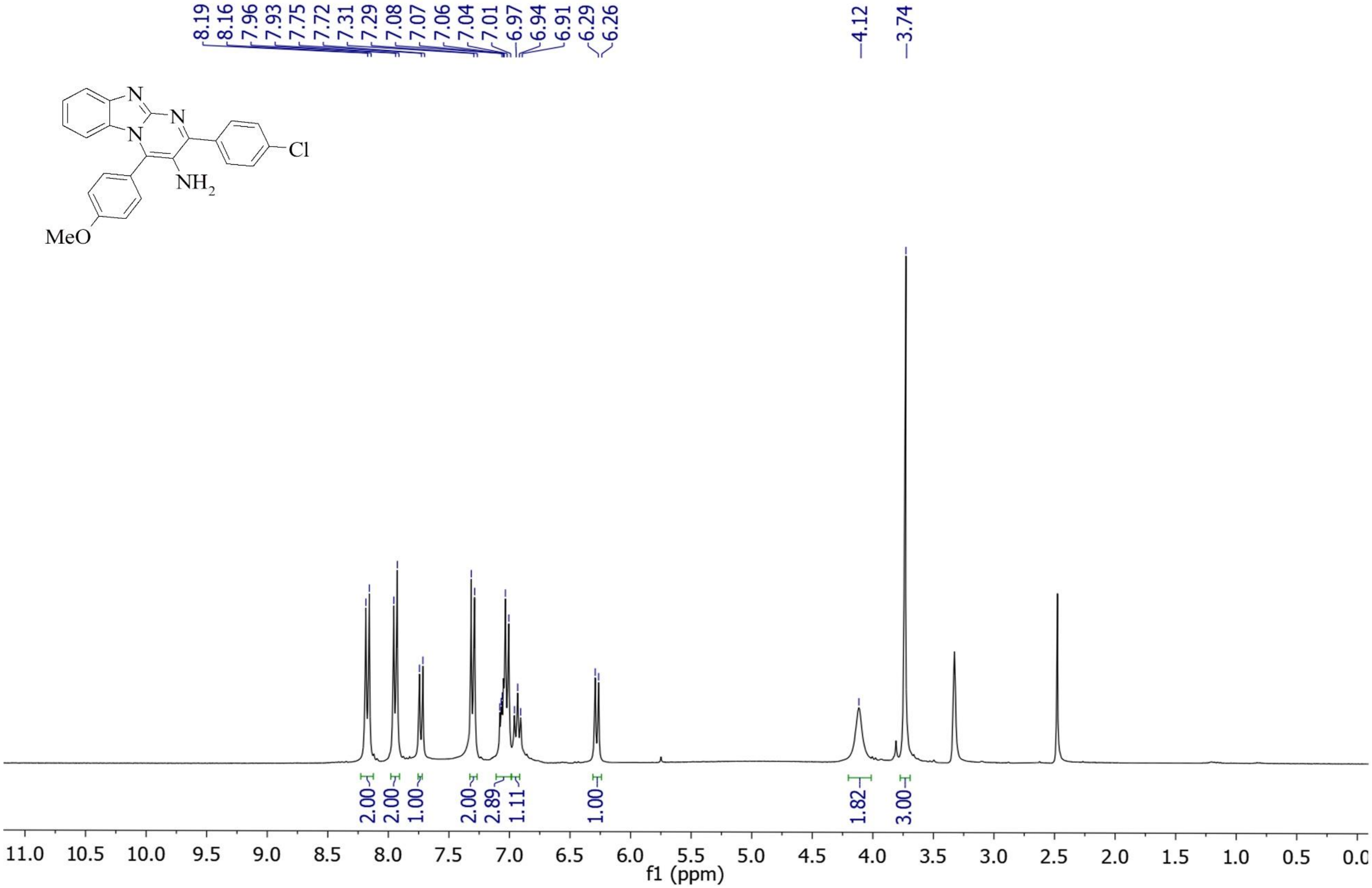

^{13}C NMR spectrum of 2-(4-chloro-phenyl)-4-(4-methoxy-phenyl)-benzo[4,5]imidazo[1,2-a]pyrimidin-3-ylamine (**3j**)

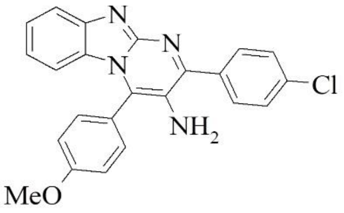

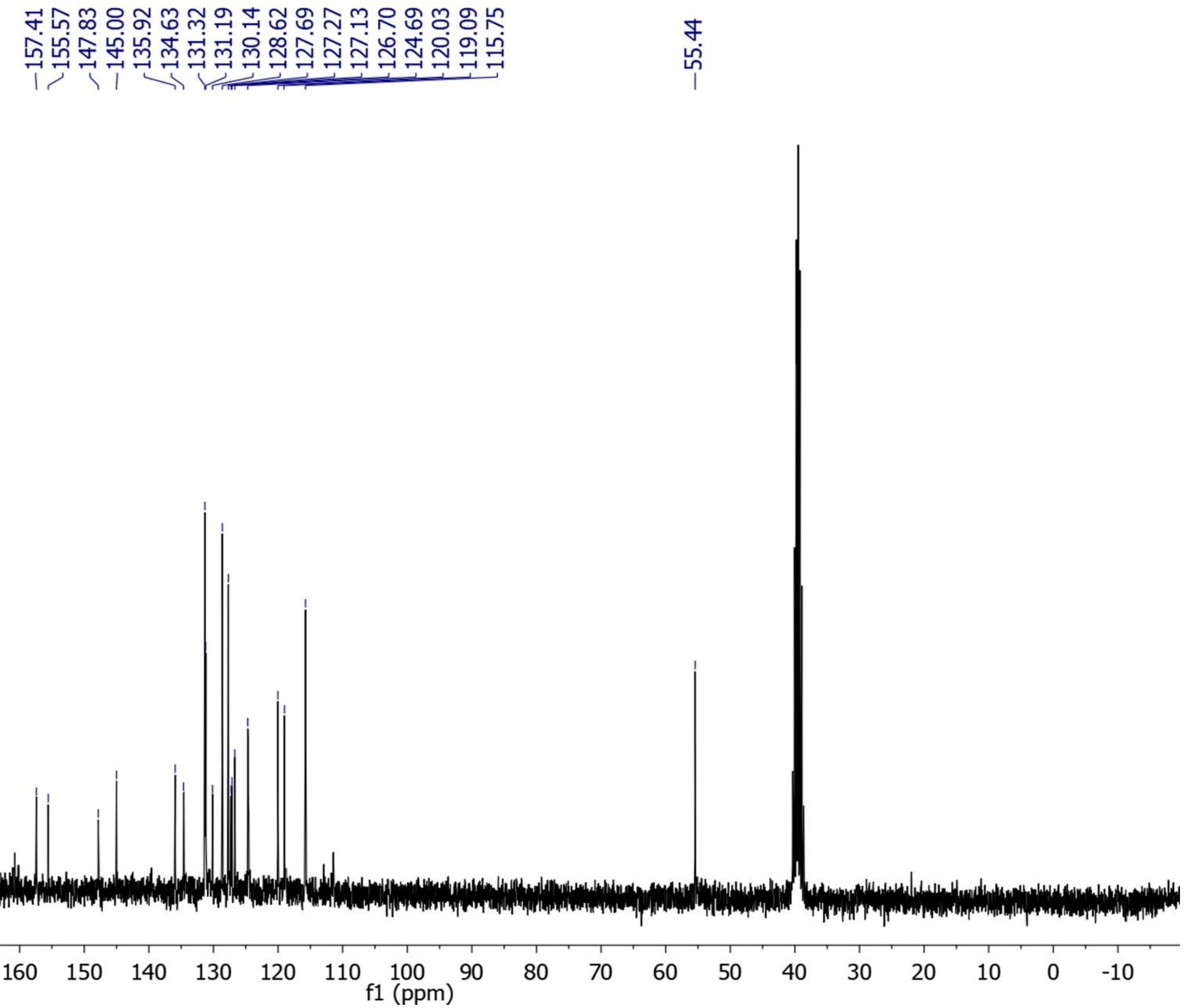

^{1}H NMR spectrum of 2,4-bis-(4-chloro-phenyl)-benzo[4,5]imidazo[1,2-a]pyrimidin-3-ylamine (**3k**)

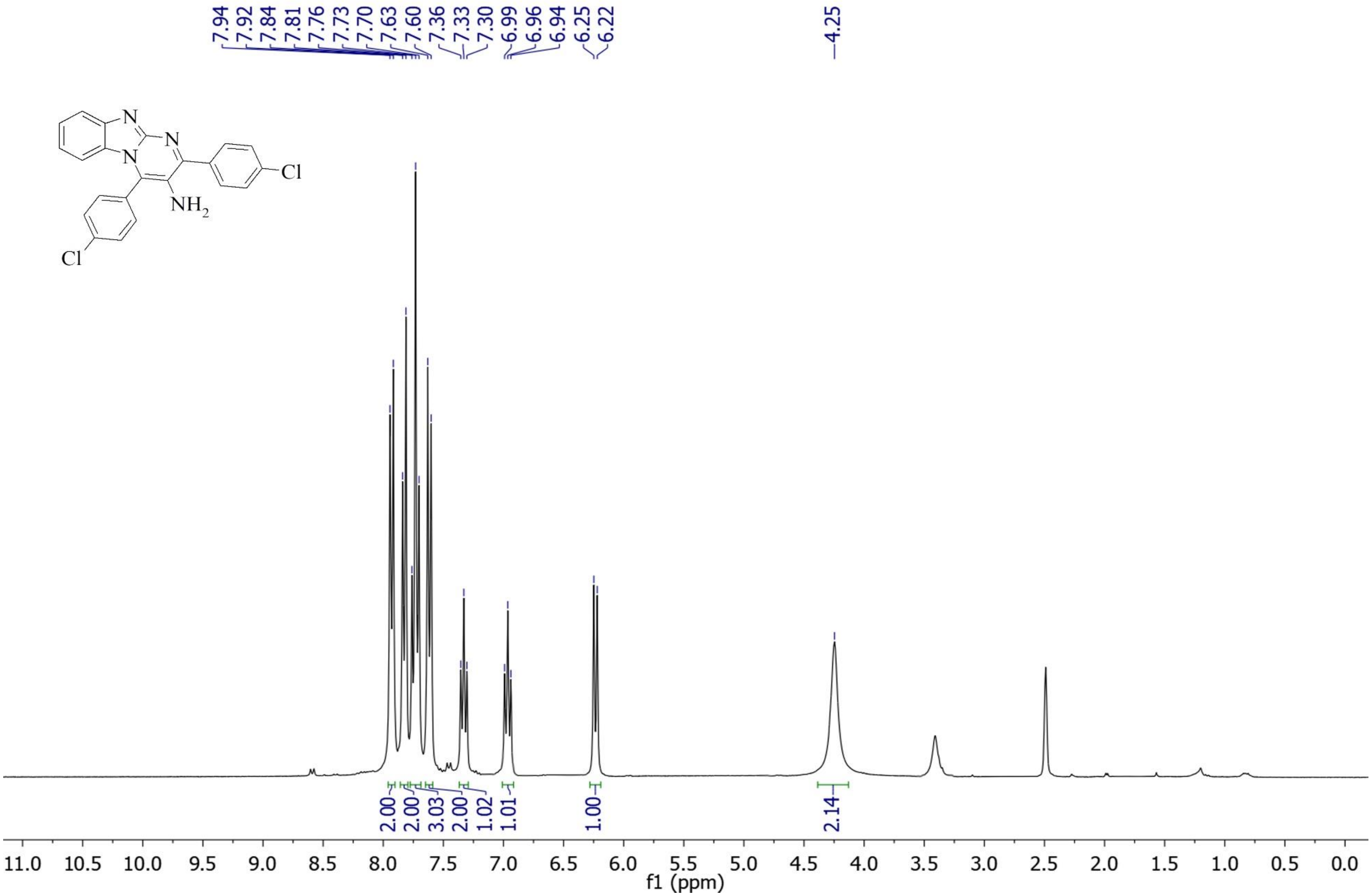

^{13}C NMR spectrum of 2,4-bis-(4-chloro-phenyl)-benzo[4,5]imidazo[1,2-a]pyrimidin-3-ylamine (**3k**)

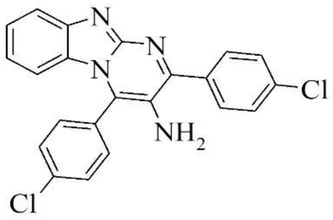

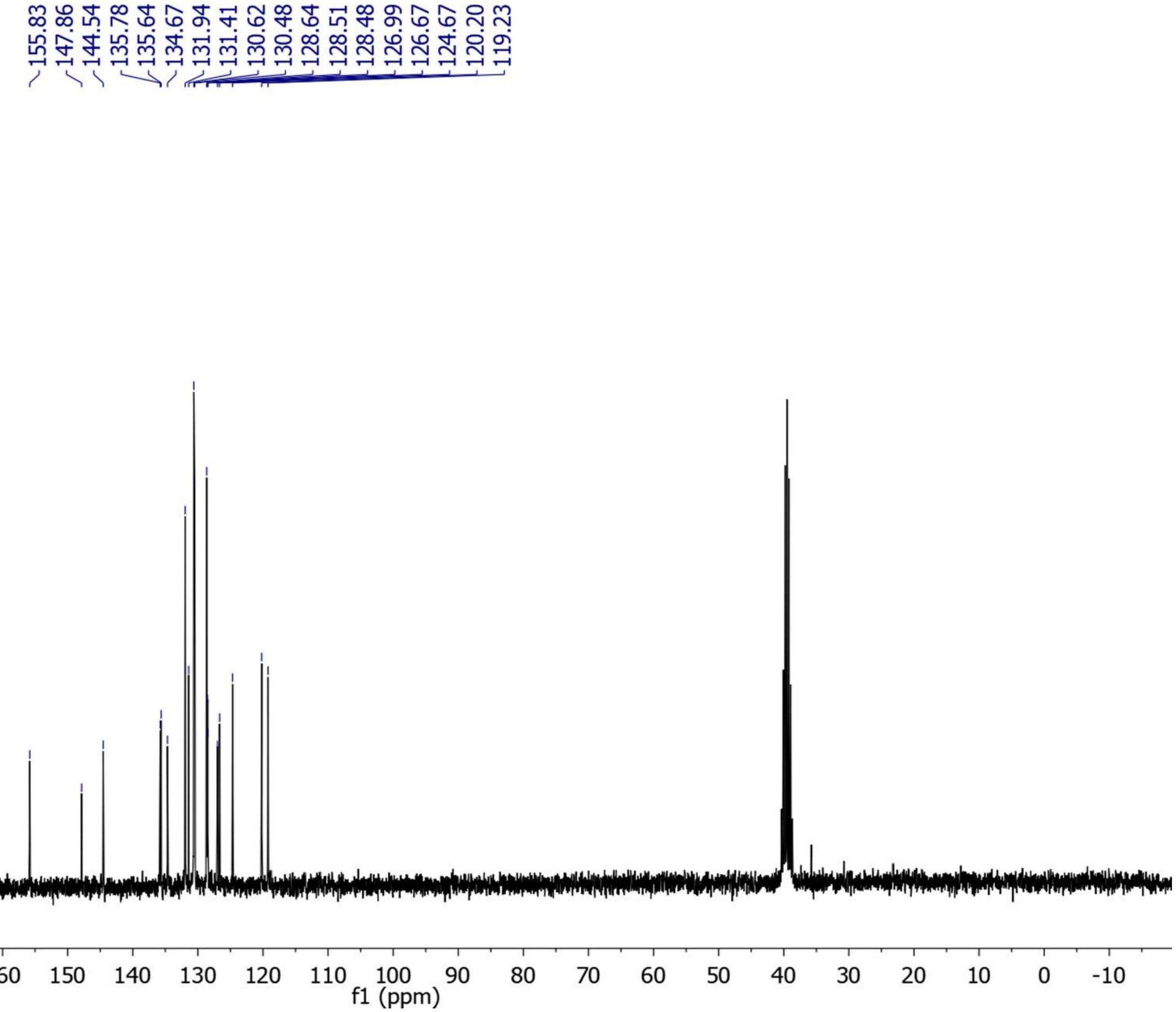

^{1}H NMR spectrum of 3-(4-chloro-phenyl)-1-thiophen-2-yl-benzo[4,5]imidazo[1,2-a]pyridin-2-ylamine (**3l**)

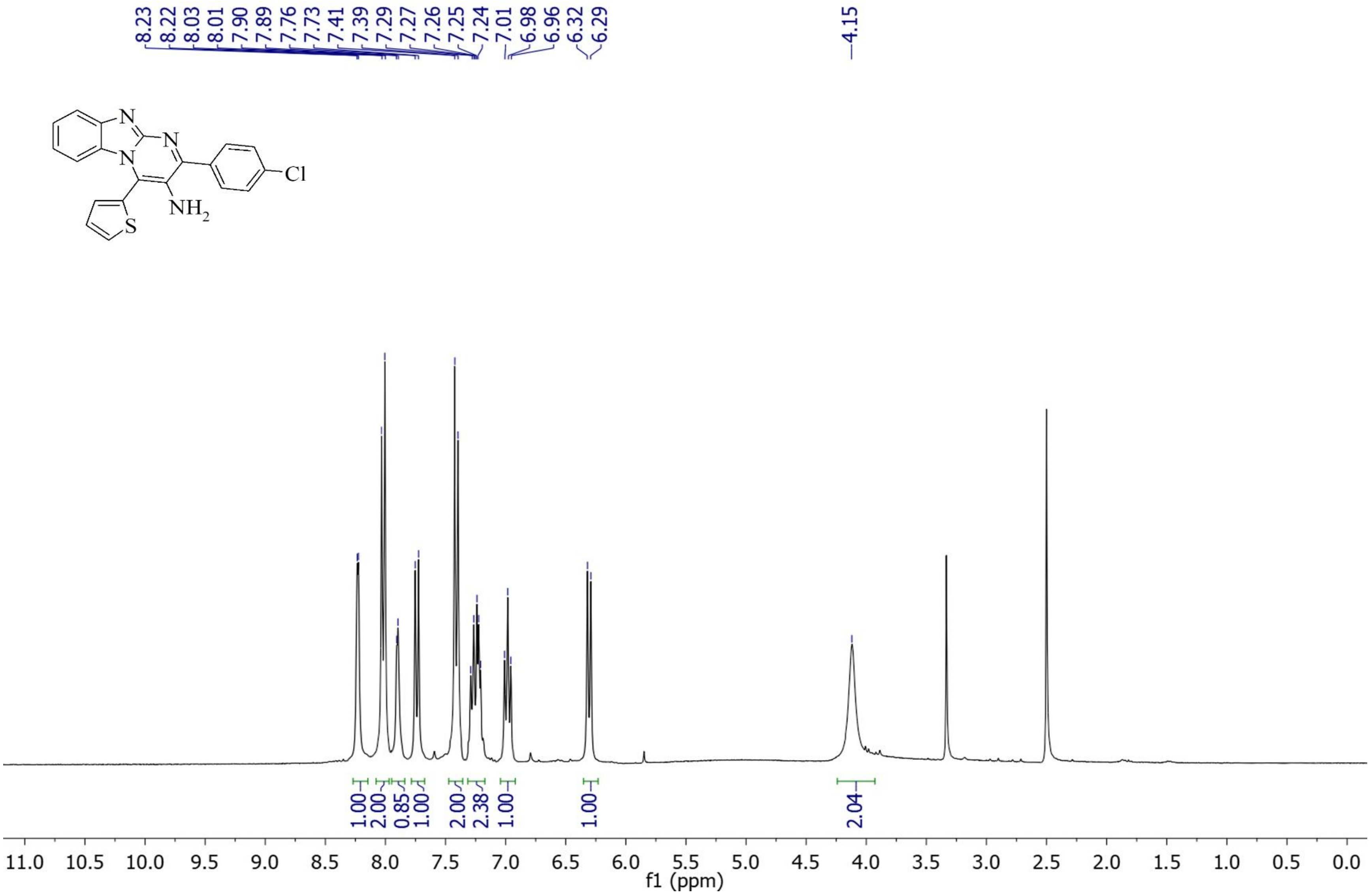

^{13}C NMR spectrum of 3-(4-chloro-phenyl)-1-thiophen-2-yl-benzo[4,5]imidazo[1,2-a]pyridin-2-ylamine (**3l**)

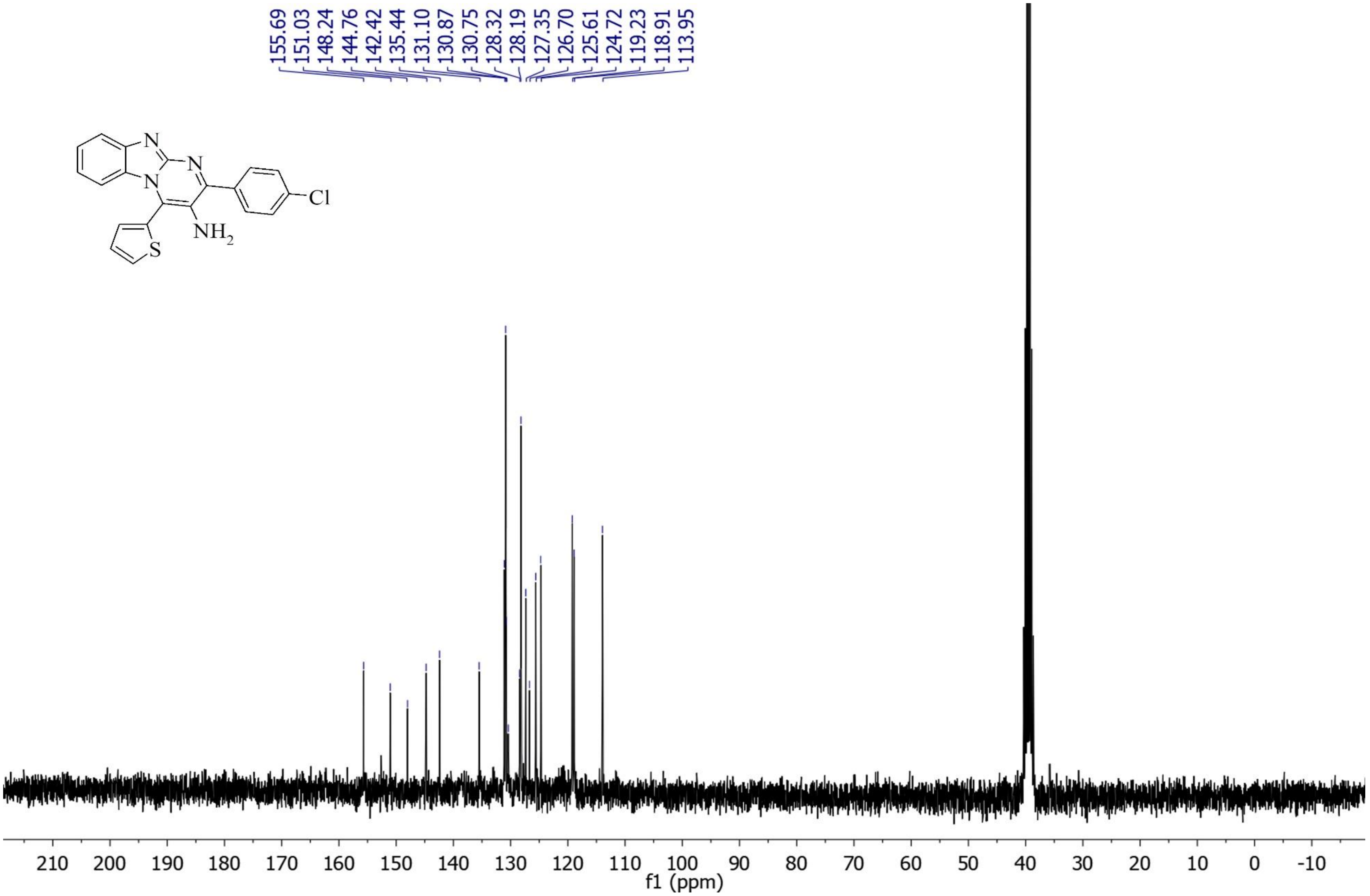

^{1}H NMR spectrum of 2-(4-bromo-phenyl)-4-phenyl-benzo[4,5]imidazo[1,2-a]pyrimidin-3-ylamine (**3m**)

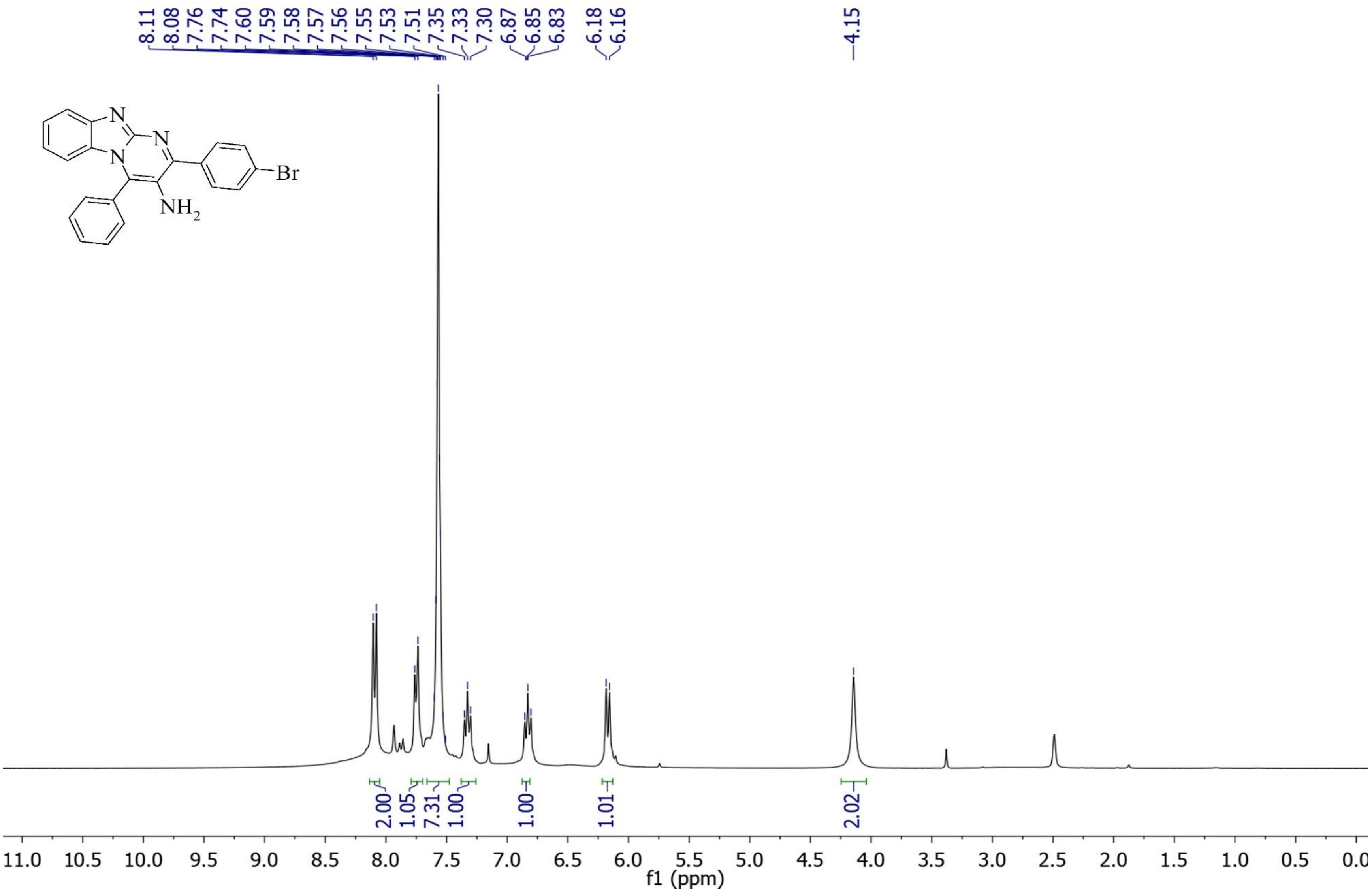

^{13}C NMR spectrum of 2-(4-bromo-phenyl)-4-phenyl-benzo[4,5]imidazo[1,2-a]pyrimidin-3-ylamine (**3m**)

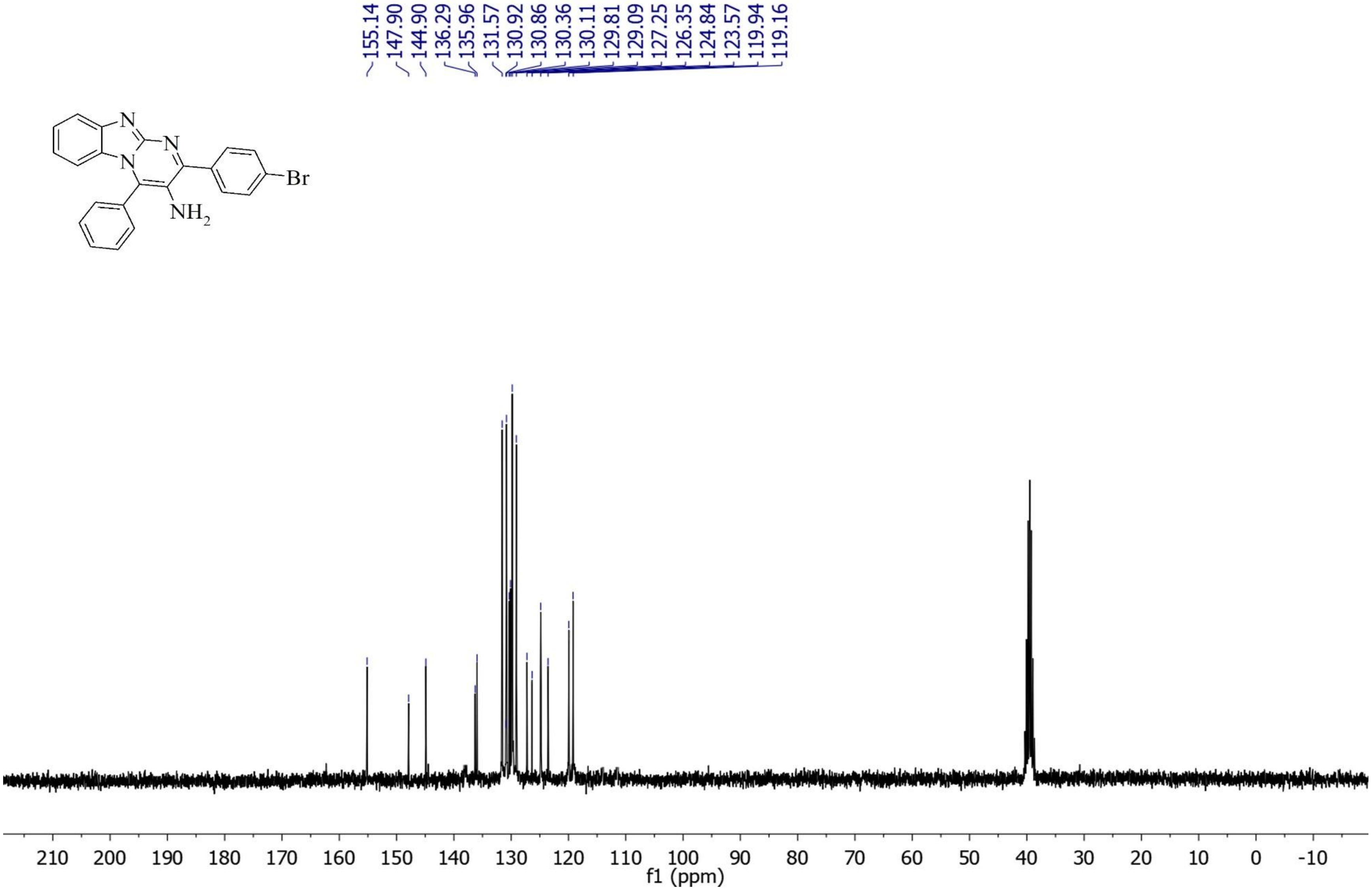

^{1}H NMR spectrum of 2-(4-bromo-phenyl)-4-p-tolyl-benzo[4,5]imidazo[1,2-a]pyrimidin-3-ylamine (**3n**)

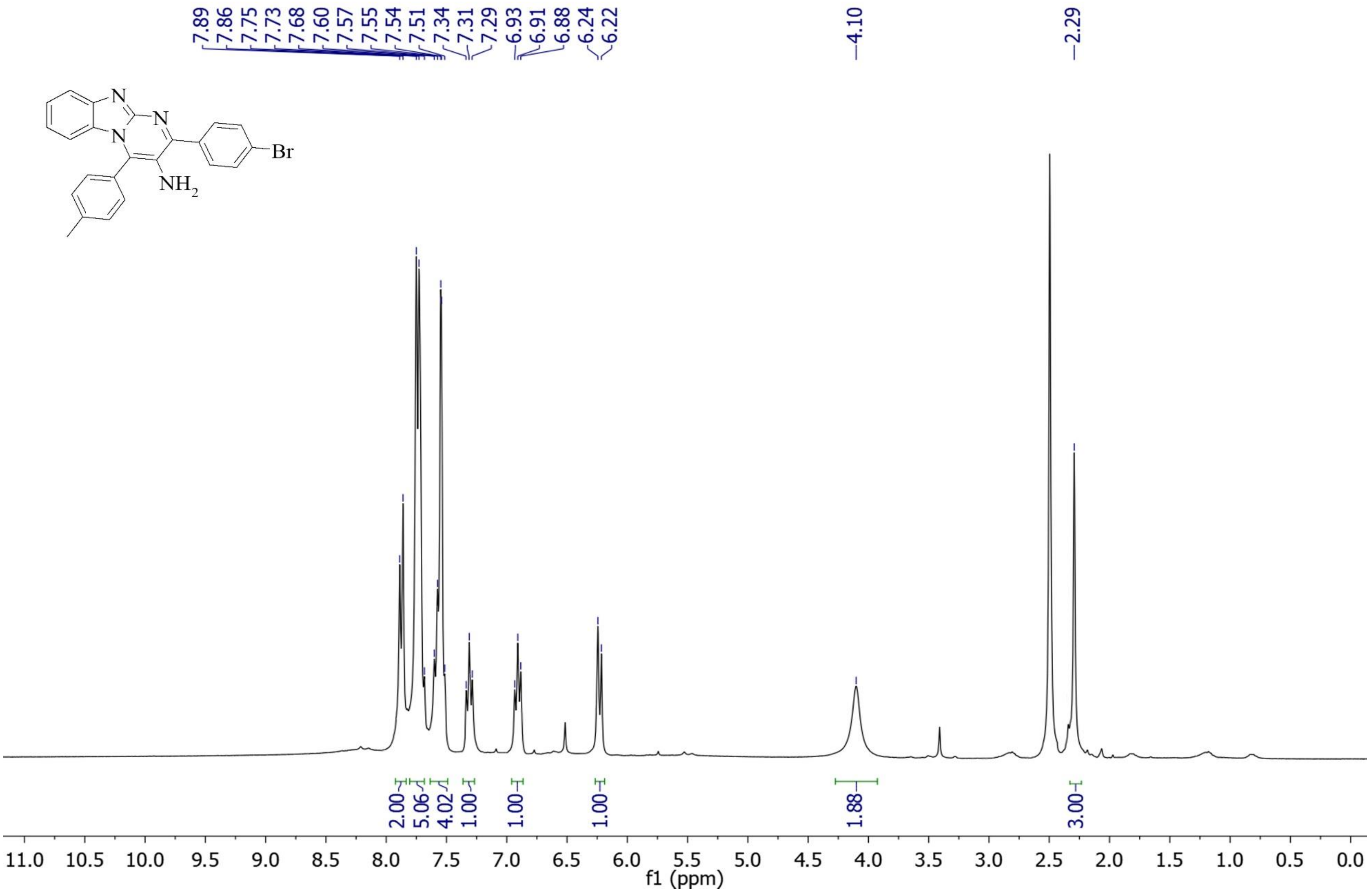

^{13}C NMR spectrum of 2-(4-bromo-phenyl)-4-p-tolyl-benzo[4,5]imidazo[1,2-a]pyrimidin-3-ylamine (**3n**)

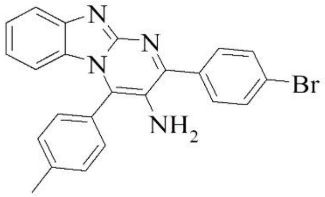

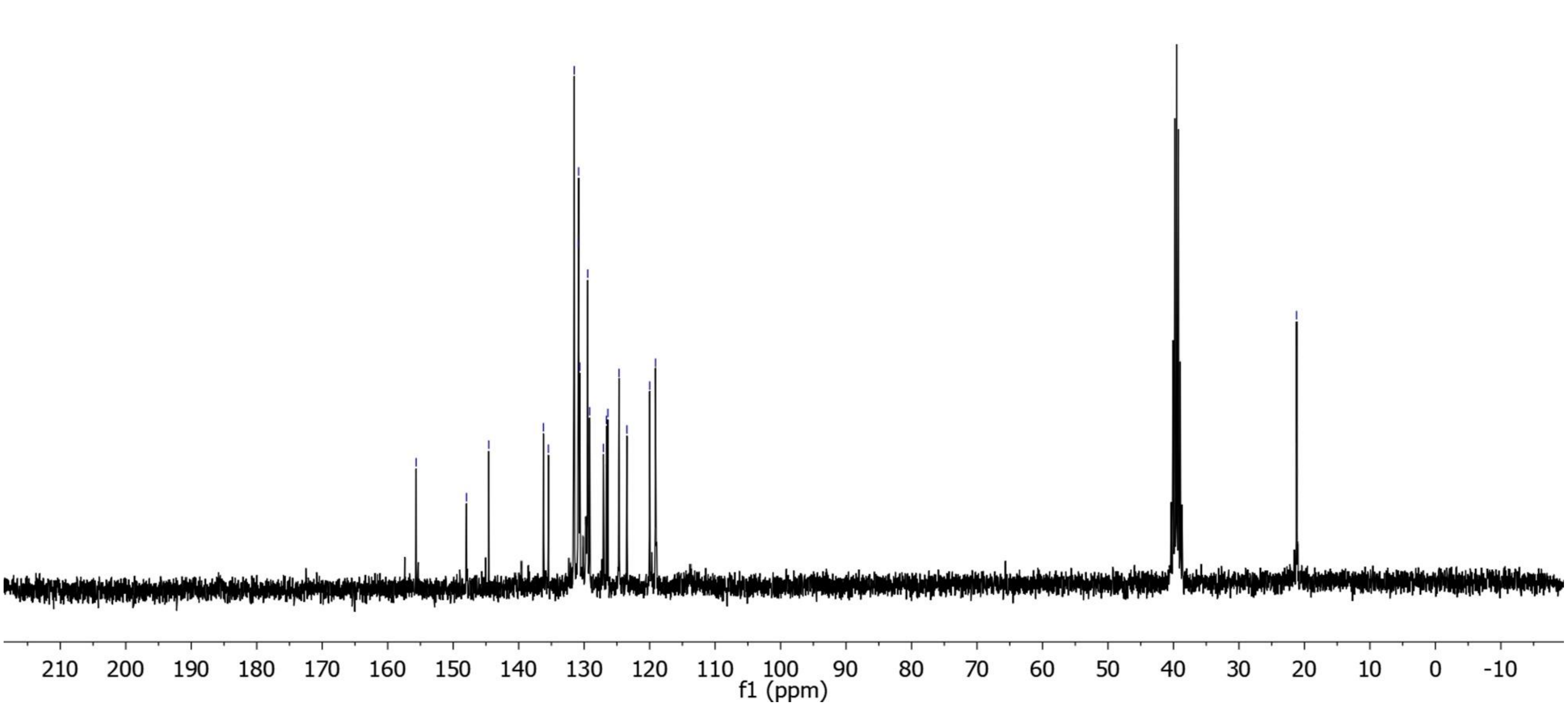

^{1}H NMR spectrum of 2-(4-methoxy-phenyl)-4-phenyl-benzo[4,5]imidazo[1,2-a]pyrimidin-3-ylamine (**3o**)

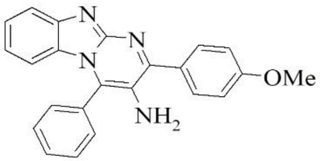

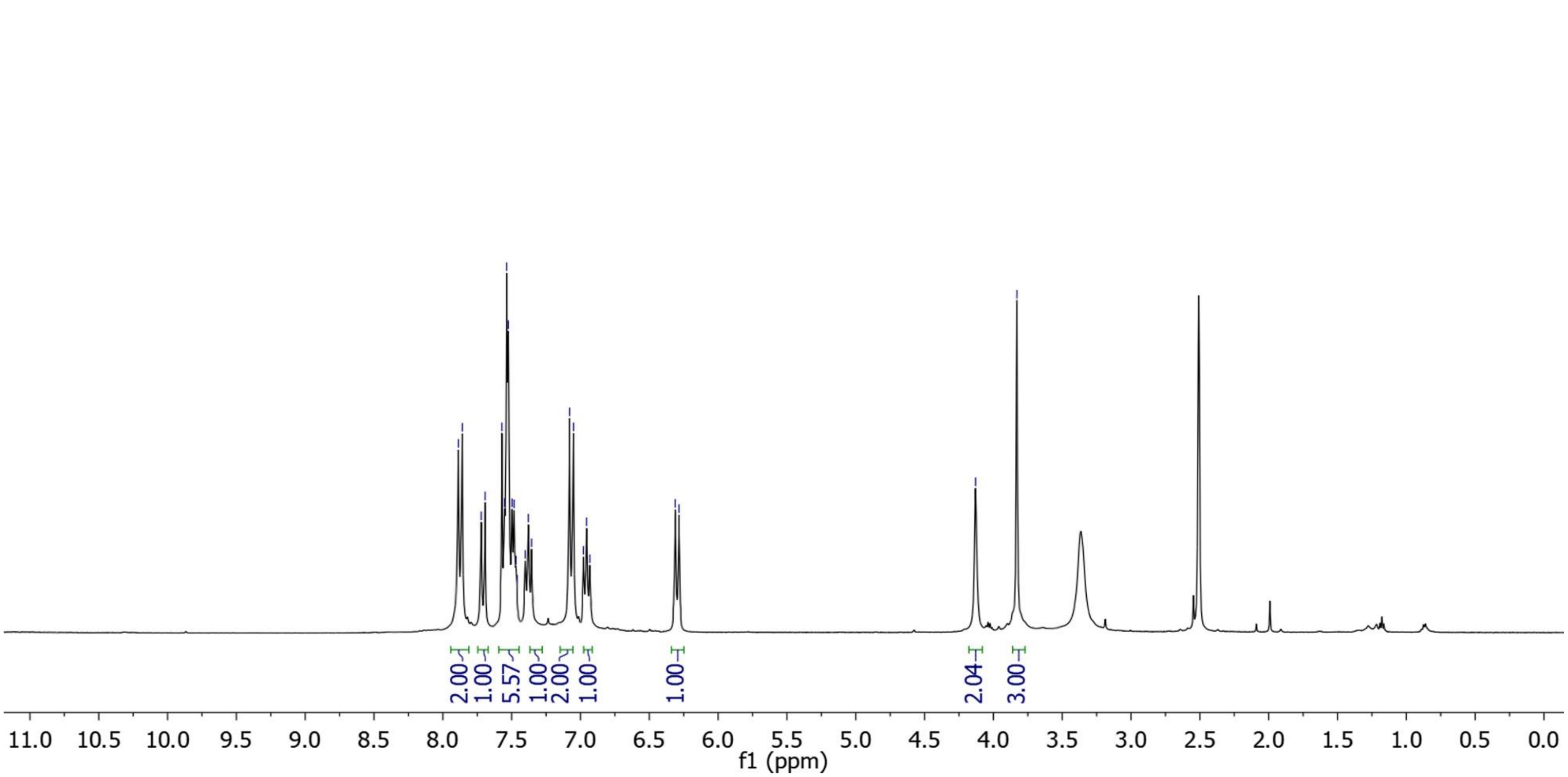

^{13}C NMR spectrum of 2-(4-methoxy-phenyl)-4-phenyl-benzo[4,5]imidazo[1,2-a]pyrimidin-3-ylamine (**3o**)

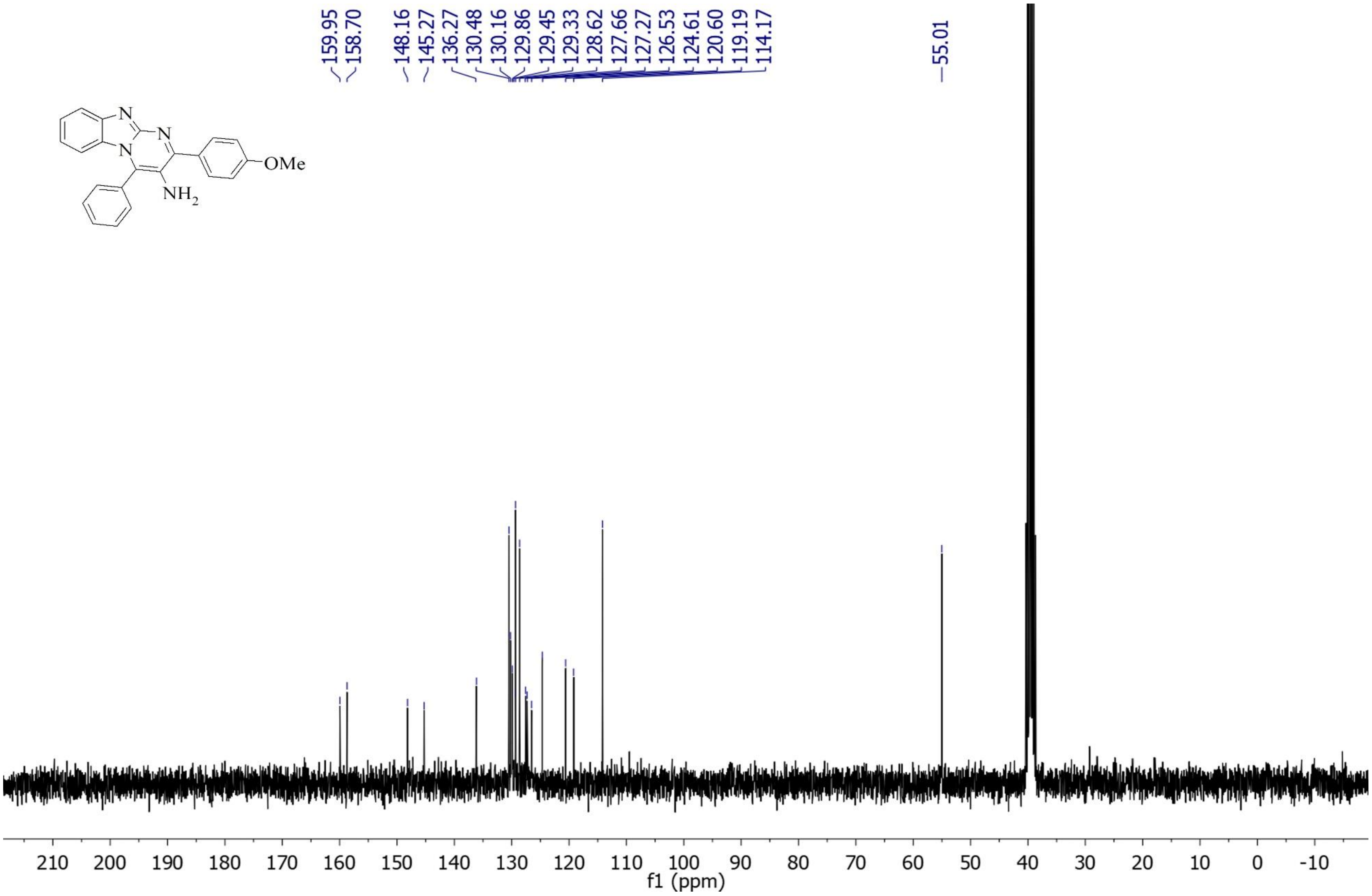

^{1}H NMR spectrum of 2-(4-methoxy-phenyl)-4-p-tolyl-benzo[4,5]imidazo[1,2-a]pyrimidin-3-ylamine (**3p**)

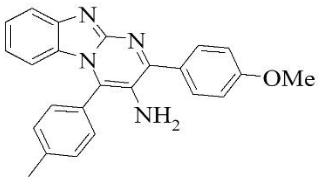

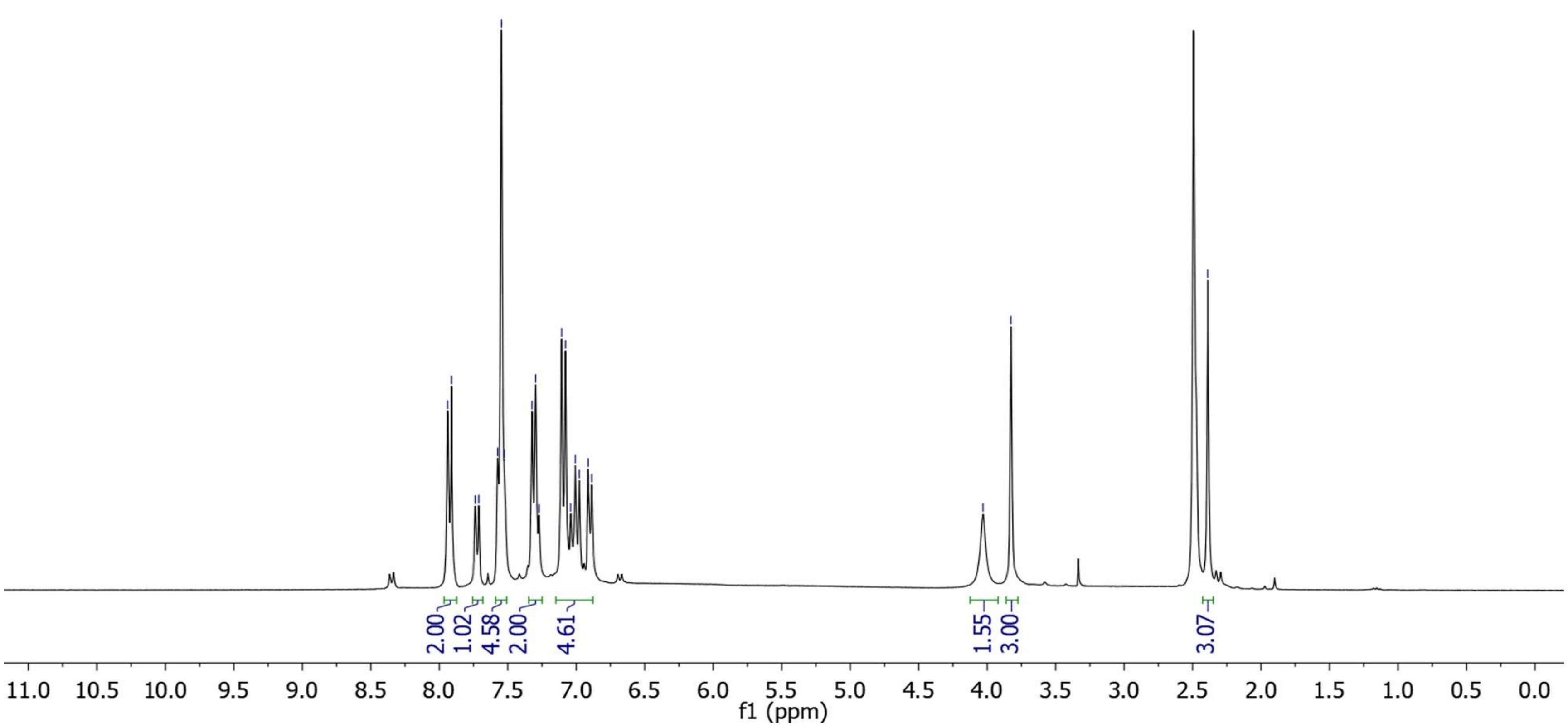

^{13}C NMR spectrum of 2-(4-methoxy-phenyl)-4-p-tolyl-benzo[4,5]imidazo[1,2-a]pyrimidin-3-ylamine (**3p**)

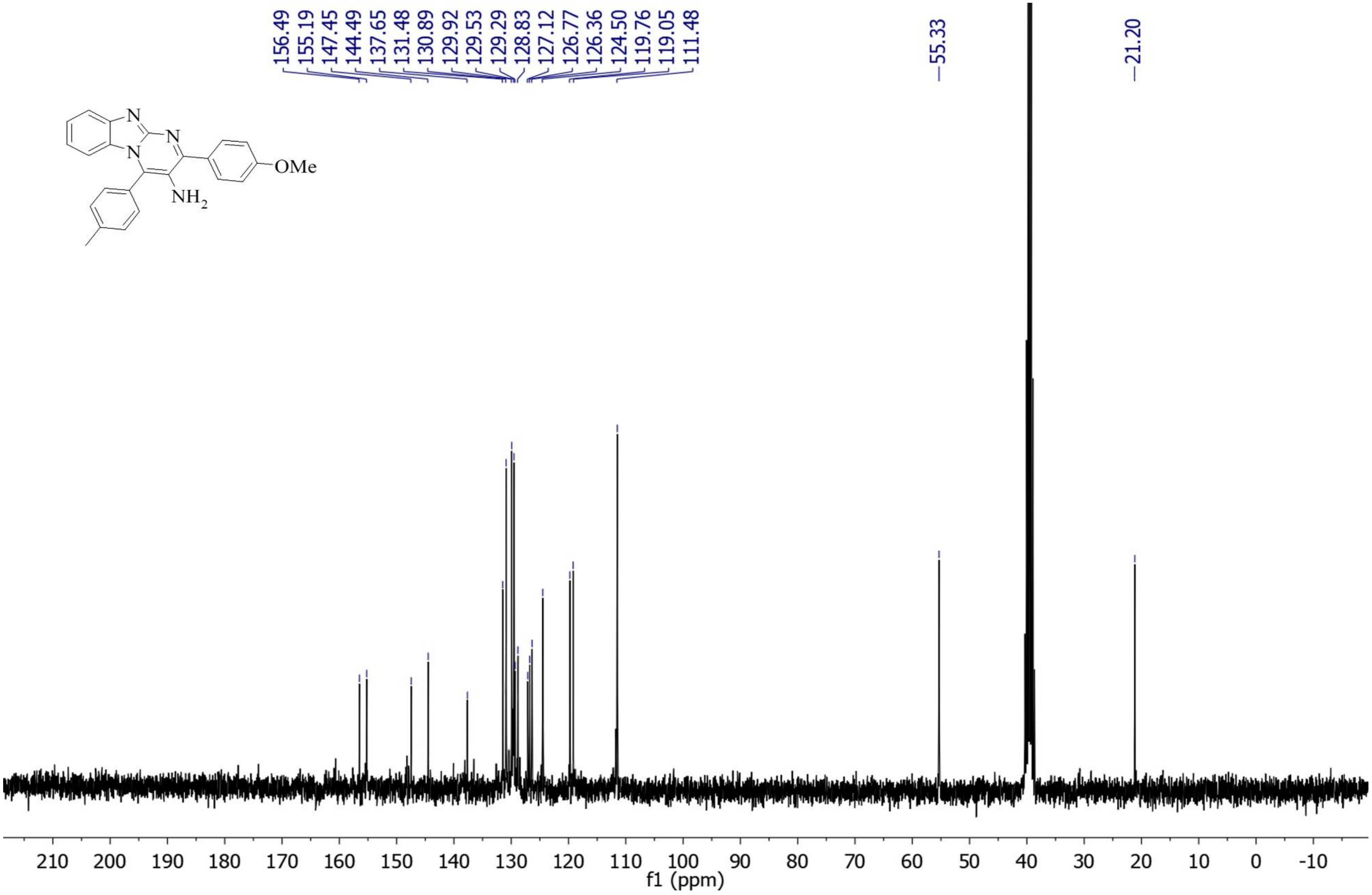

^{1}H NMR spectrum of 2,4-bis-(4-methoxy-phenyl)-benzo[4,5]imidazo[1,2-a]pyrimidin-3-ylamine (**3q**)

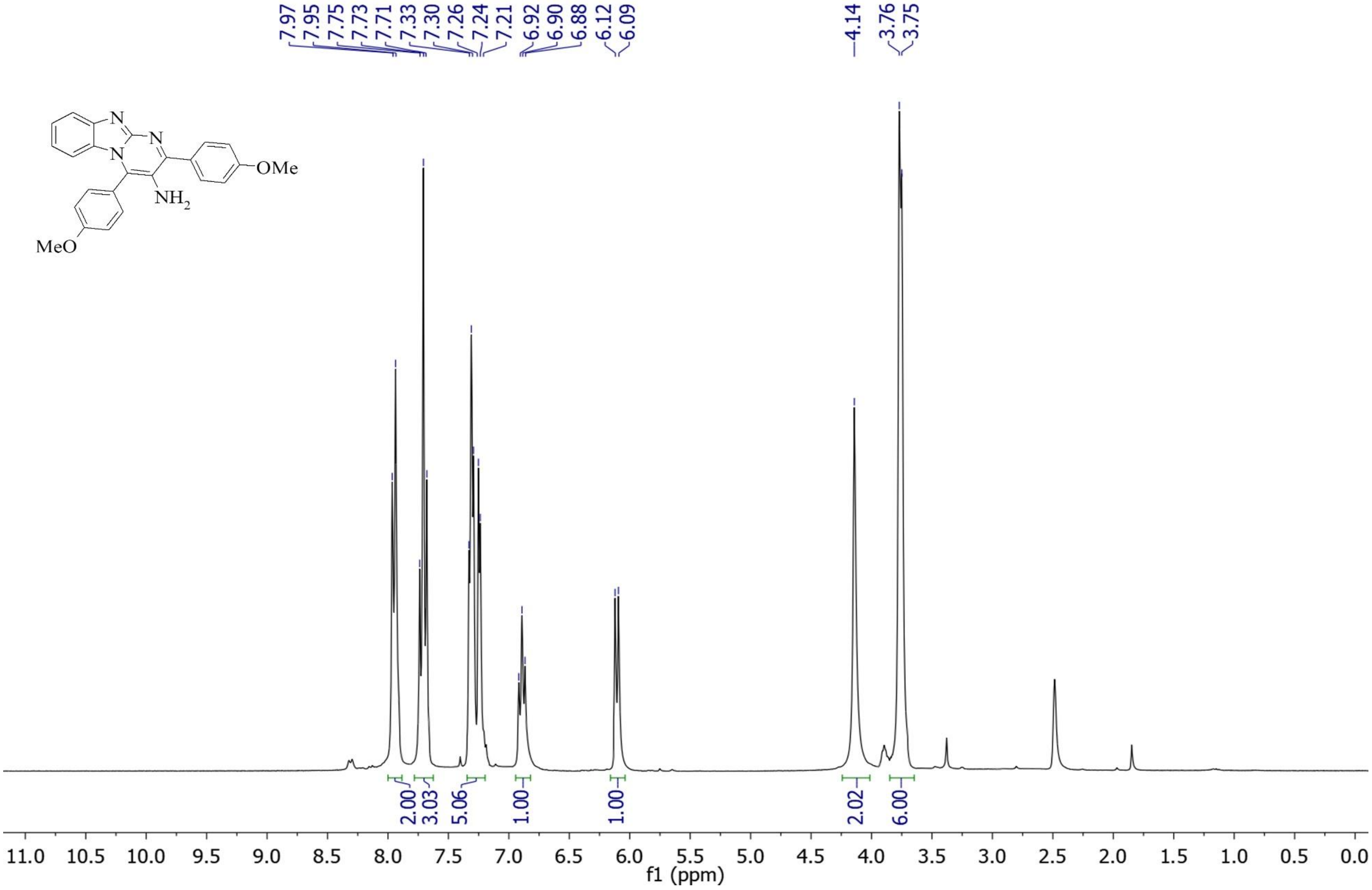

^{13}C NMR spectrum of 2,4-bis-(4-methoxy-phenyl)-benzo[4,5]imidazo[1,2-a]pyrimidin-3-ylamine (**3q**)

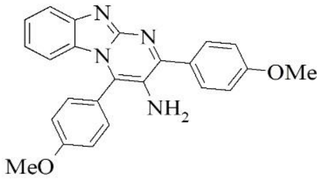

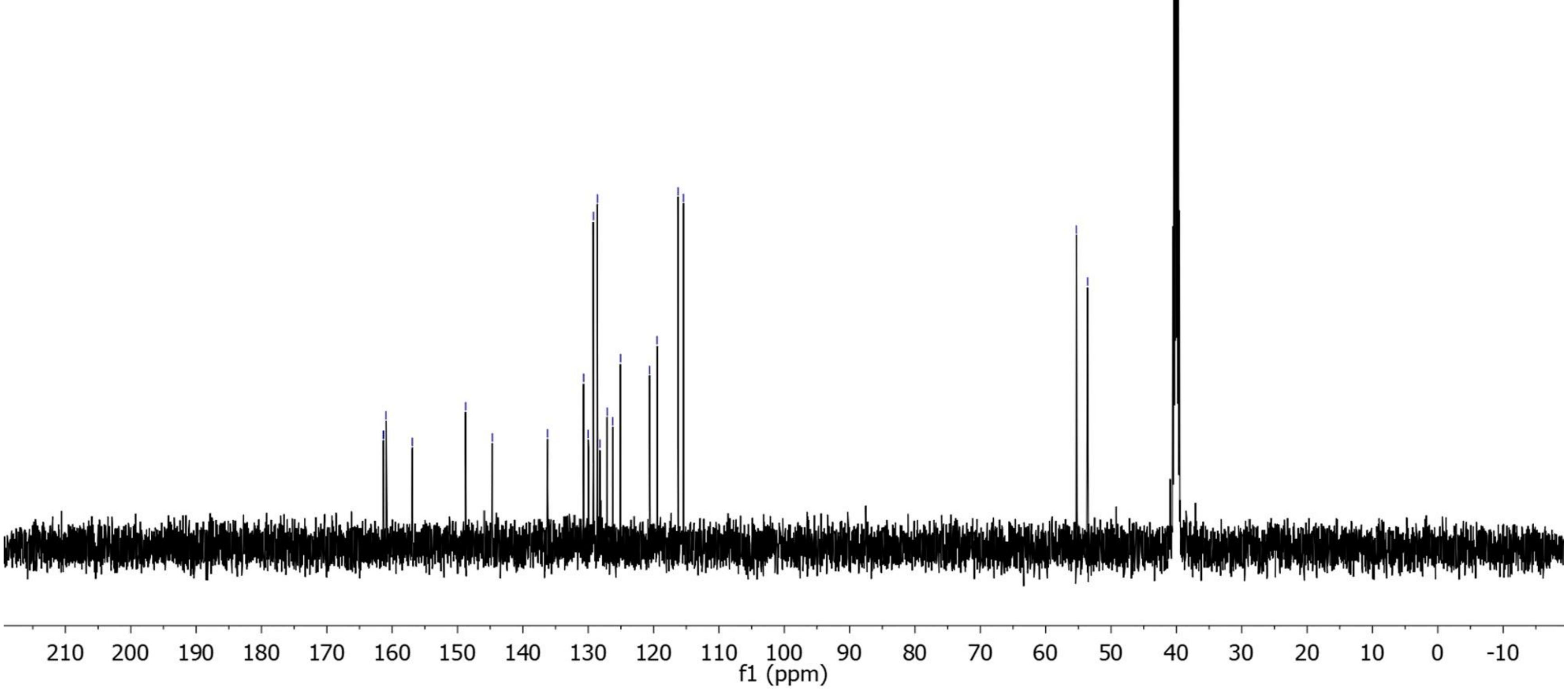

^{1}H NMR spectrum of 2-(4-methoxy-phenyl)-4-thiophen-2-yl-benzo[4,5]imidazo[1,2-a]pyrimidin-3-ylamine (**3r**)

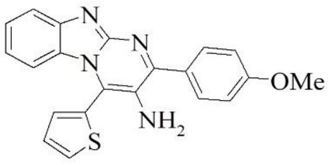

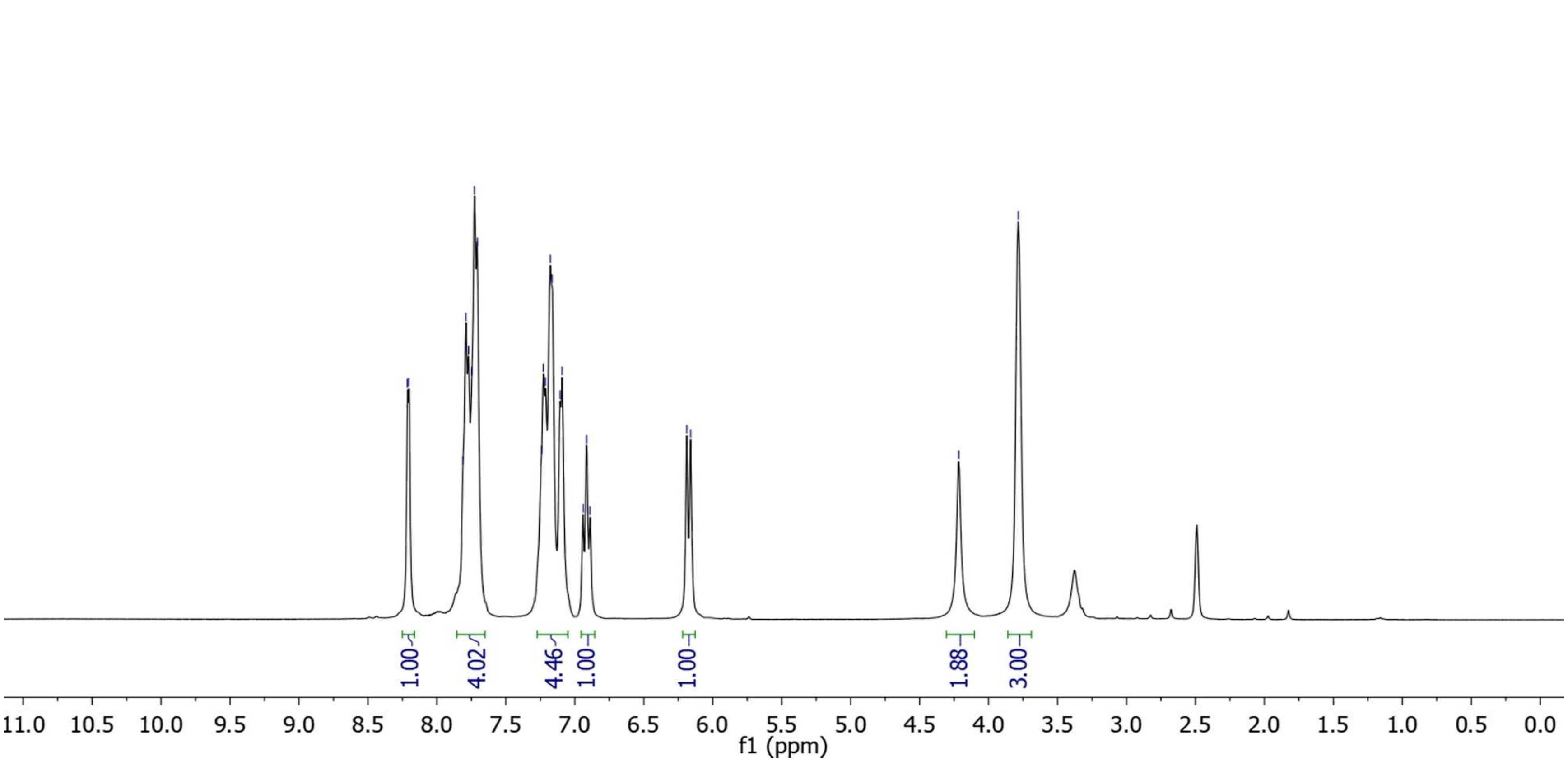

^{13}C NMR spectrum of 2-(4-methoxy-phenyl)-4-thiophen-2-yl-benzo[4,5]imidazo[1,2-a]pyrimidin-3-ylamine (**3r**)

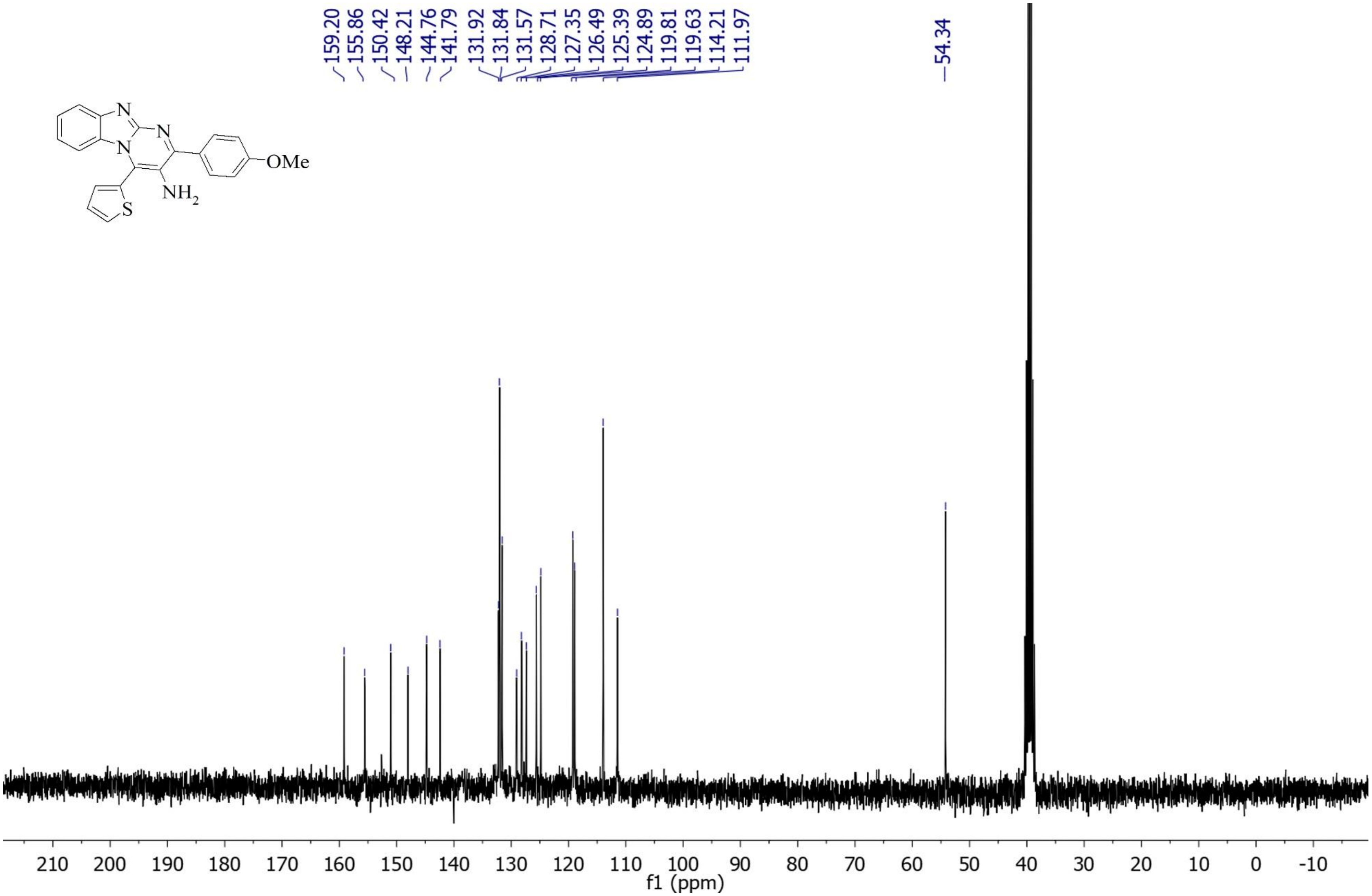

^{1}H NMR spectrum of 4-phenyl-2-thiophen-2-yl-benzo[4,5]imidazo[1,2-a]pyrimidin-3-ylamine (**3s**)

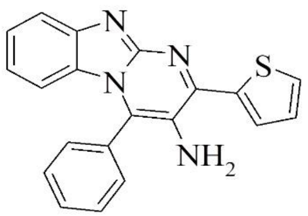

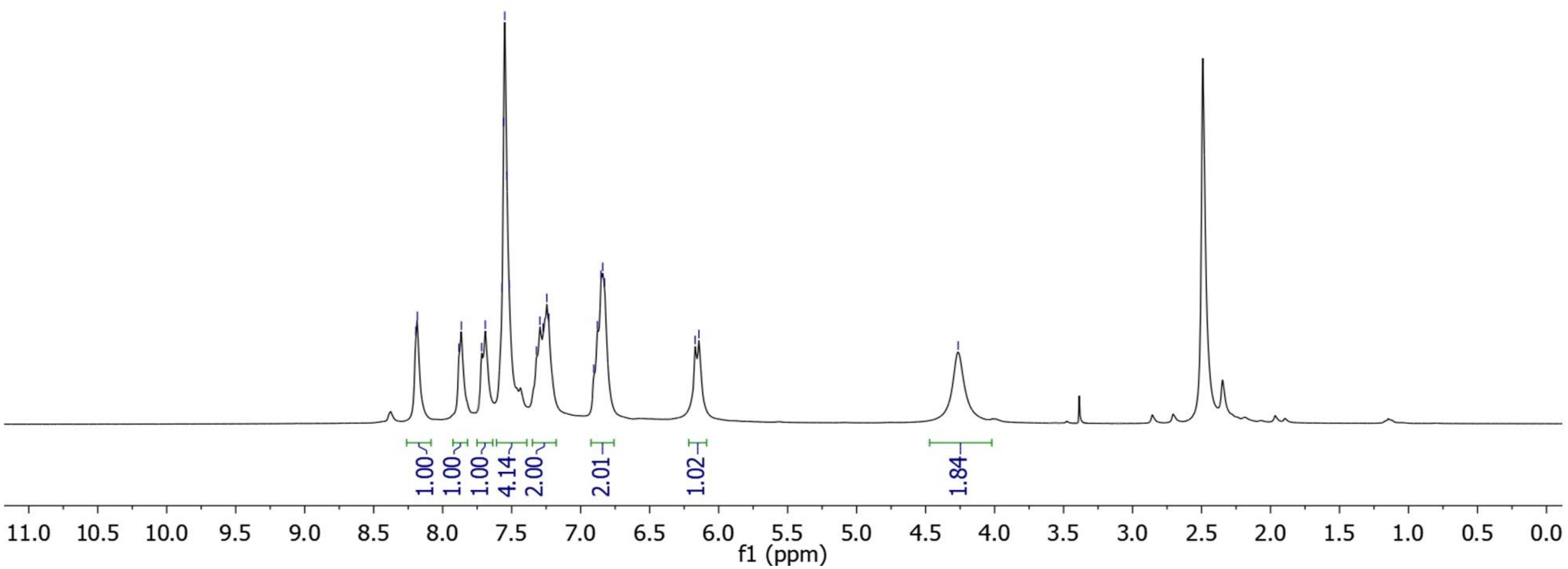

^{13}C NMR spectrum of spectrum of 4-phenyl-2-thiophen-2-yl-benzo[4,5]imidazo[1,2-a]pyrimidin-3-ylamine (**3s**)

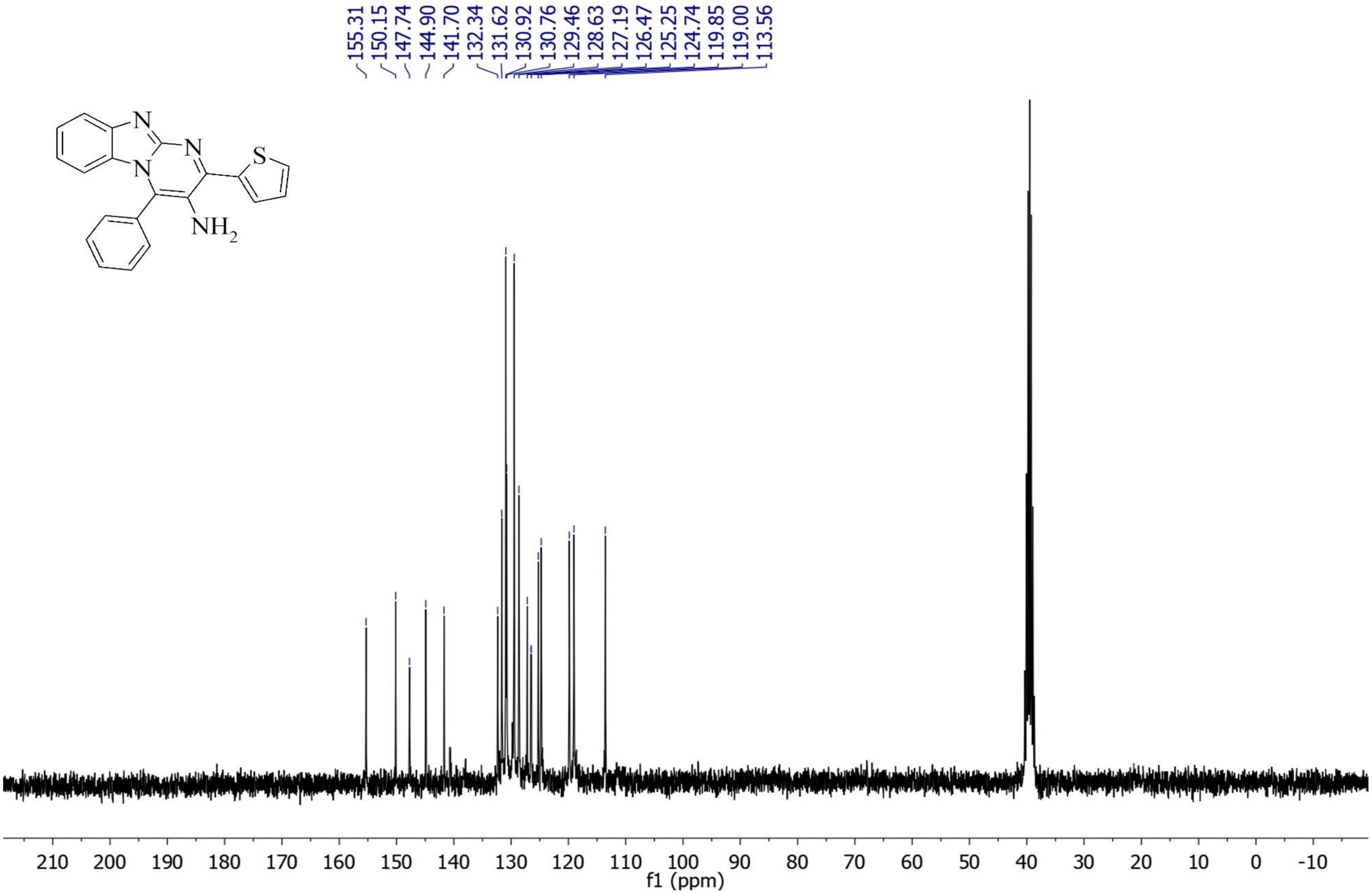

^{1}H NMR spectrum of 2-thiophen-2-yl-4-p-tolyl-benzo[4,5]imidazo[1,2-a]pyrimidin-3-ylamine (**3t**)

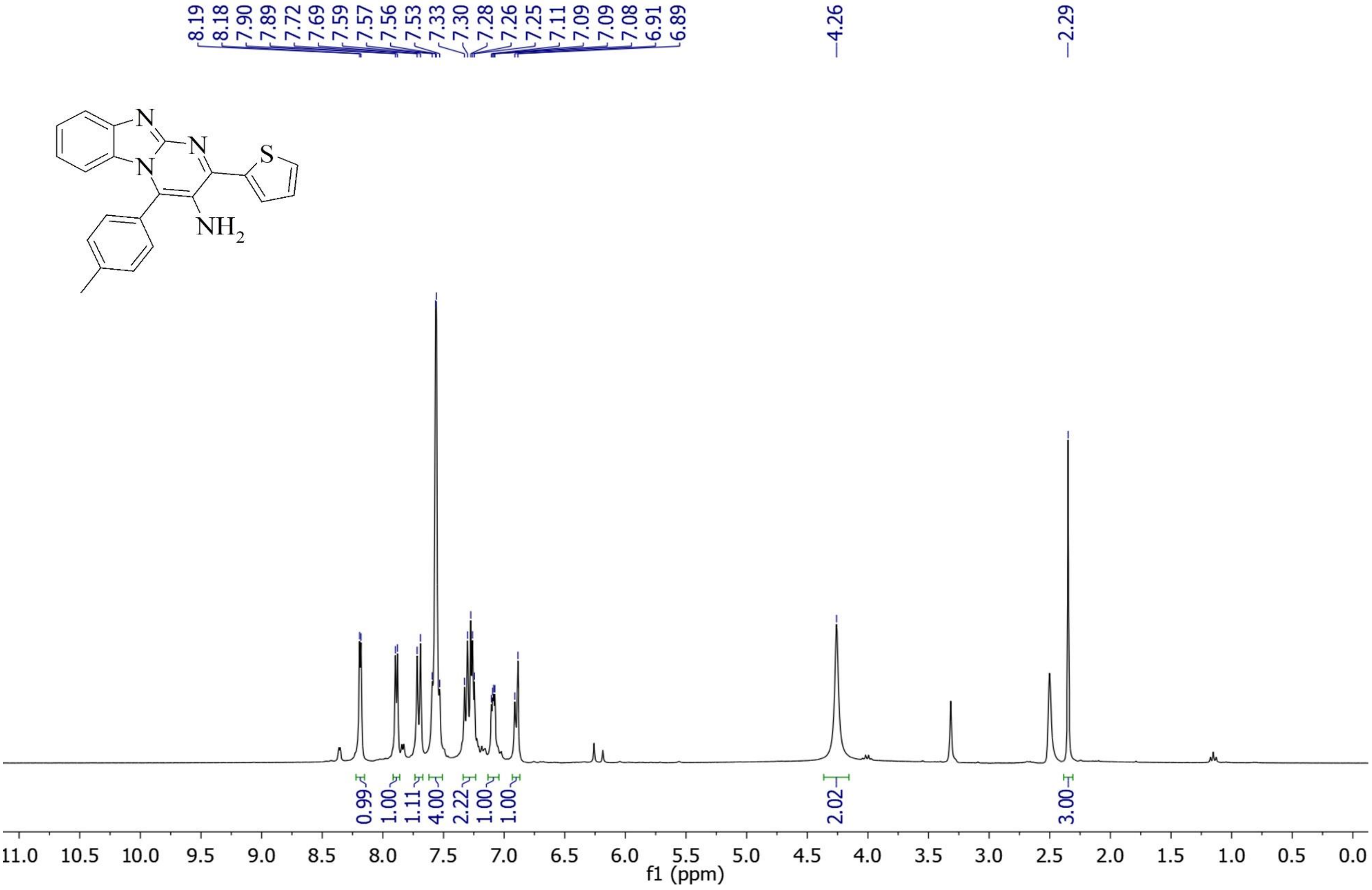

^{13}C NMR spectrum of 2-thiophen-2-yl-4-p-tolyl-benzo[4,5]imidazo[1,2-a]pyrimidin-3-ylamine (**3t**)

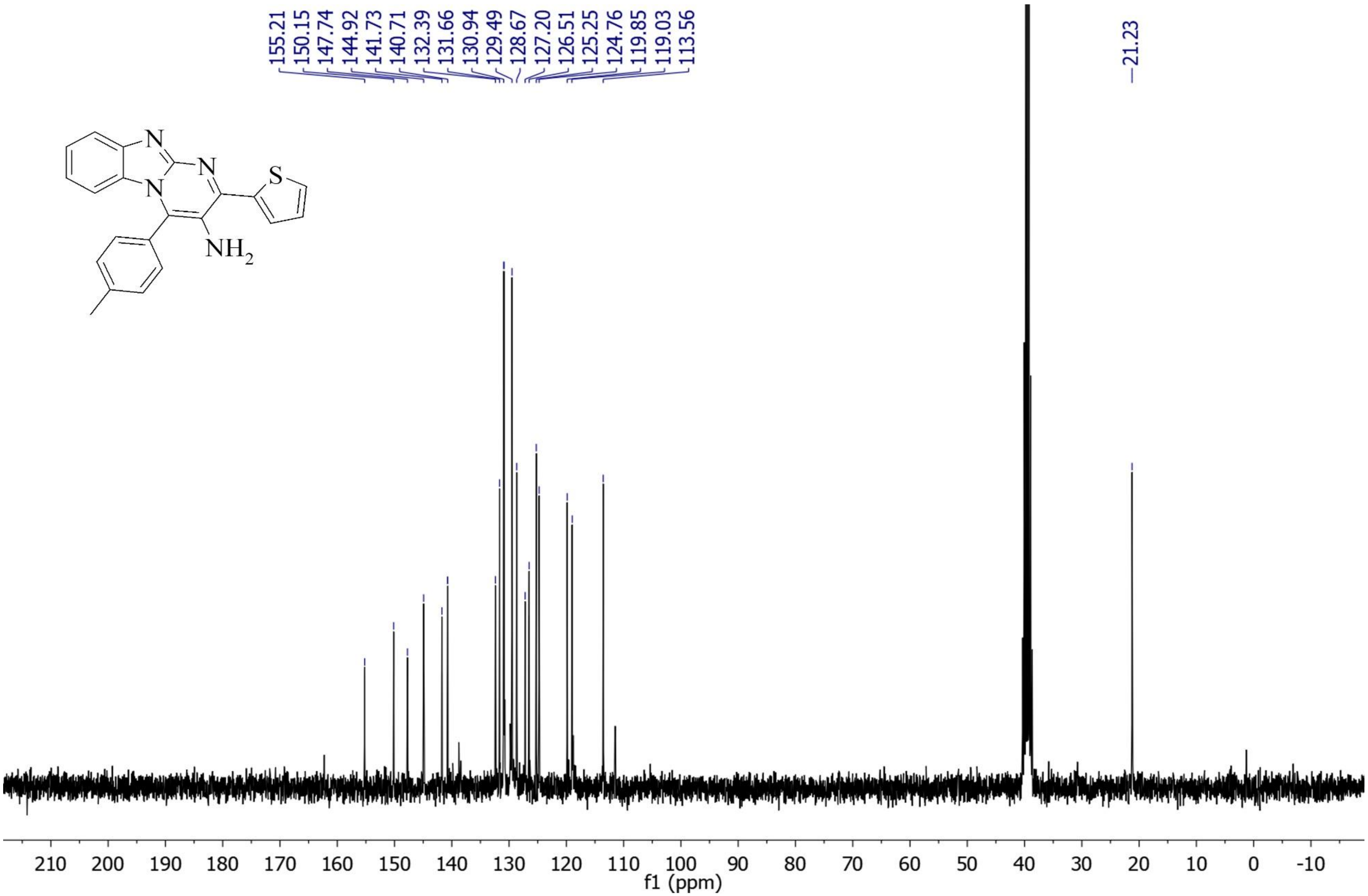

^{1}H NMR spectrum of 4-(4-methoxy-phenyl)-2-thiophen-2-yl-benzo[4,5]imidazo[1,2-a]pyrimidin-3-ylamine (**3u**)

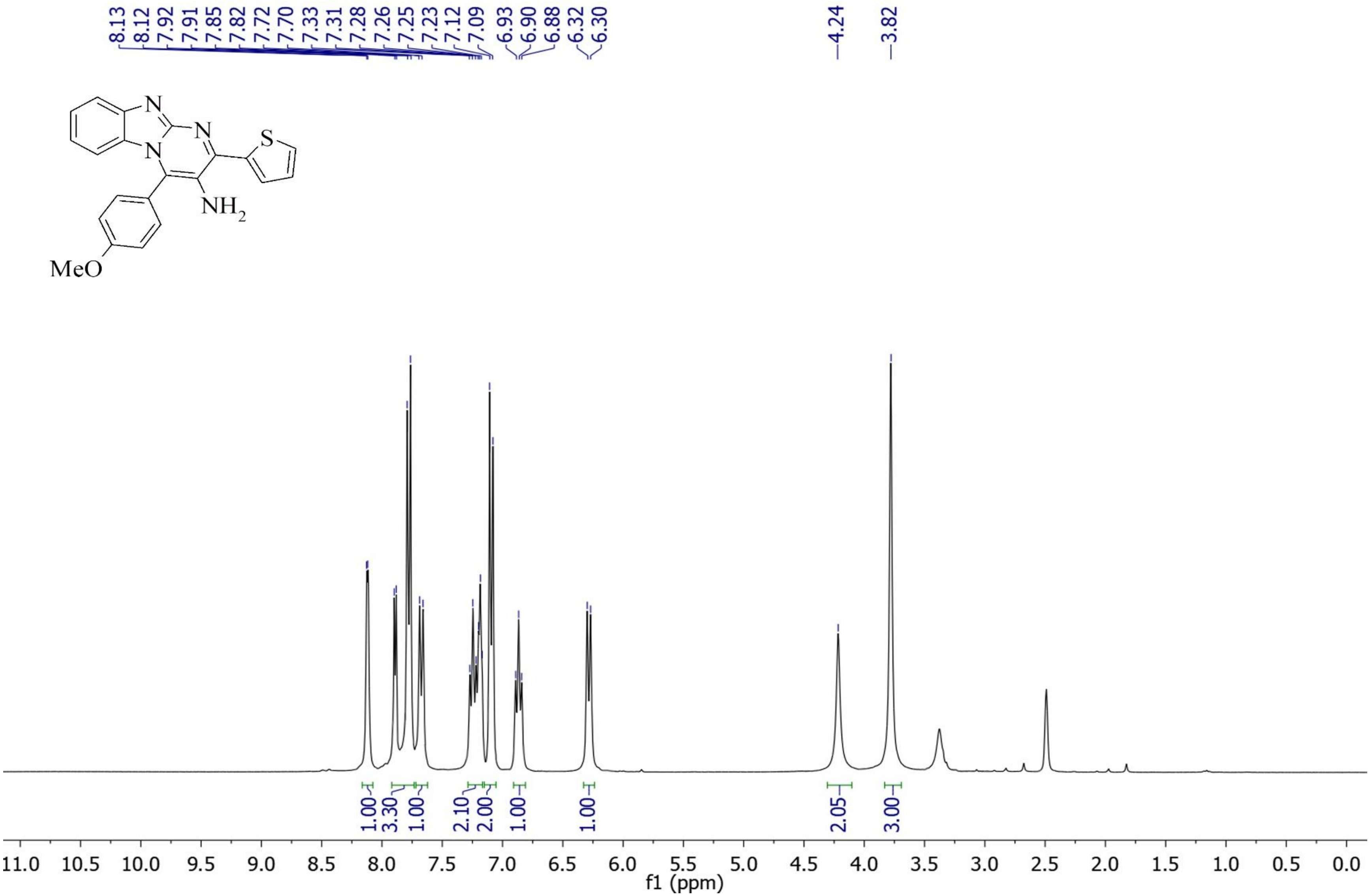

^{13}C NMR spectrum of spectrum of 4-(4-methoxy-phenyl)-2-thiophen-2-yl-benzo[4,5]imidazo[1,2-a]pyrimidin-3-ylamine (**3u**)

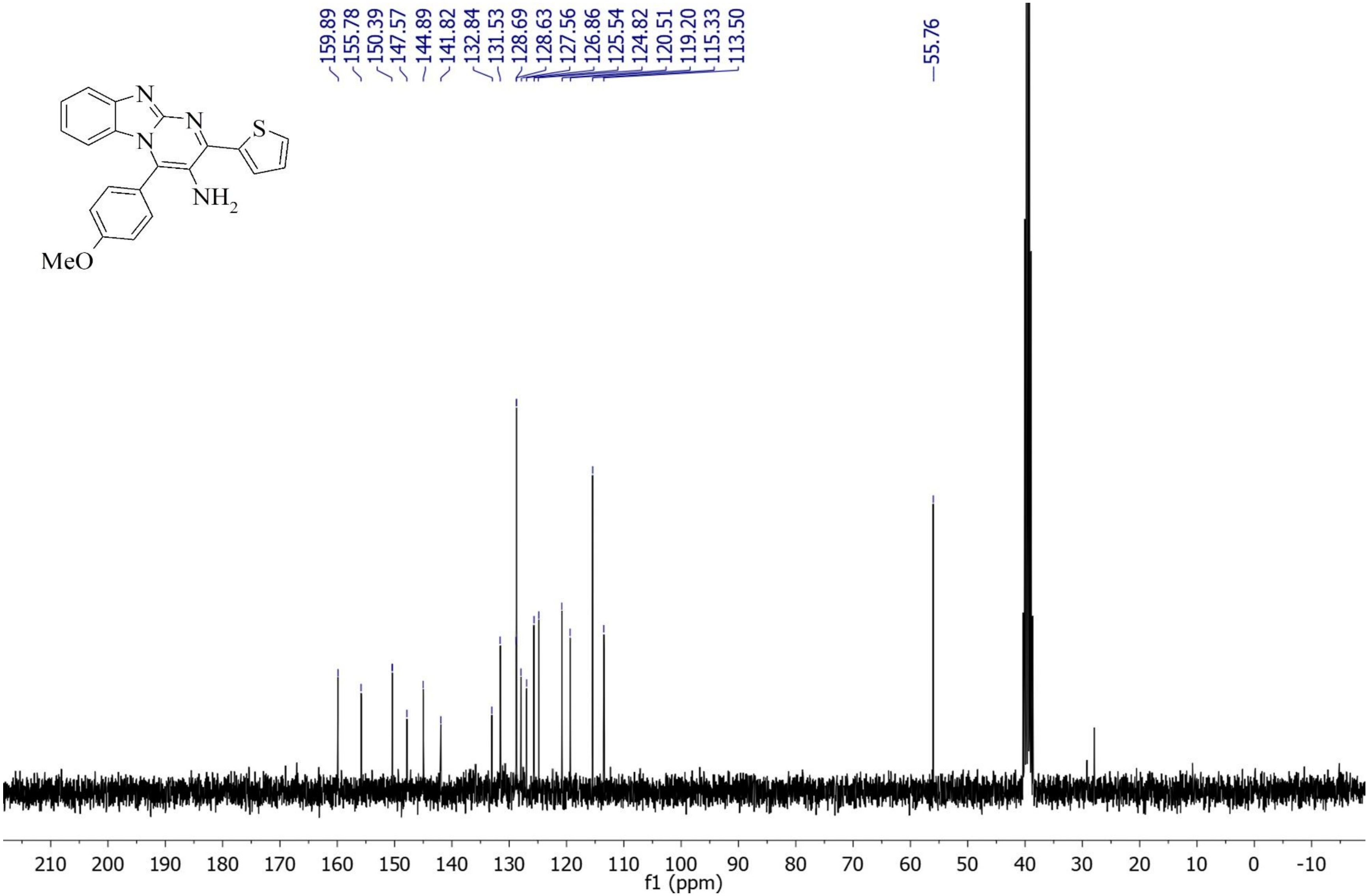

^{1}H NMR spectrum of 4-(4-chloro-phenyl)-2-thiophen-2-yl-benzo[4,5]imidazo[1,2-a]pyrimidin-3-ylamine (**3w**)

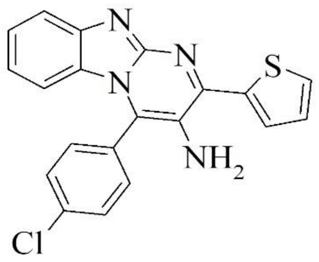

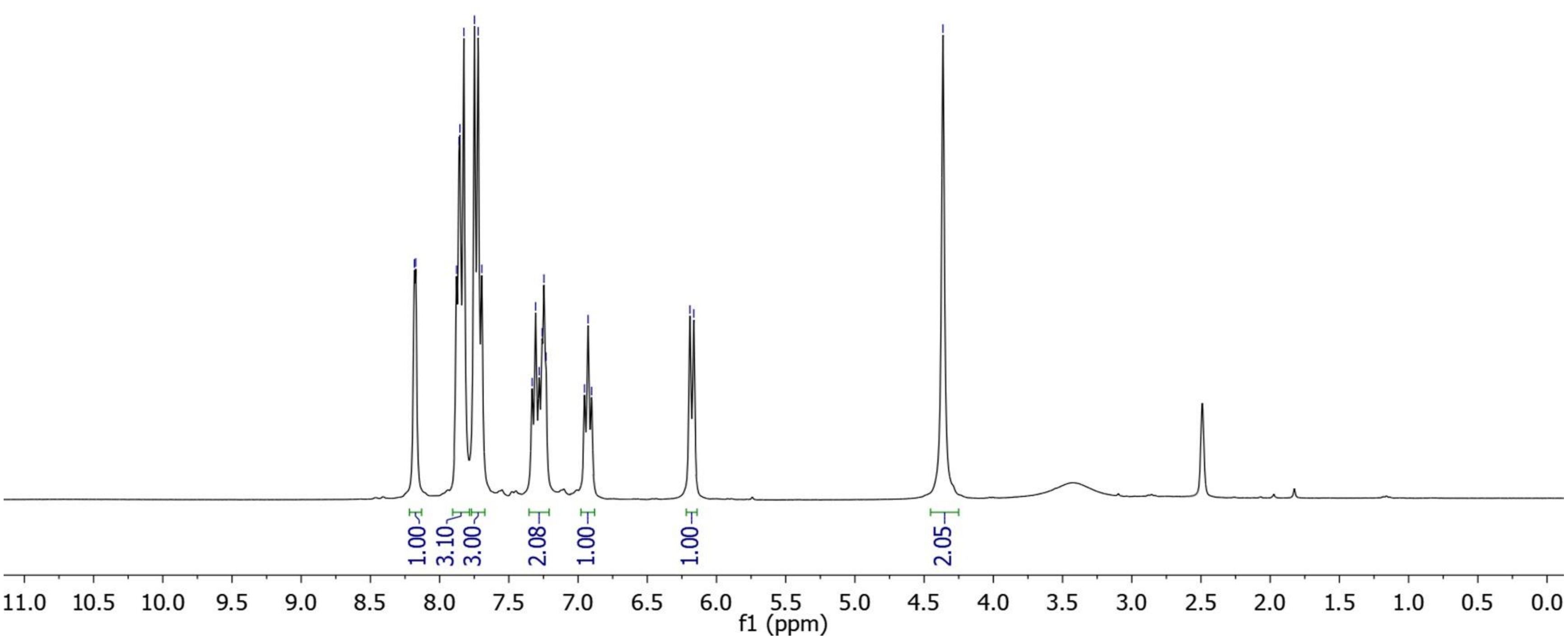

^{13}C NMR spectrum of 4-(4-chloro-phenyl)-2-thiophen-2-yl-benzo[4,5]imidazo[1,2-a]pyrimidin-3-ylamine (**3w**)

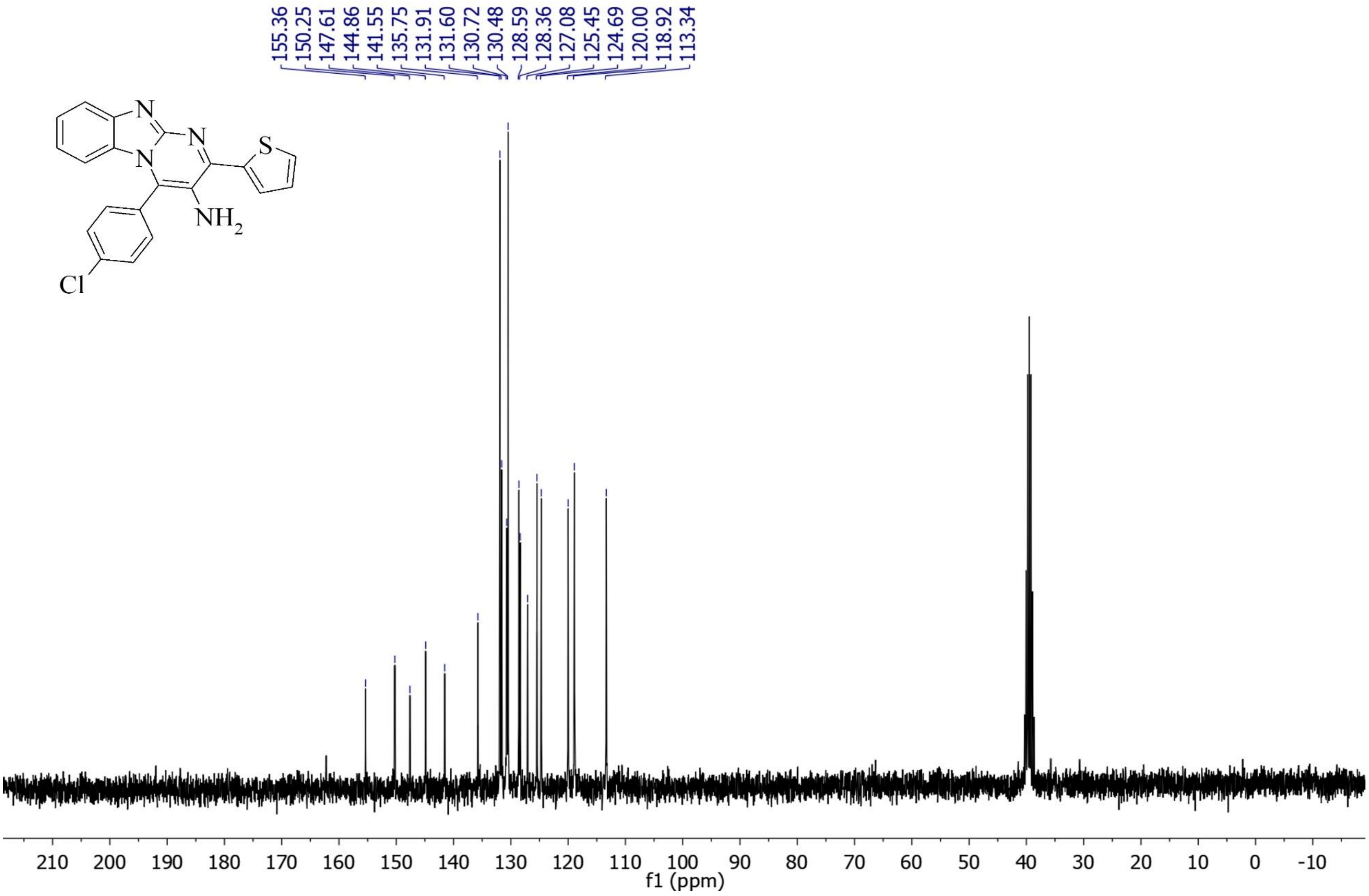

^{1}H NMR spectrum of 2,4-di-thiophen-2-yl-benzo[4,5]imidazo[1,2-a]pyrimidin-3-ylamine (**3x**)

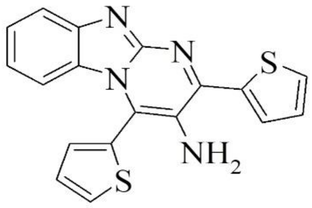

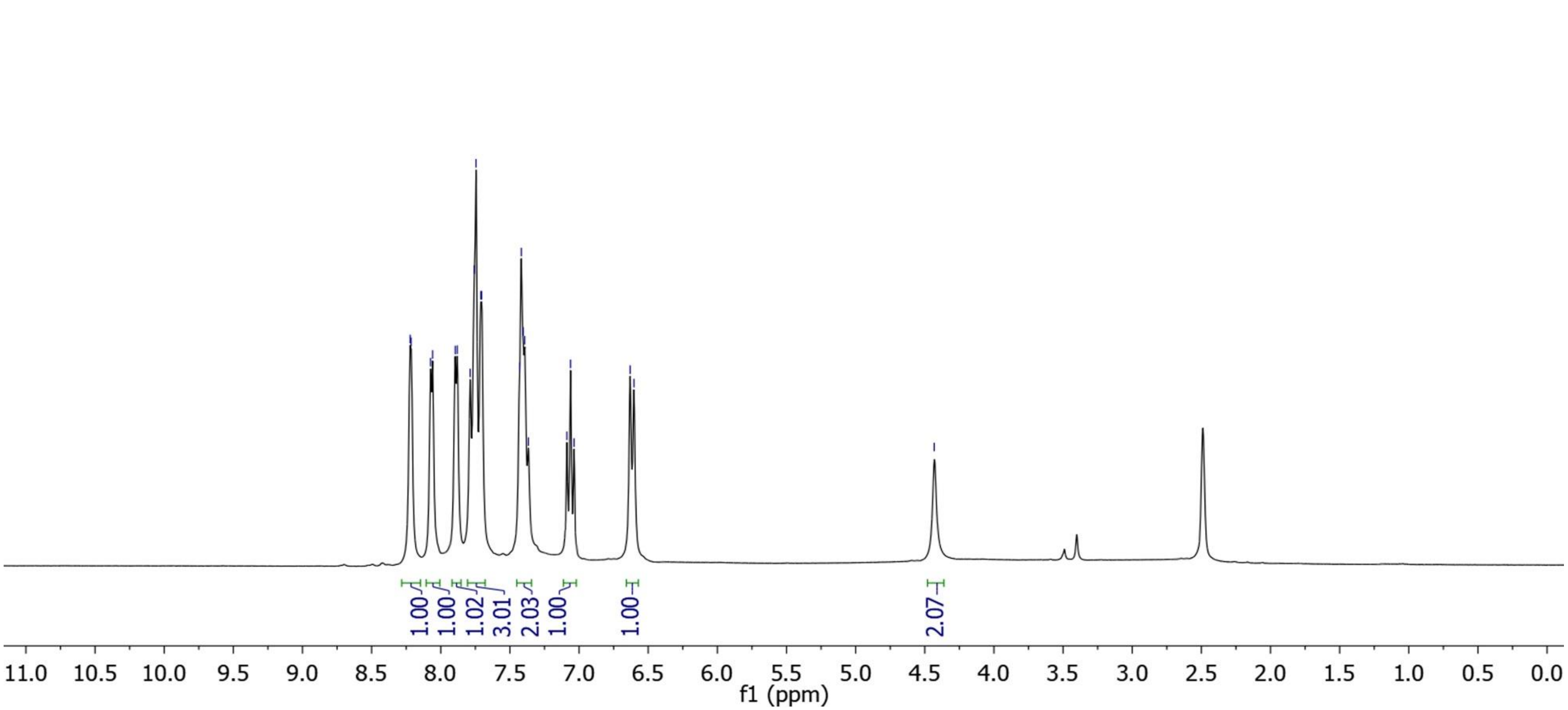

^{13}C NMR spectrum of 2,4-di-thiophen-2-yl-benzo[4,5]imidazo[1,2-a]pyrimidin-3-ylamine (**3x**)

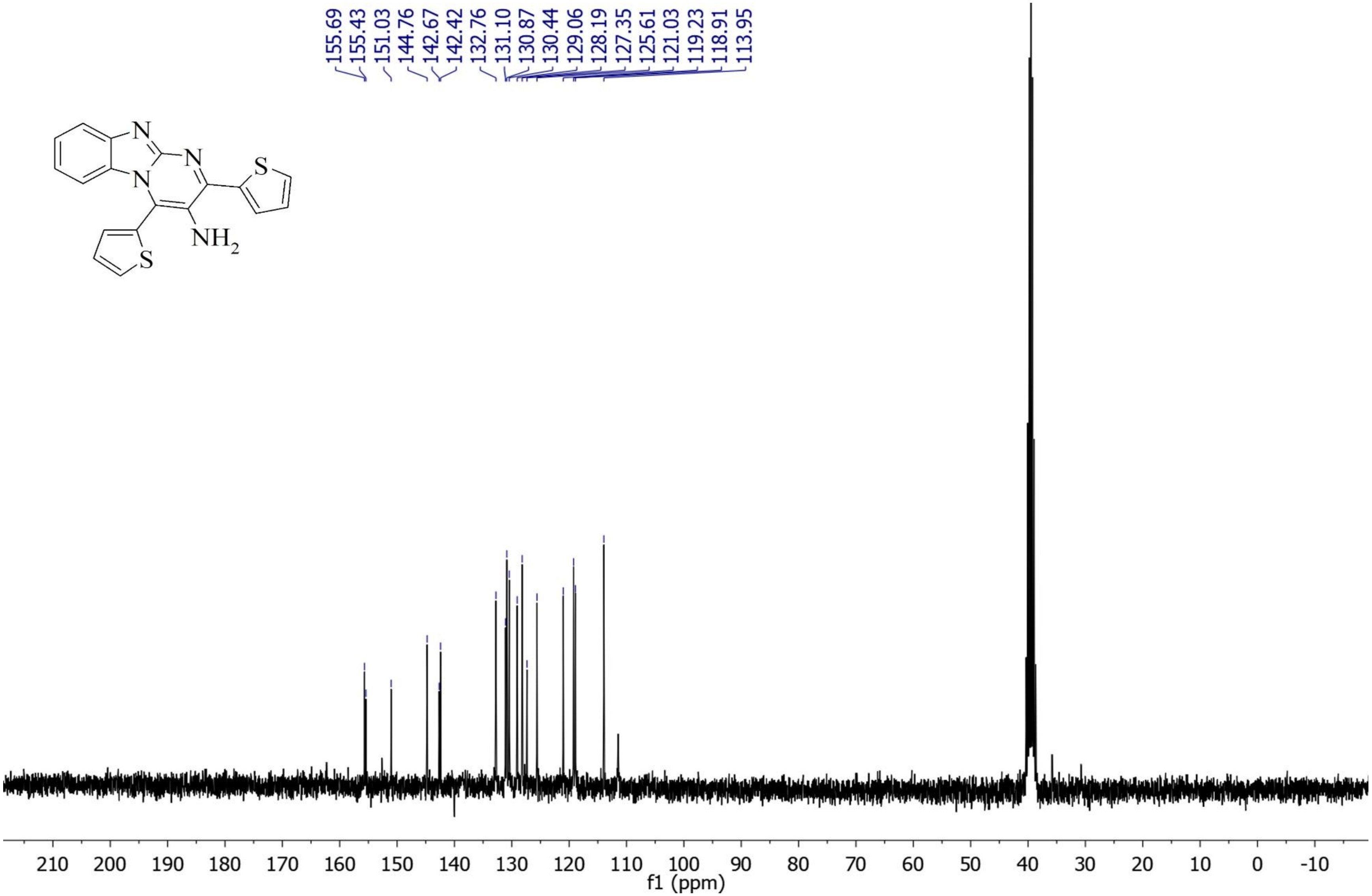

^{1}H NMR spectrum of 7,8-dichloro-2,4-diphenyl-benzo[4,5]imidazo[1,2-a]pyrimidin-3-ylamine (**3y**)

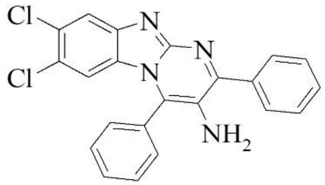

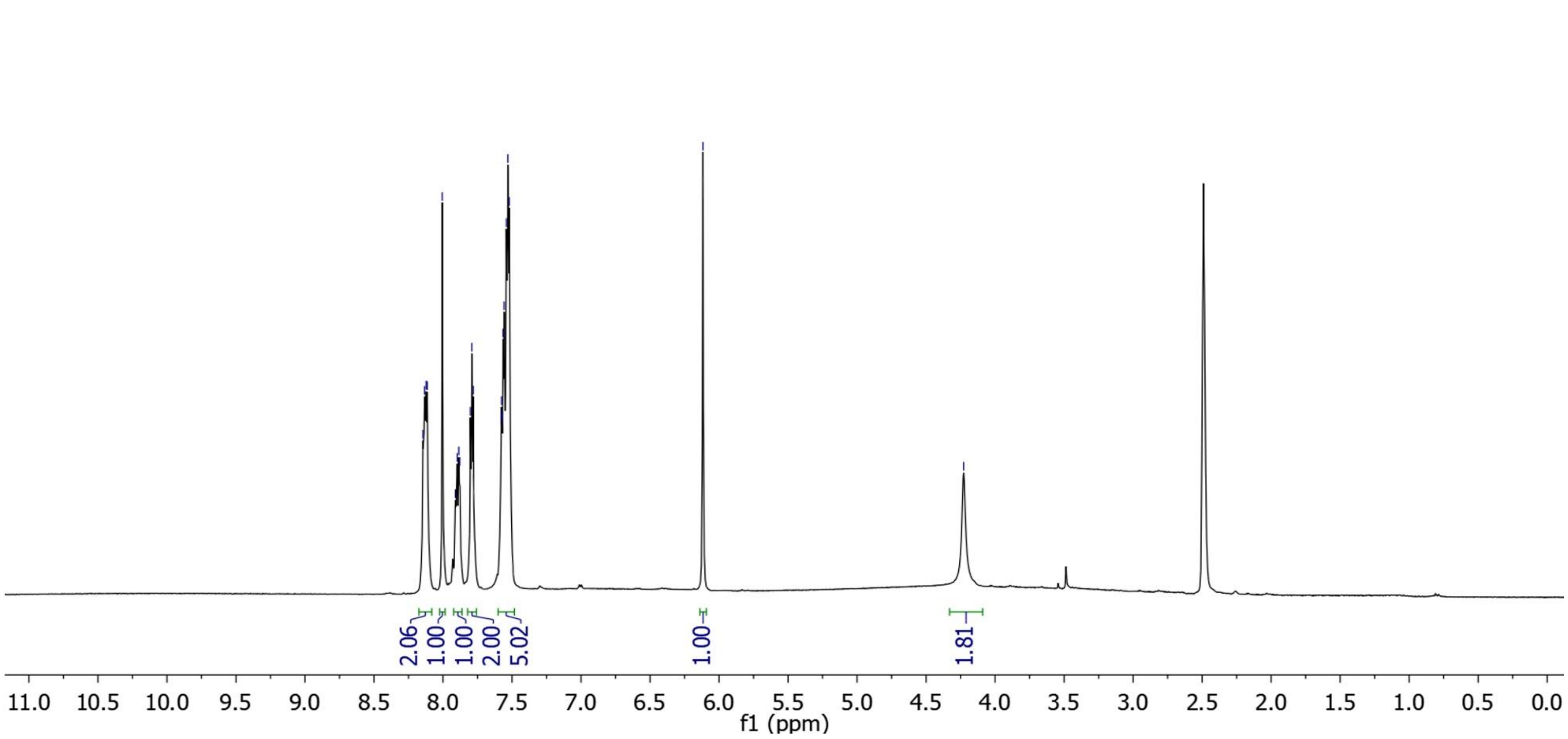

^{13}C NMR spectrum of 7,8-dichloro-2,4-diphenyl-benzo[4,5]imidazo[1,2-a]pyrimidin-3-ylamine (**3y**)

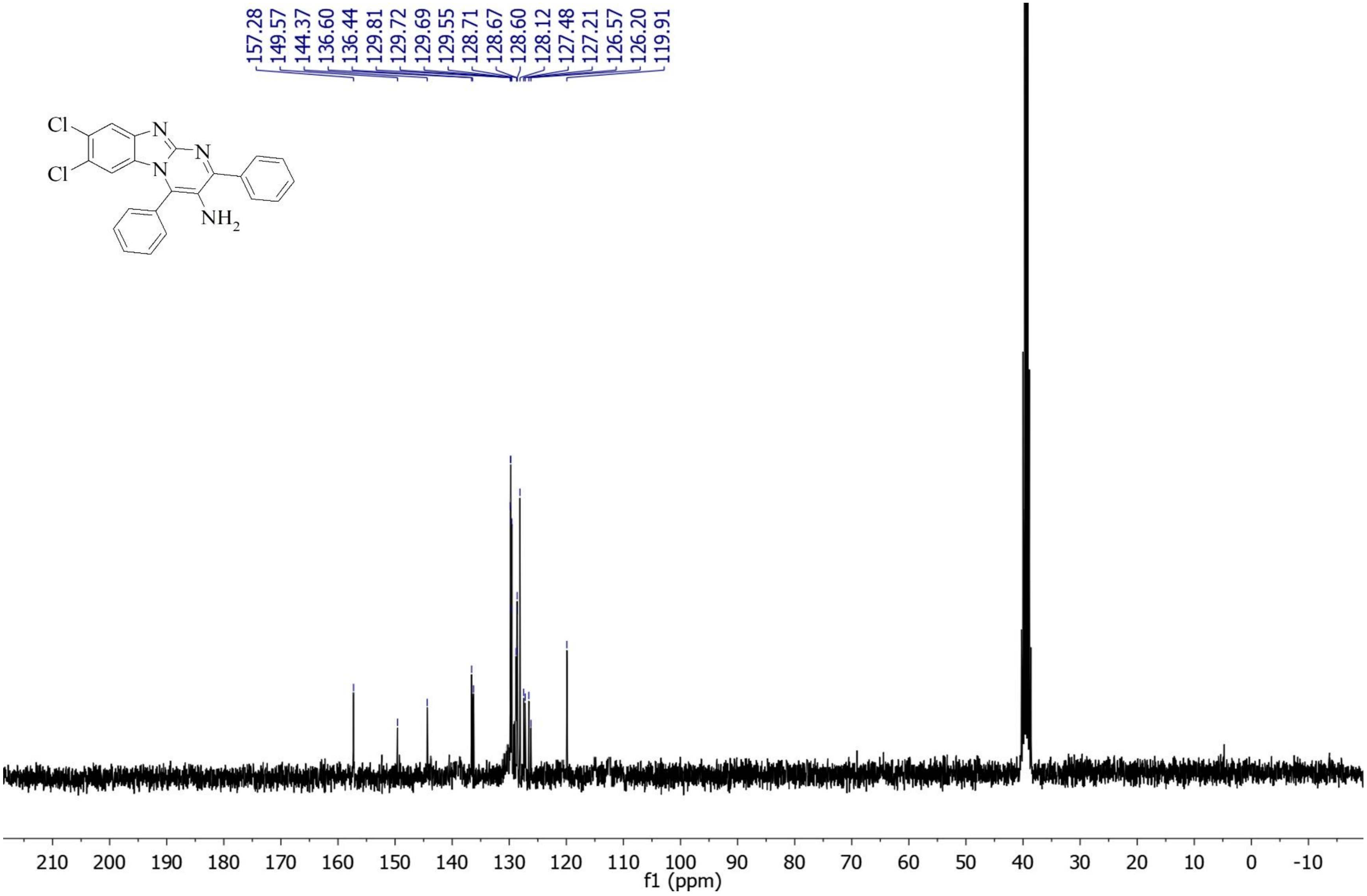

^{1}H NMR spectrum of 7,8-dichloro-4-(4-chloro-phenyl)-2-phenyl-benzo[4,5]imidazo[1,2-a]pyrimidin-3-ylamine (**3z**)

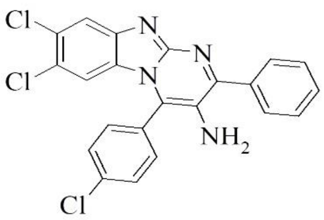

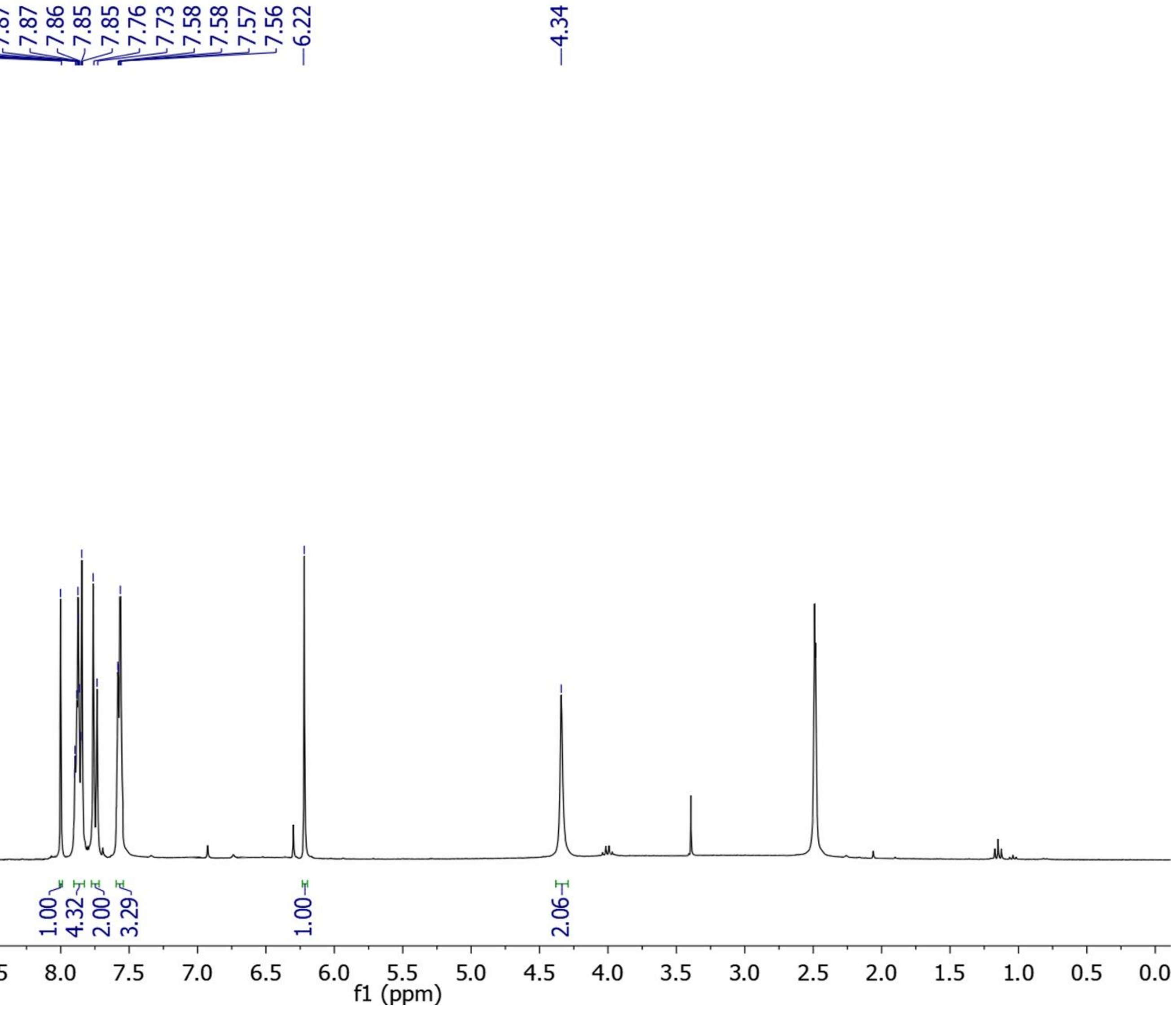

^{13}C NMR spectrum of 7,8-dichloro-4-(4-chloro-phenyl)-2-phenyl-benzo[4,5]imidazo[1,2-a]pyrimidin-3-ylamine (**3z**)

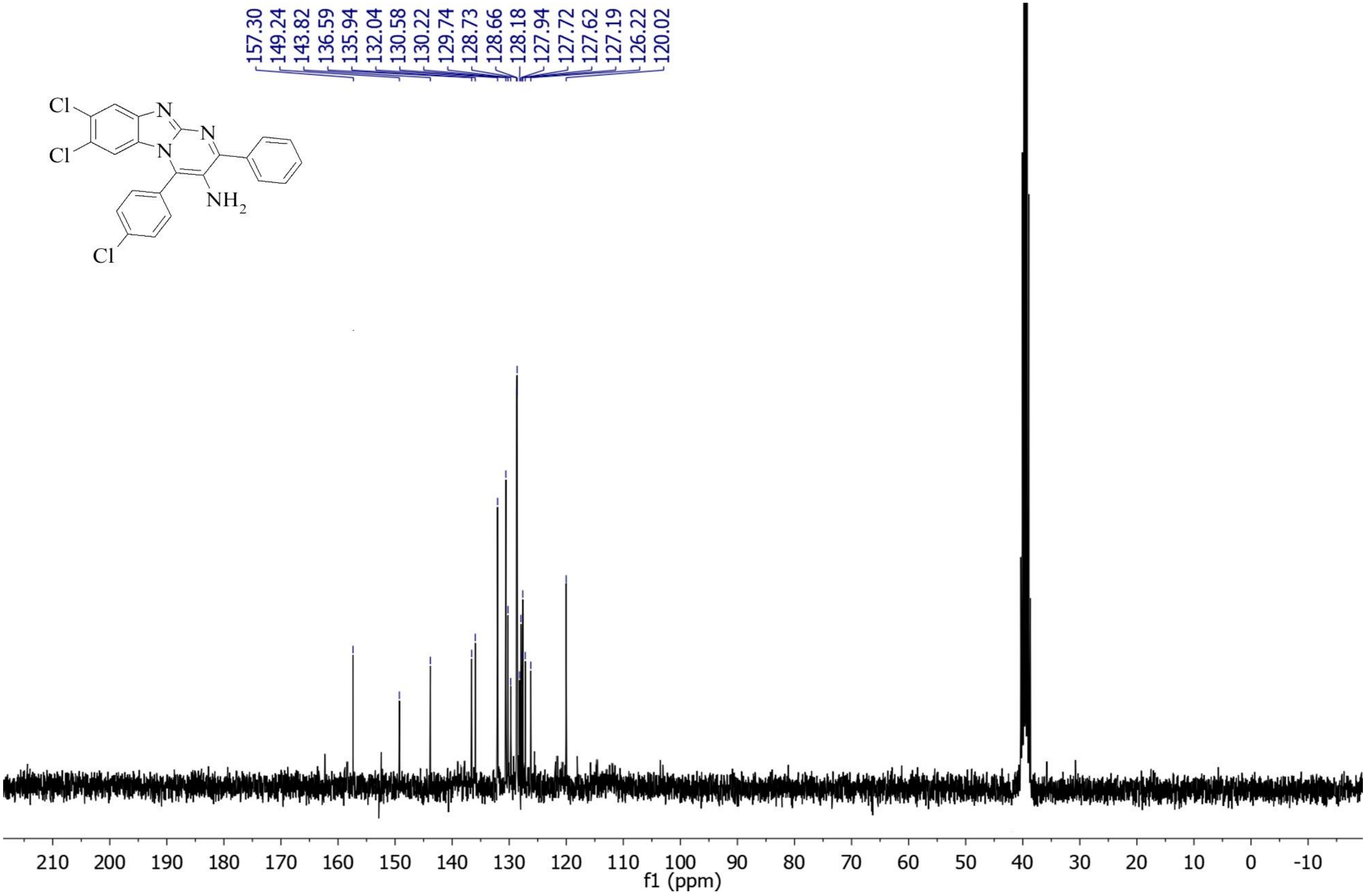

^1H NMR spectrum of 7,8-dichloro-2-(4-chloro-phenyl)-4-phenyl-benzo[4,5]imidazo[1,2-a]pyrimidin-3-ylamine (**3aa**)

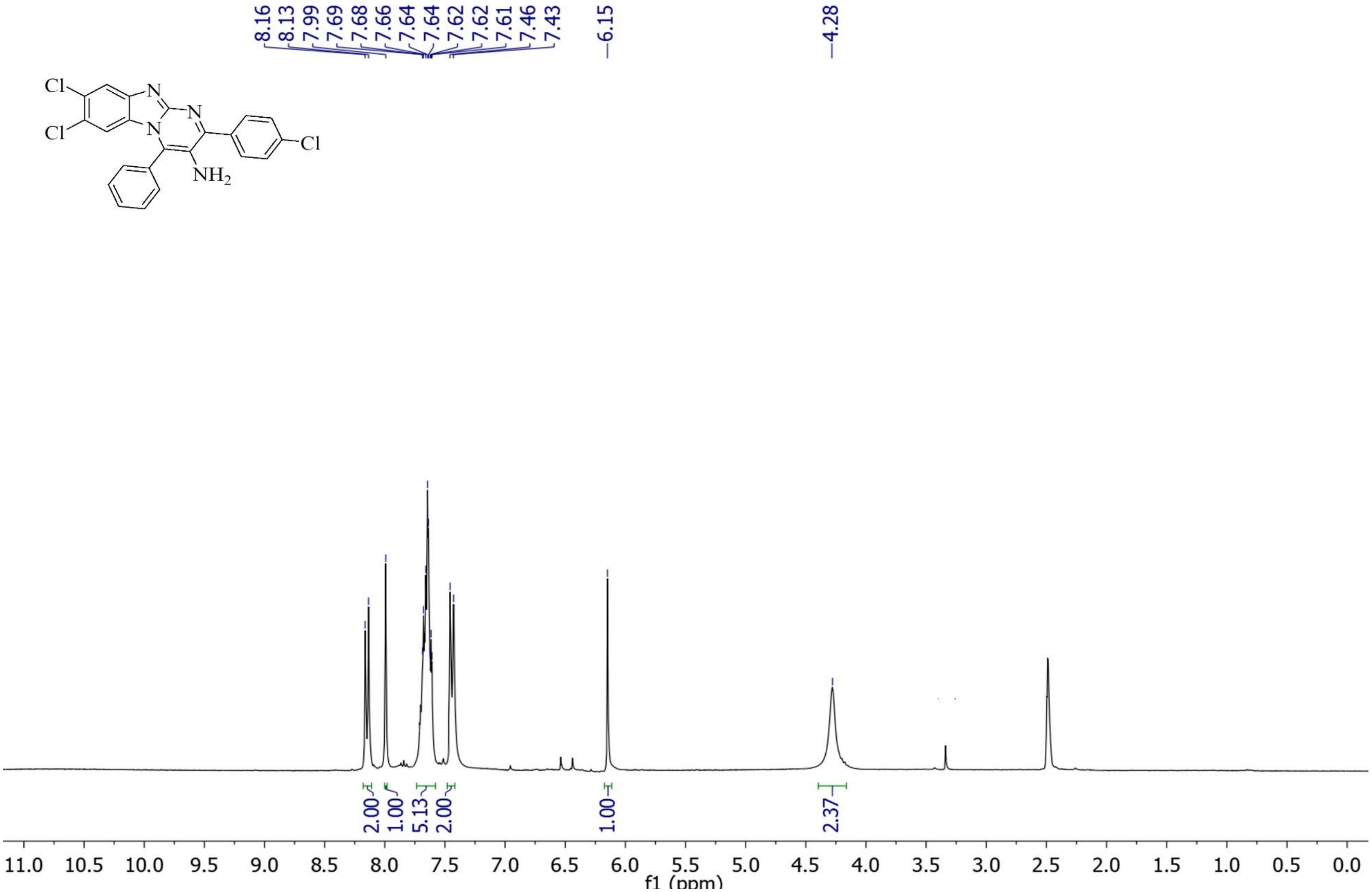

^{13}C NMR spectrum of 7,8-dichloro-2-(4-chloro-phenyl)-4-phenyl-benzo[4,5]imidazo[1,2-a]pyrimidin-3-ylamine (**3aa**)

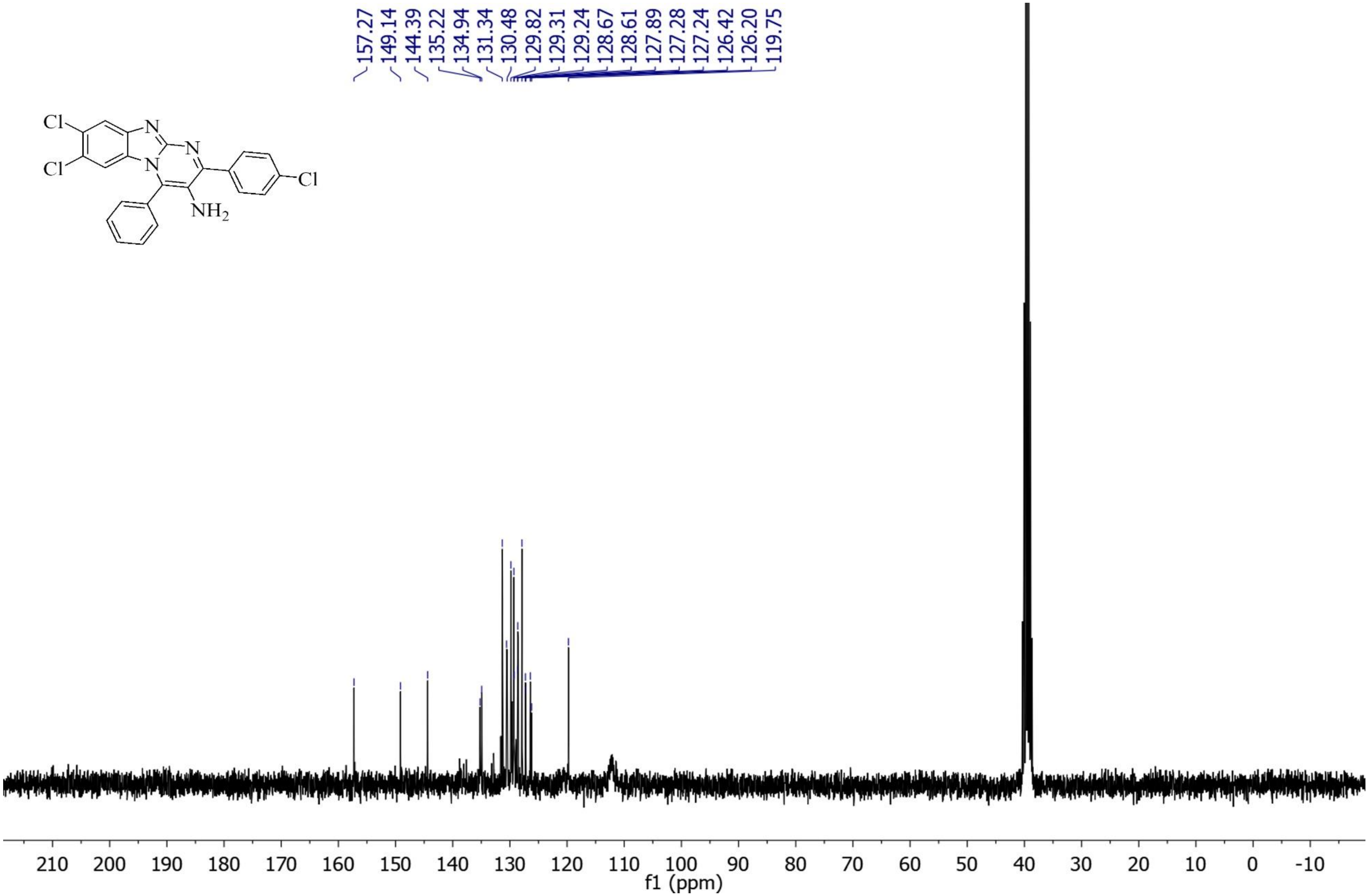

^{1}H NMR spectrum of 7,8-dichloro-2-(4-chloro-phenyl)-4-p-tolyl-benzo[4,5]imidazo[1,2-a]pyrimidin-3-ylamine (**3ab**)

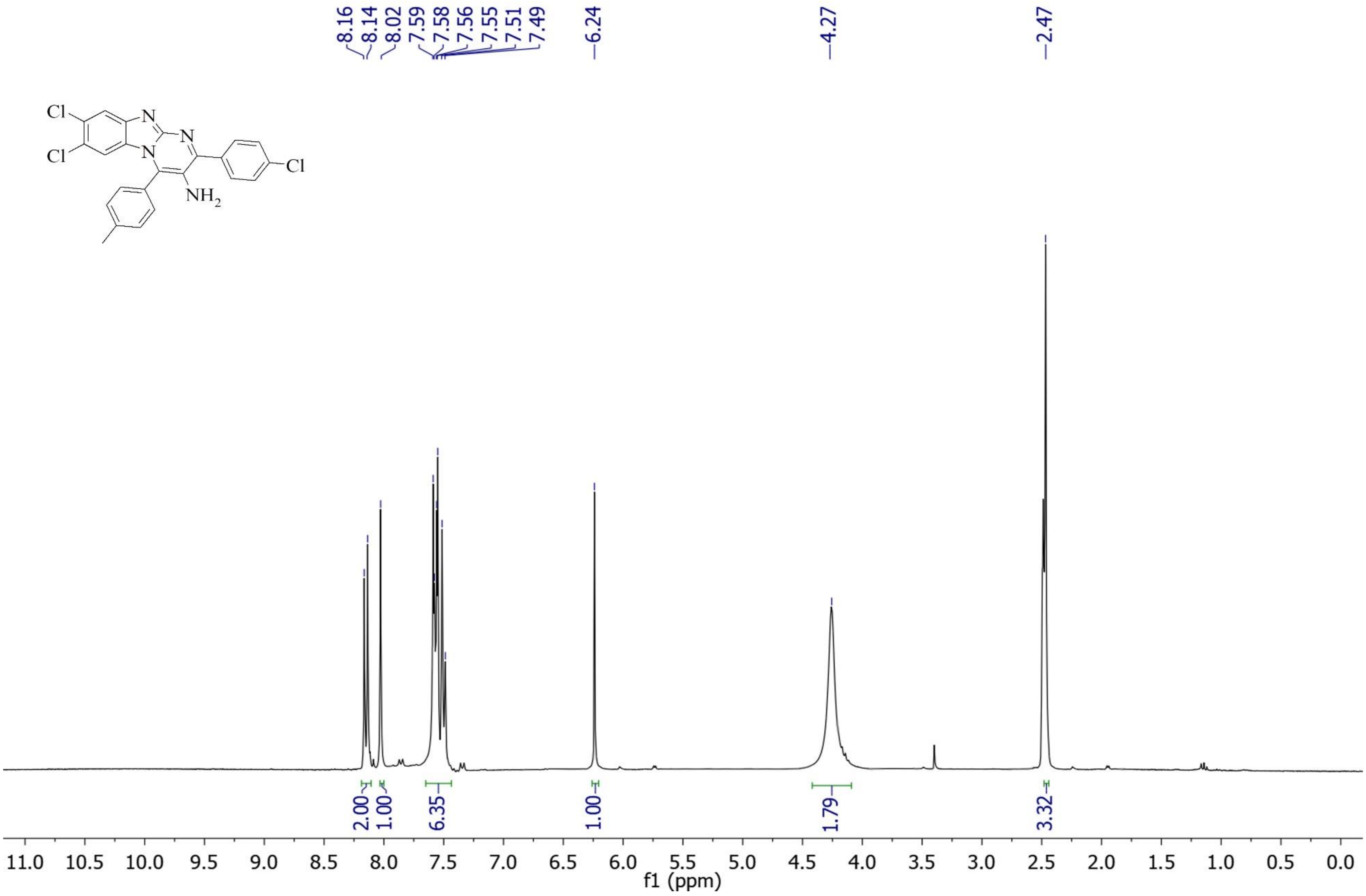

^{13}C NMR spectrum of 7,8-dichloro-2-(4-chloro-phenyl)-4-p-tolyl-benzo[4,5]imidazo[1,2-a]pyrimidin-3-ylamine (**3ab**)

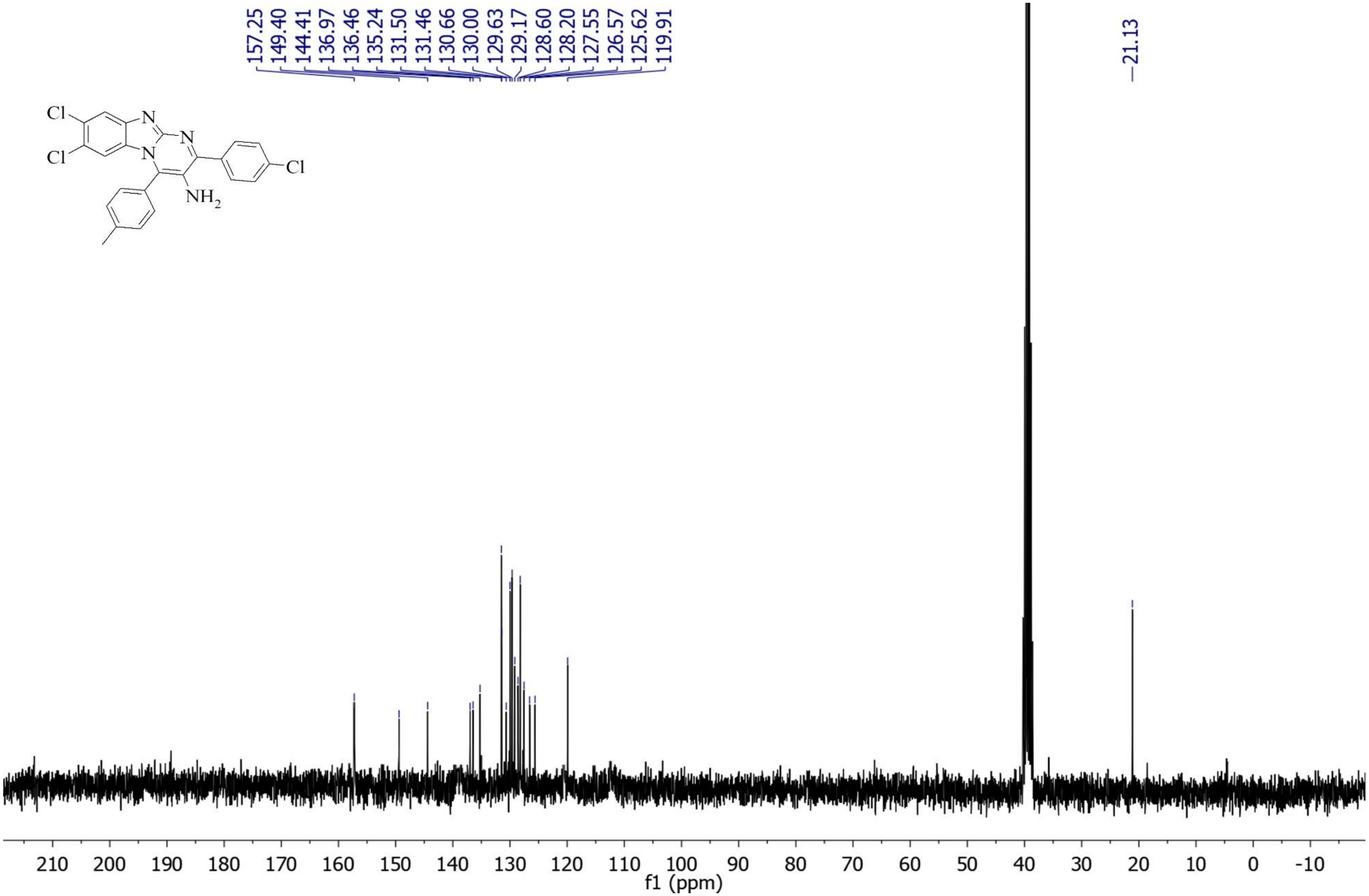

^{1}H NMR spectrum of 7,8-dichloro-2-(4-chloro-phenyl)-4-(4-methoxy-phenyl)-benzo[4,5]imidazo[1,2-a]pyrimidin-3-ylamine (**3ac**)

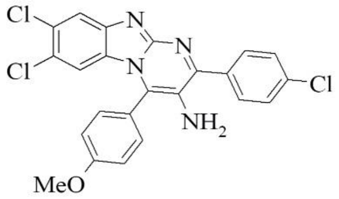

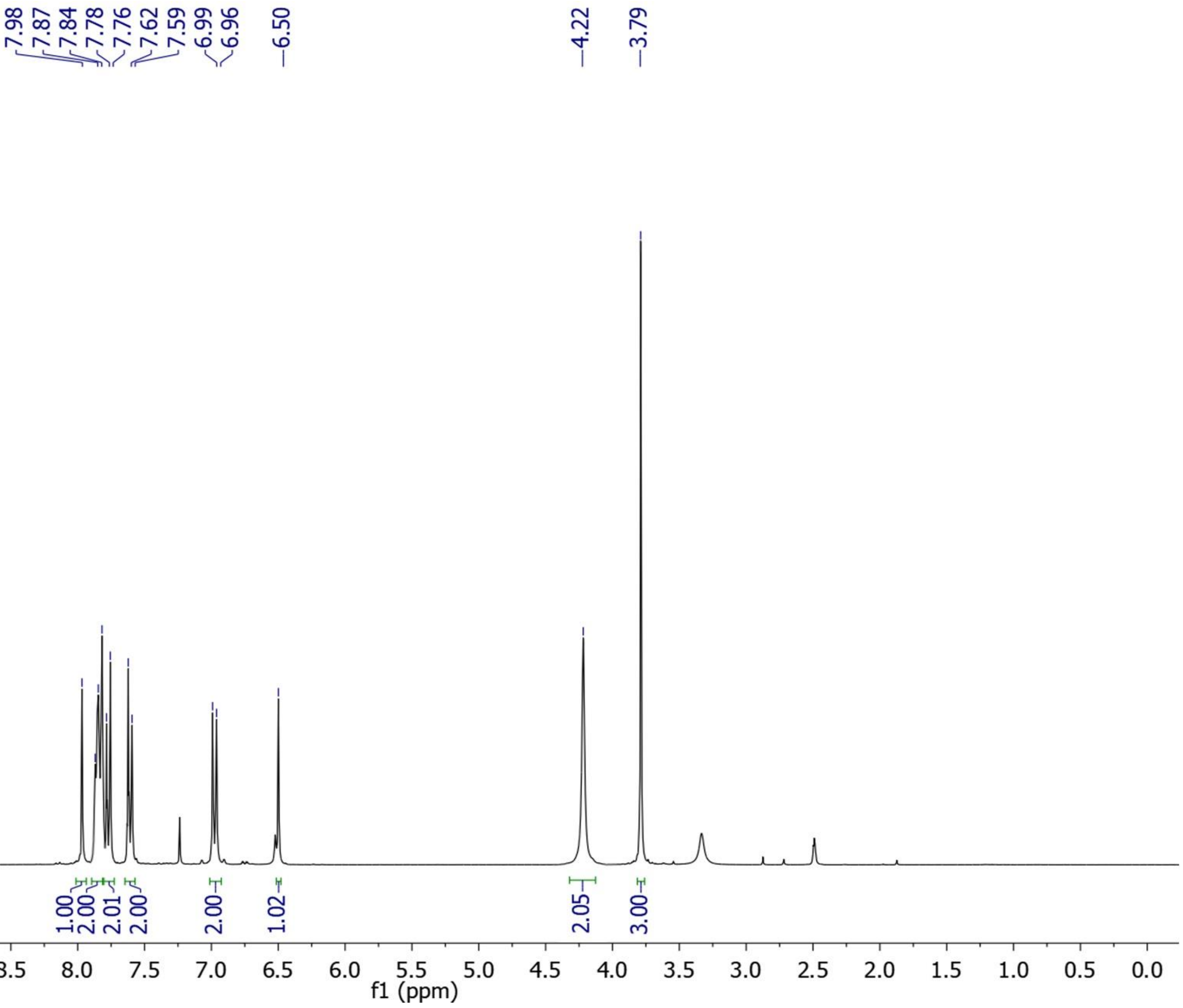

^{13}C NMR spectrum of 7,8-dichloro-2-(4-chloro-phenyl)-4-(4-methoxy-phenyl)-benzo[4,5]imidazo[1,2-a]pyrimidin-3-ylamine (**3ac**)

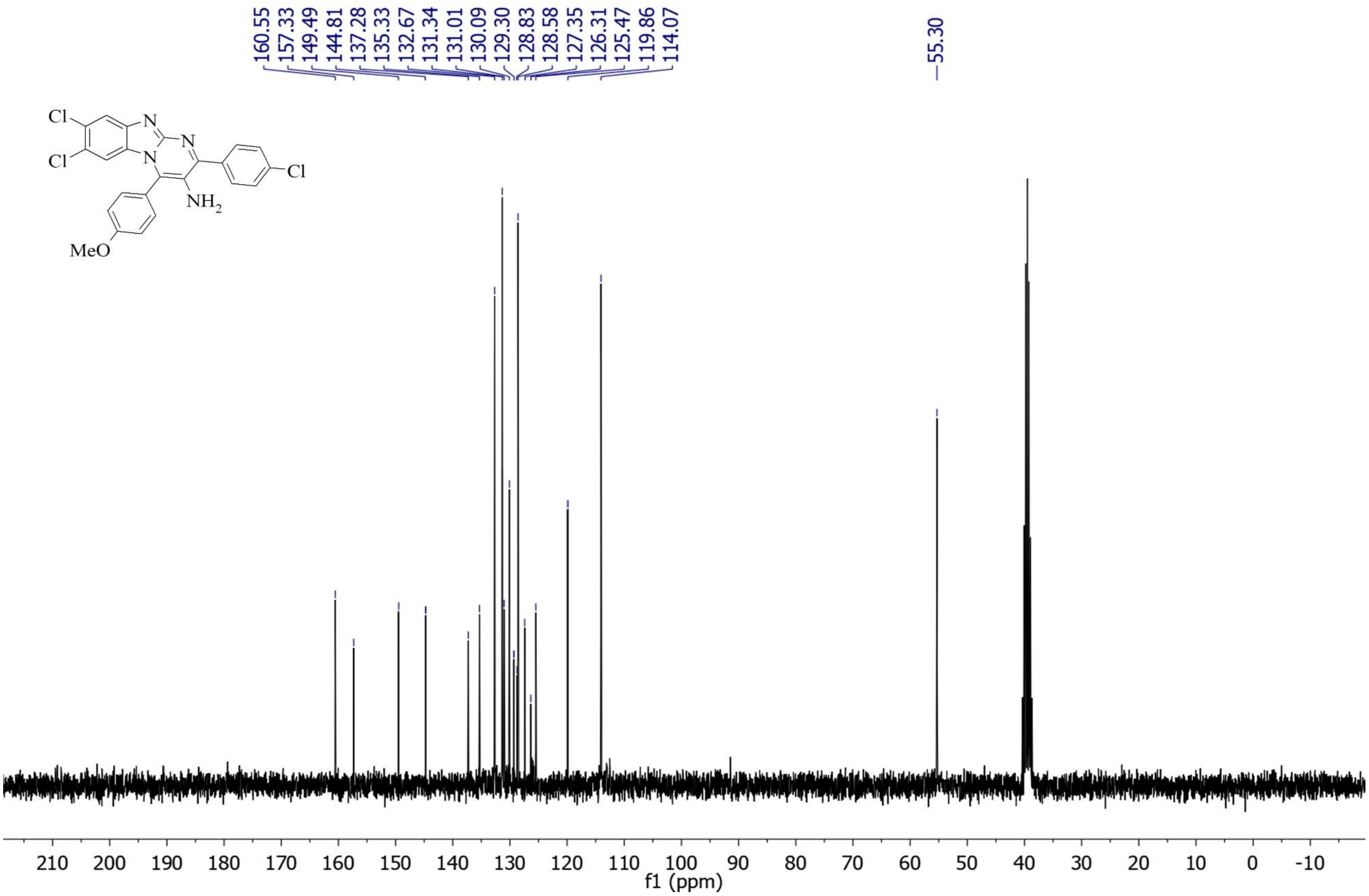

^{1}H NMR spectrum of 7,8-dichloro-2,4-bis-(4-chloro-phenyl)-benzo[4,5]imidazo[1,2-a]pyrimidin-3-ylamine (**3ad**)

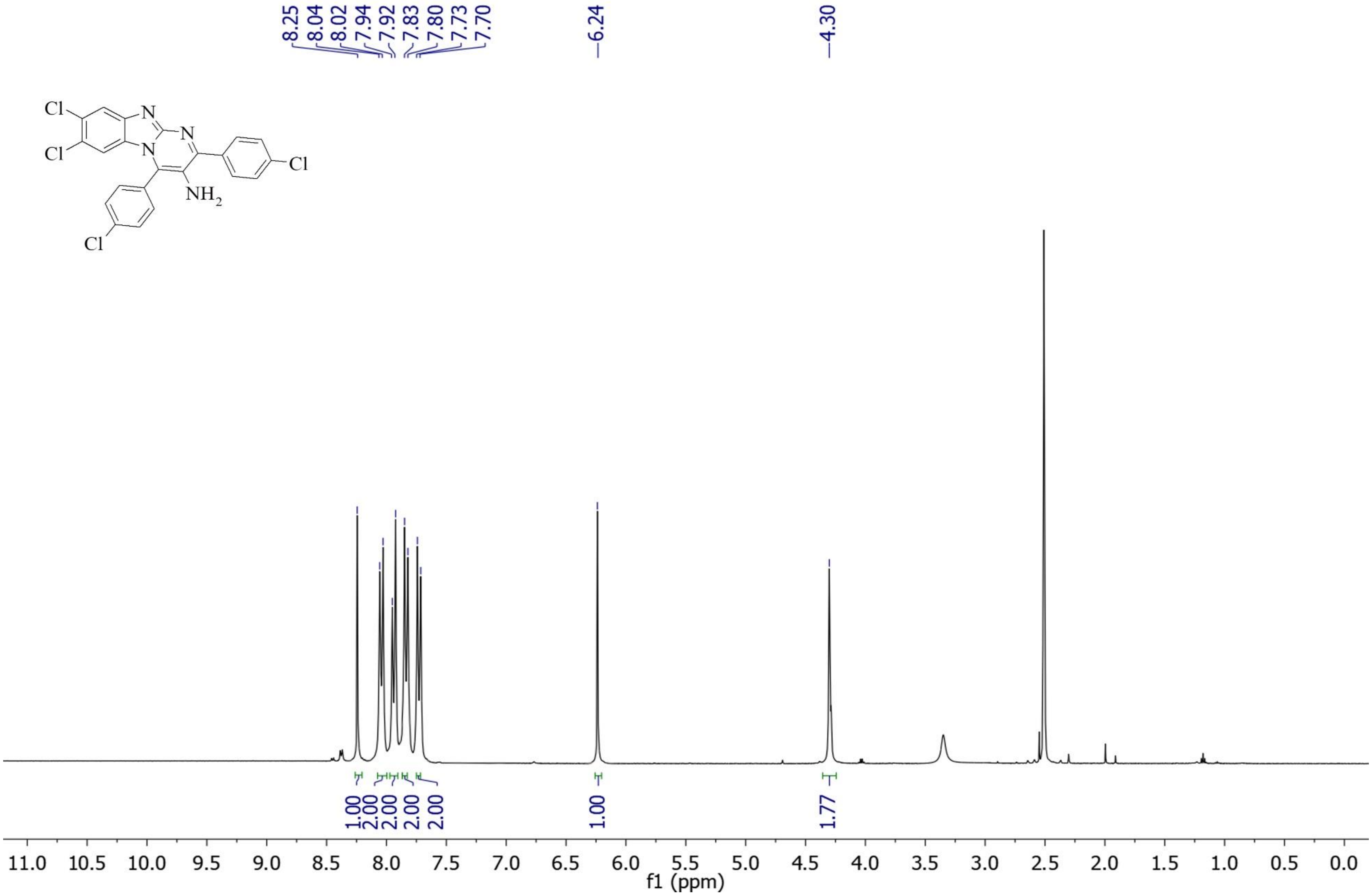

^{13}C NMR spectrum of 7,8-dichloro-2,4-bis-(4-chloro-phenyl)-benzo[4,5]imidazo[1,2-a]pyrimidin-3-ylamine (**3ad**)

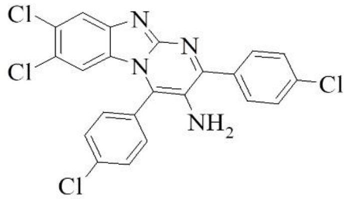

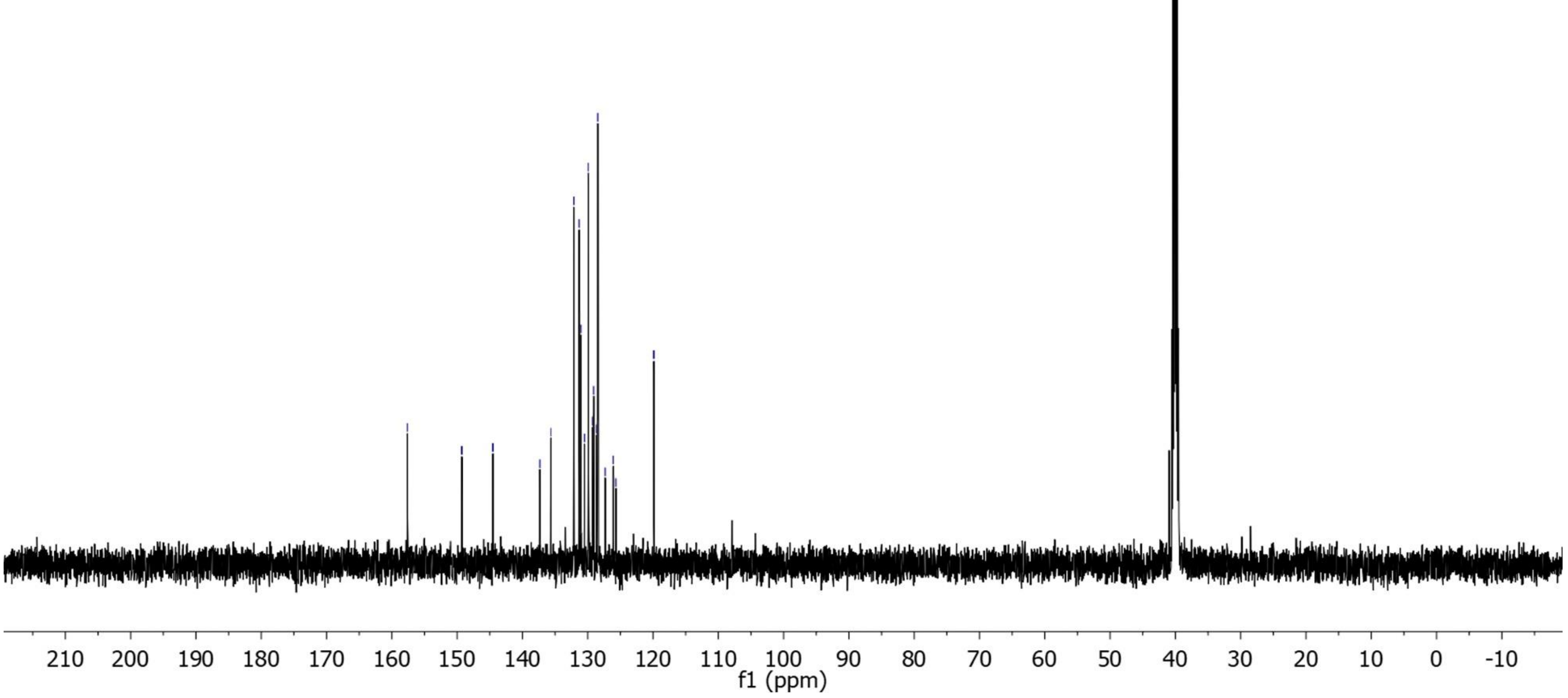

^{1}H NMR spectrum of 7,8-dichloro-2-(4-chloro-phenyl)-4-thiophen-2-yl-benzo[4,5]imidazo[1,2-a]pyrimidin-3-ylamine (**3ae**)

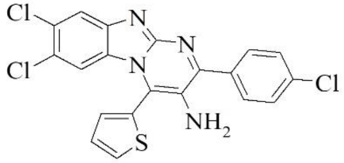

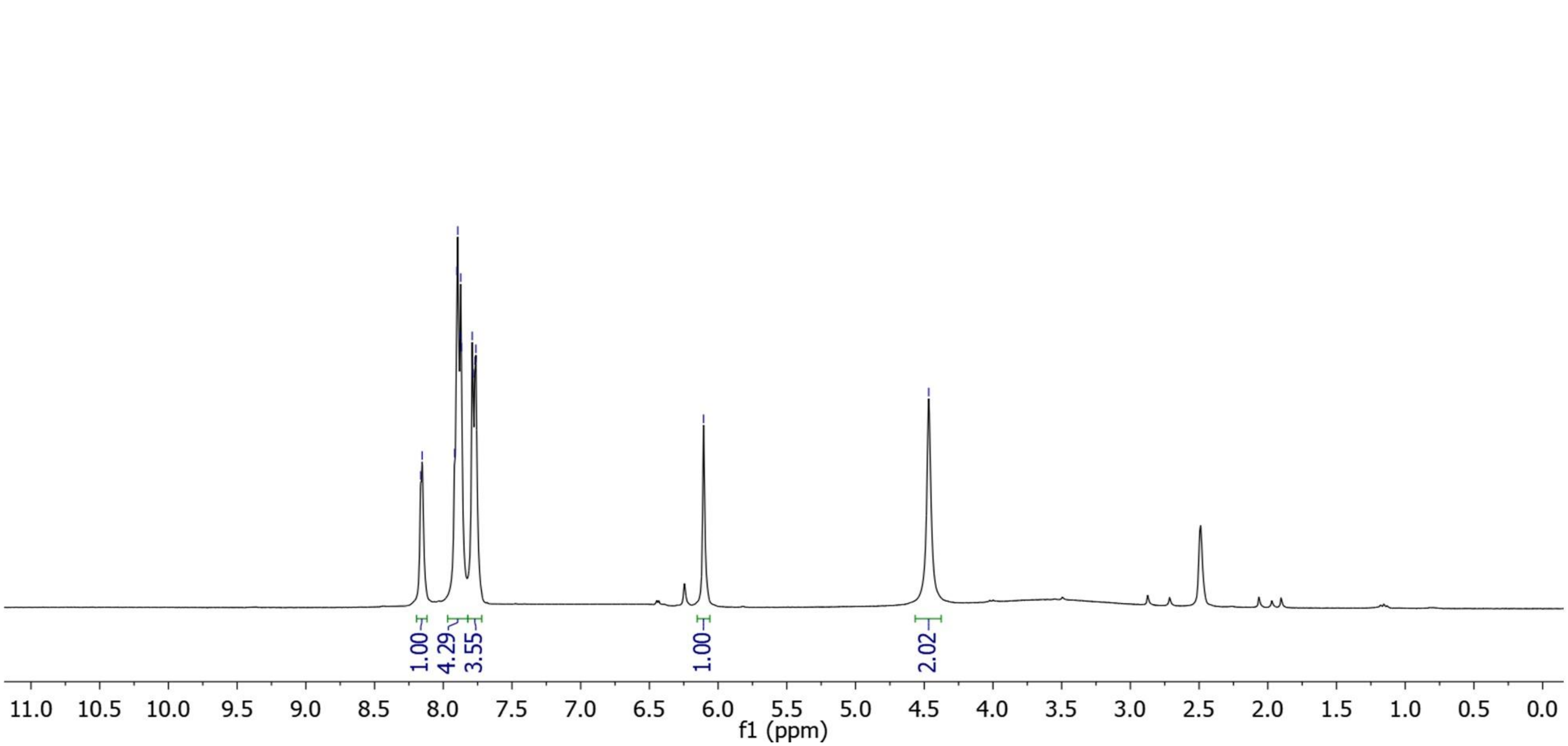

^{13}C NMR spectrum of 7,8-dichloro-2-(4-chloro-phenyl)-4-thiophen-2-yl-benzo[4,5]imidazo[1,2-a]pyrimidin-3-ylamine (**3ae**)

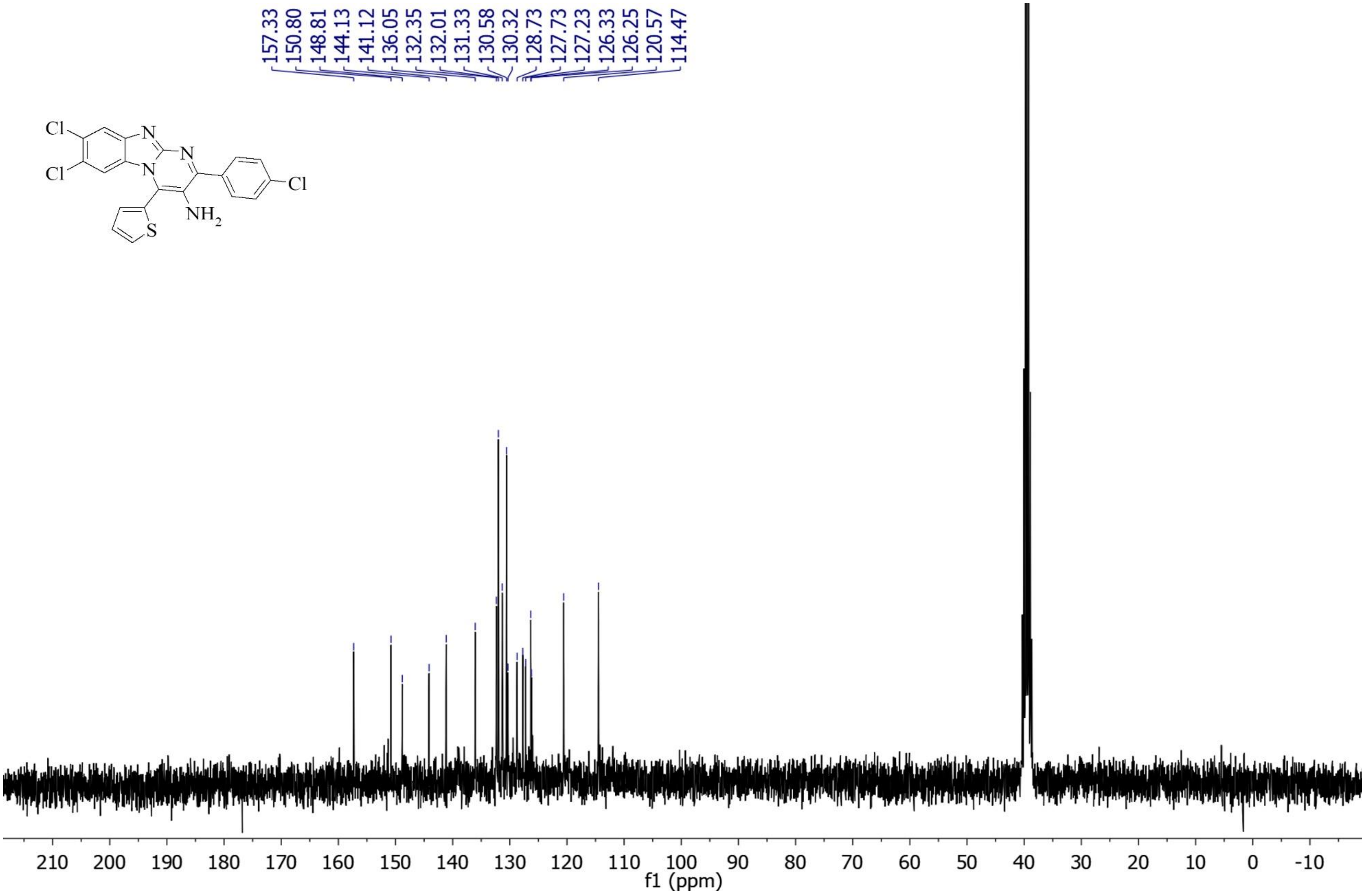

[1] H NMR spectrum of 2-(4-bromo-phenyl)-7,8-dichloro-4-phenyl-benzo[4,5]imidazo[1,2-a]pyrimidin-3-ylamine (**3af**)

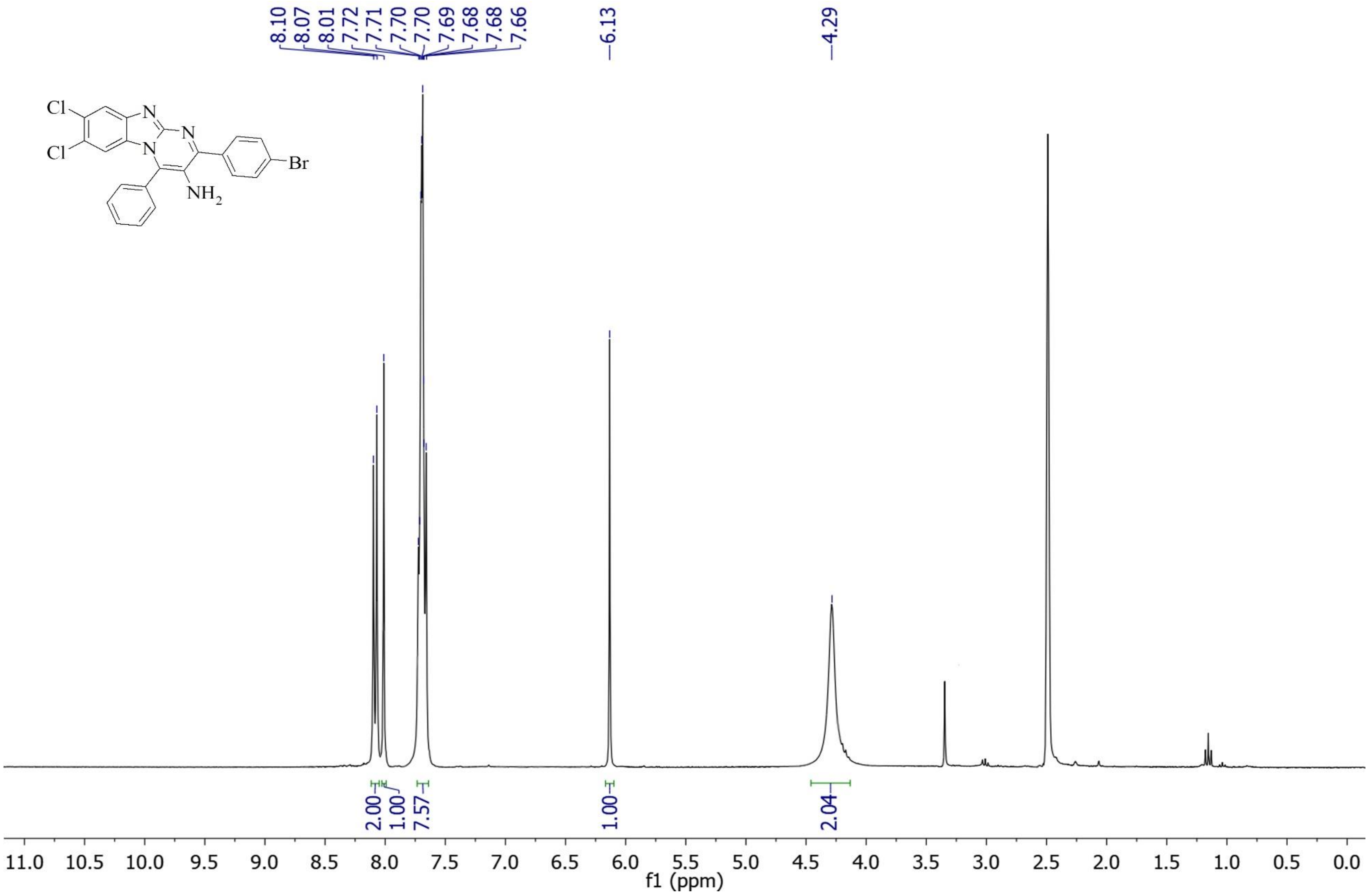

^{13}C NMR spectrum of 2-(4-bromo-phenyl)-7,8-dichloro-4-phenyl-benzo[4,5]imidazo[1,2-a]pyrimidin-3-ylamine (**3af**)

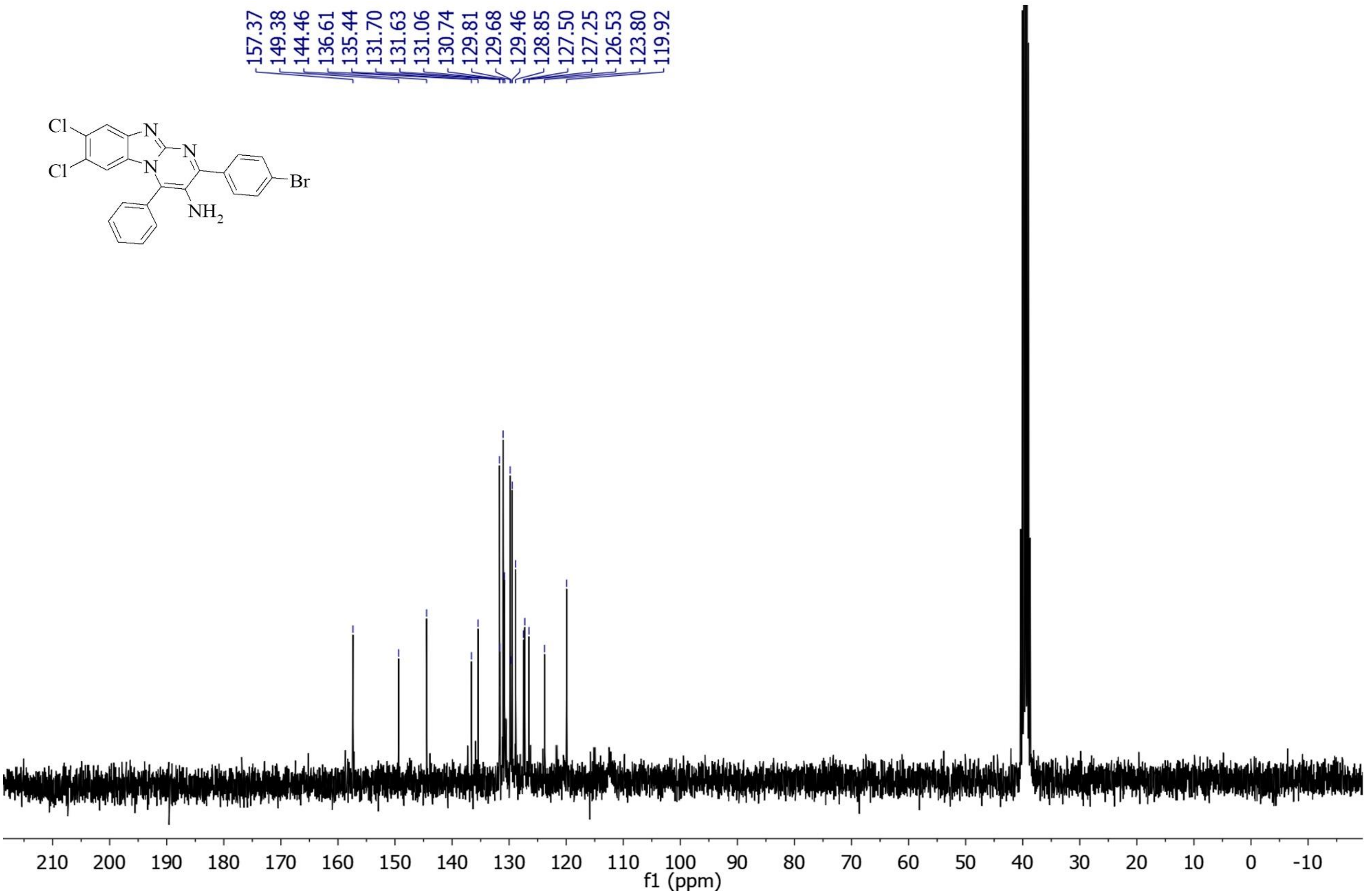

^{1}H NMR spectrum of 2-(4-bromo-phenyl)-7,8-dichloro-4-p-tolyl-benzo[4,5]imidazo[1,2-a]pyrimidin-3-ylamine (**3ag**)

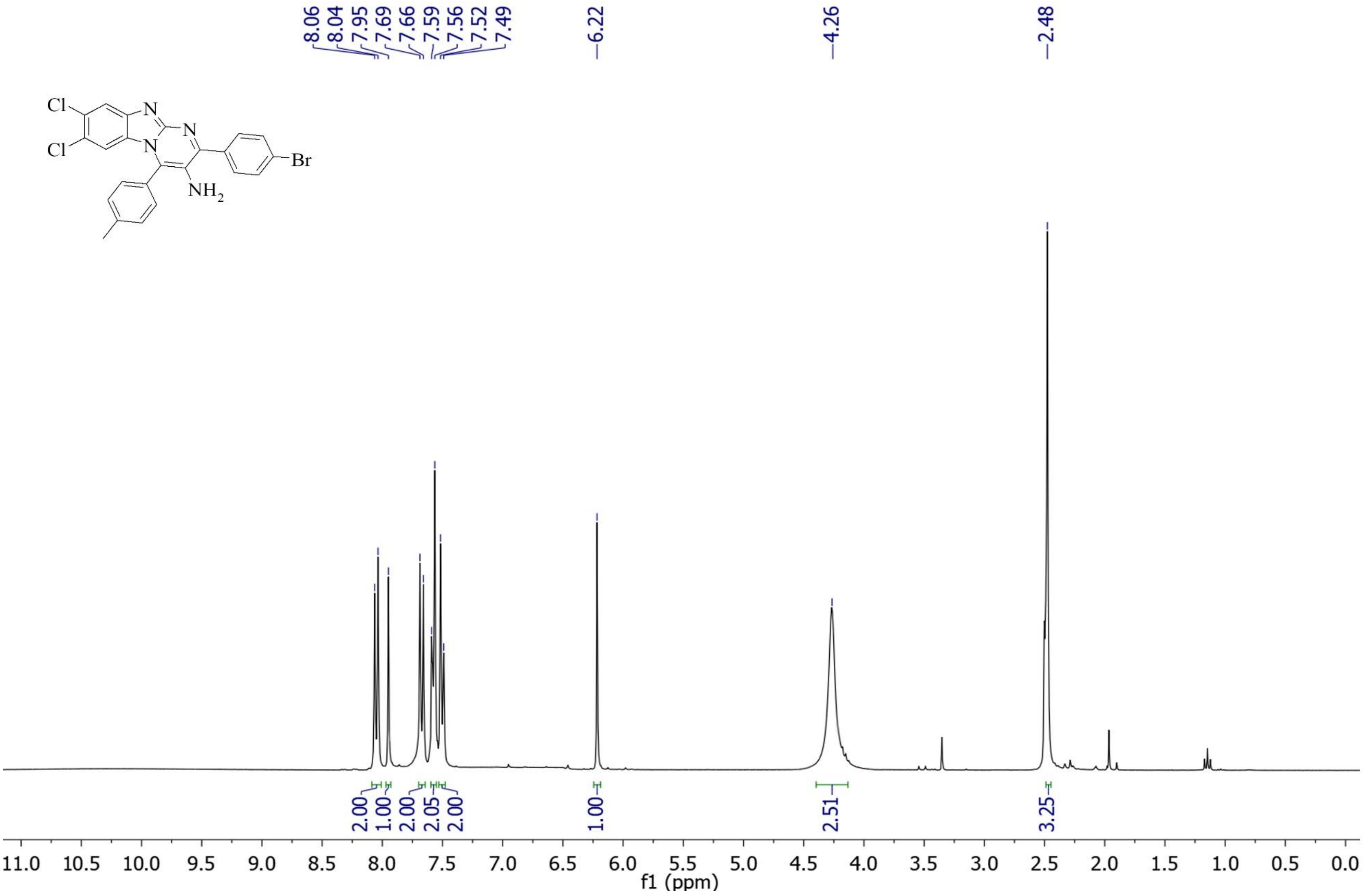

^{13}C NMR spectrum of 2-(4-bromo-phenyl)-7,8-dichloro-4-p-tolyl-benzo[4,5]imidazo[1,2-a]pyrimidin-3-ylamine (**3ag**)

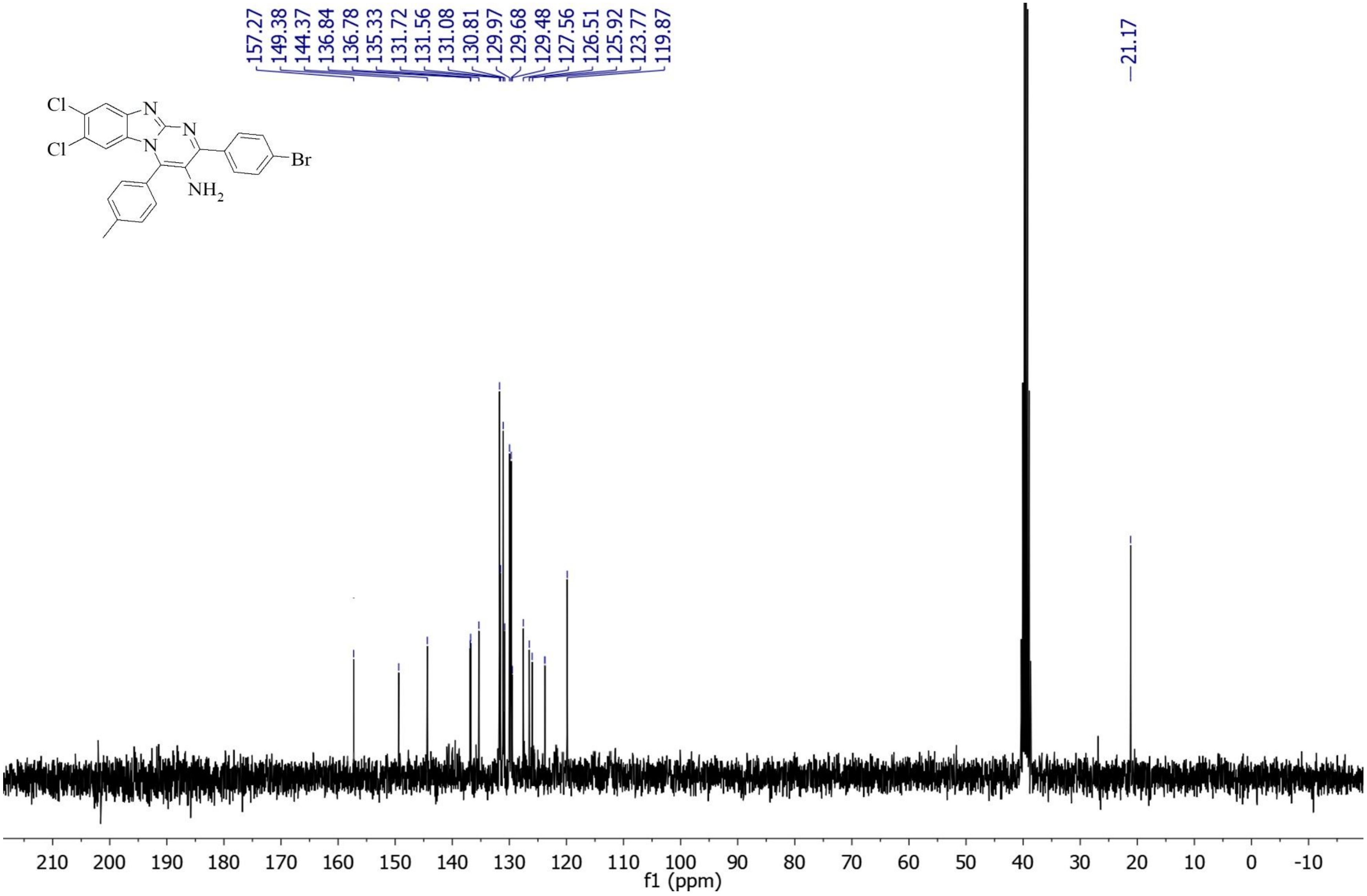

^{1}H NMR spectrum of 3-chloro-2,4-diphenyl-benzo[4,5]imidazo[1,2-a]pyrimidine (**4a**)

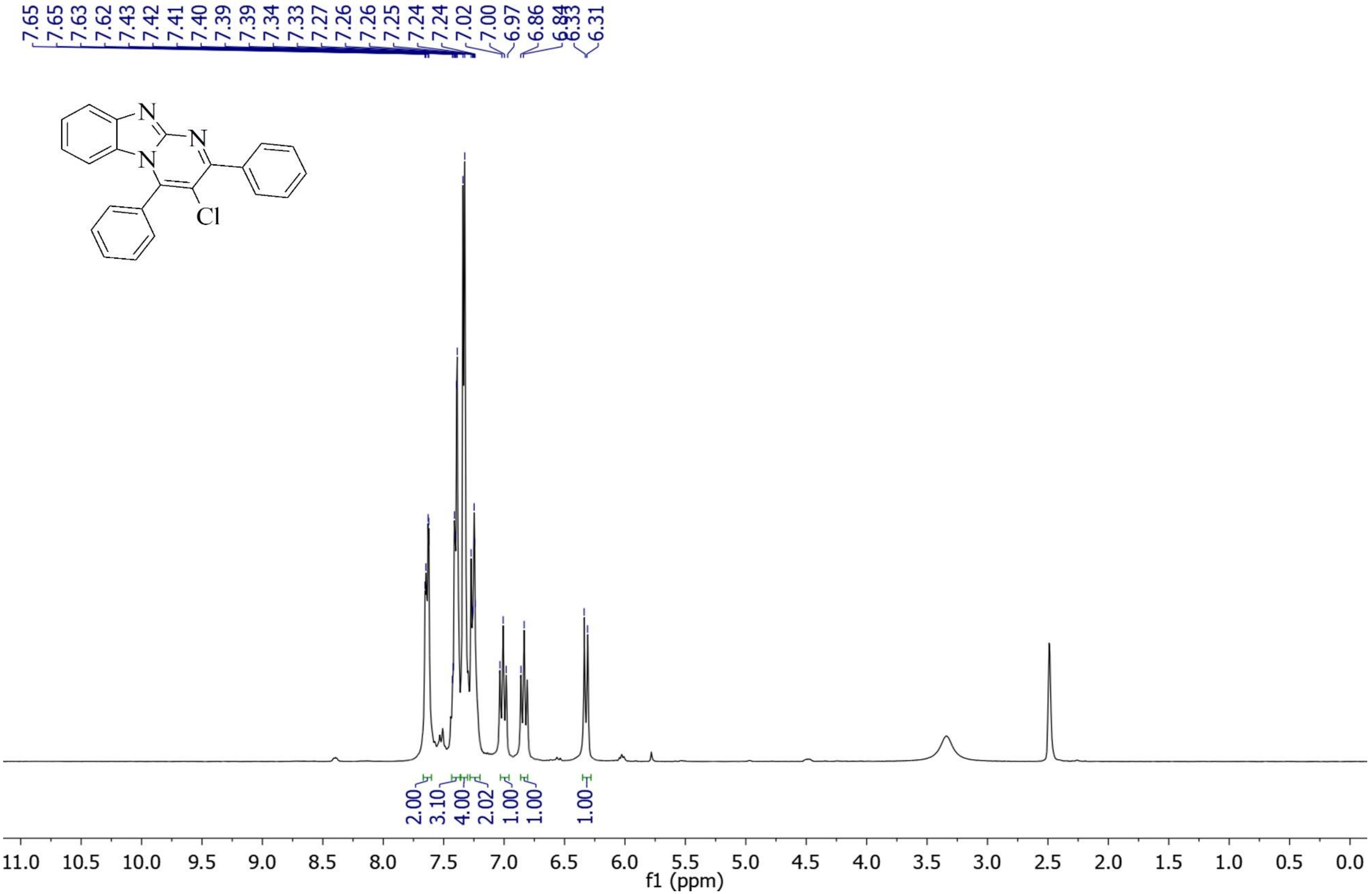

^{13}C NMR spectrum of 3-chloro-2,4-diphenyl-benzo[4,5]imidazo[1,2-a]pyrimidine (**4a**)

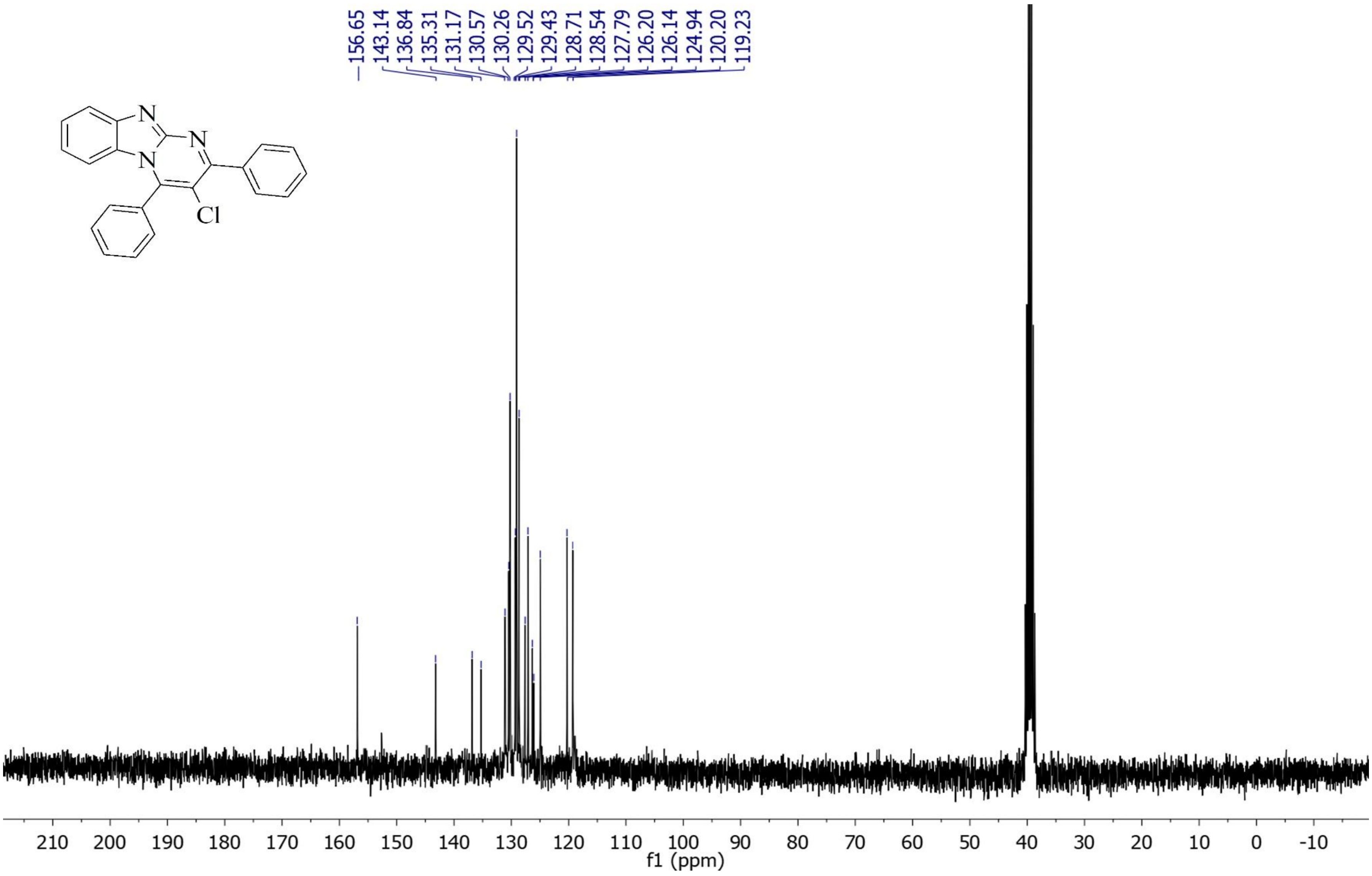

^{1}H NMR spectrum of 2,4-diphenyl-benzo[4,5]imidazo[1,2-a]pyrimidine (**6a**)

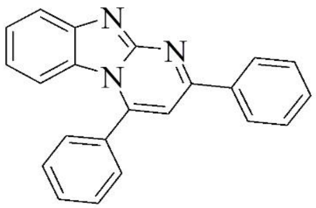

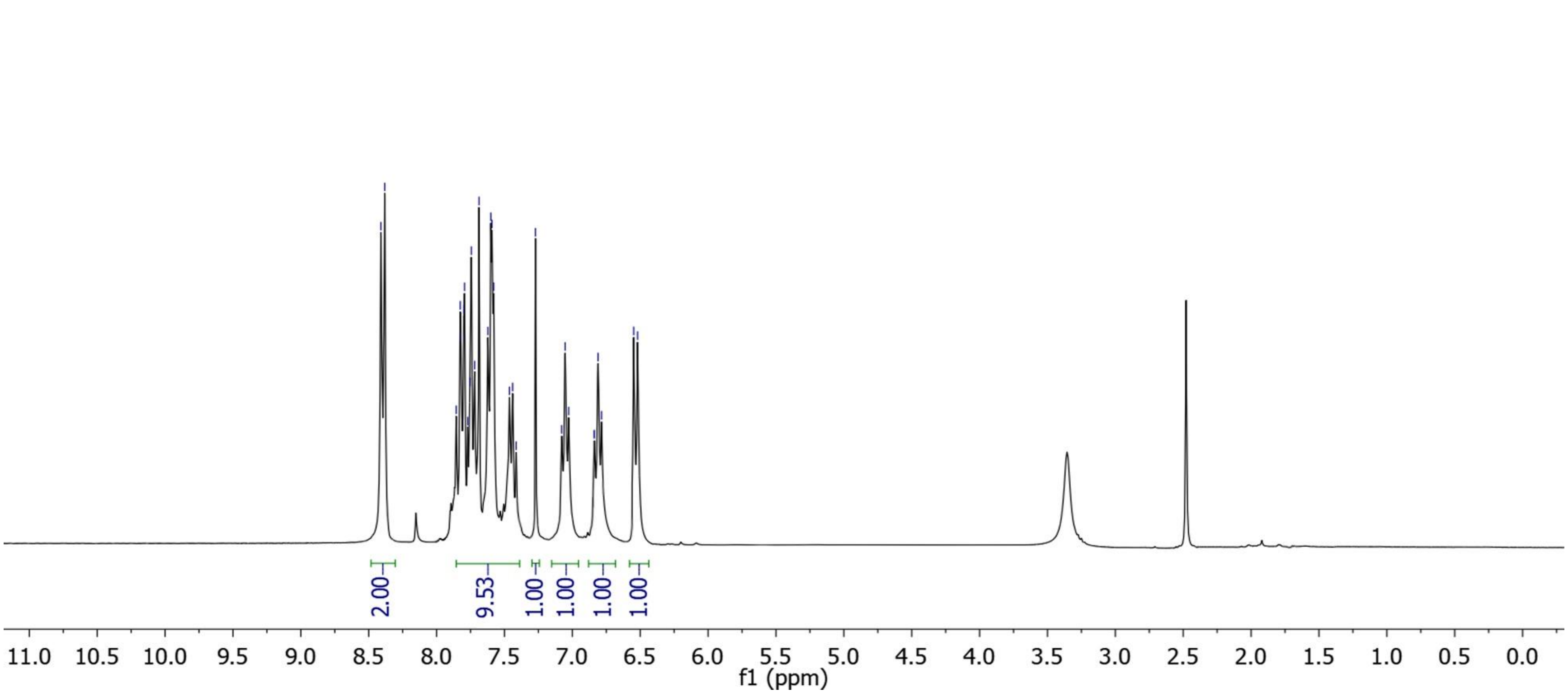

^{13}C NMR spectrum of 2,4-diphenyl-benzo[4,5]imidazo[1,2-a]pyrimidine (**6a**)

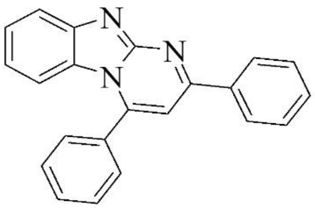

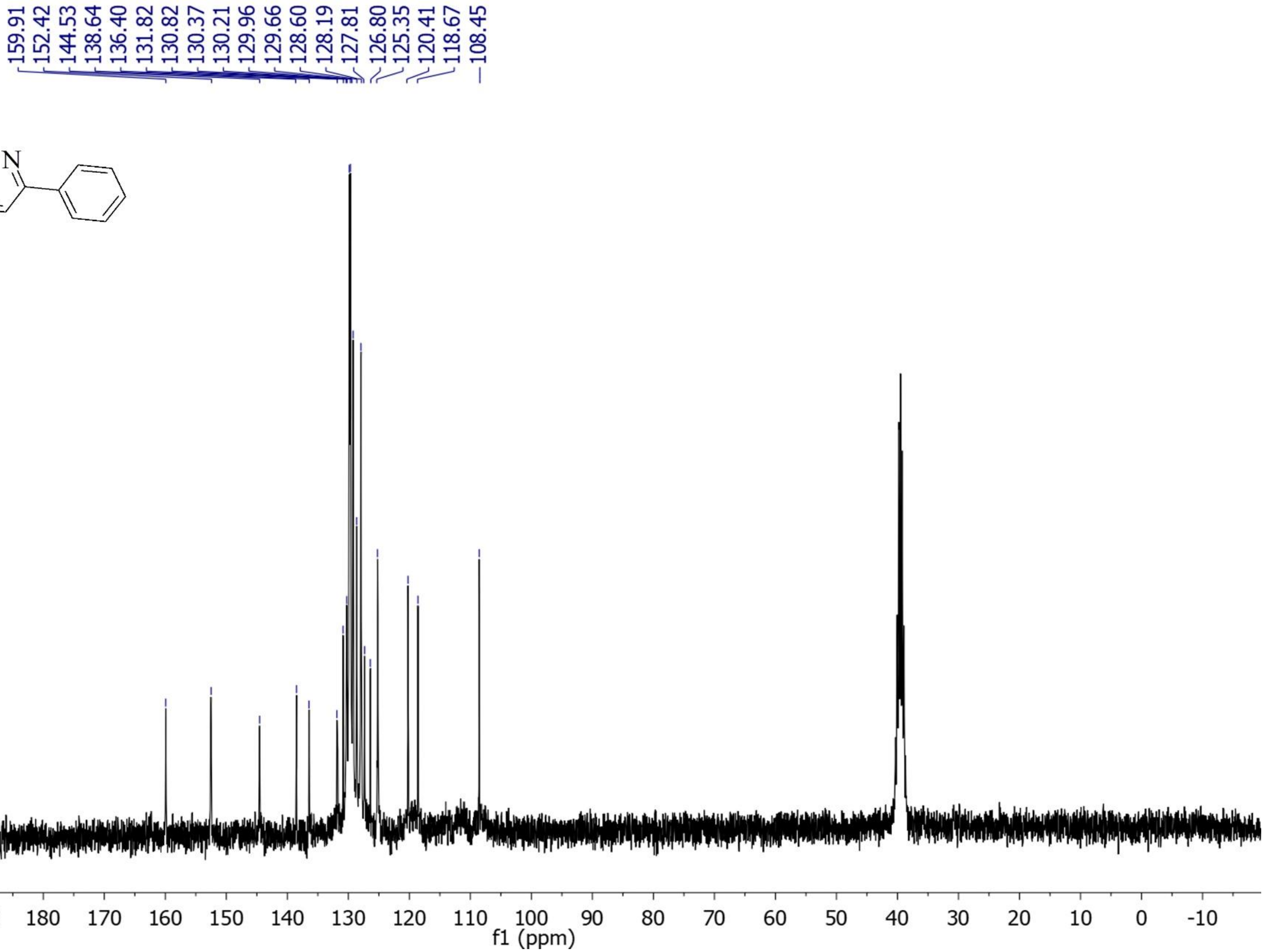

Supplement: Supplementary file 1 — Supplementary Information. [file 41598_2021_91473_MOESM1_ESM.pdf]
